# Metabolic Competition-Driven Antitumor Immunity of *glpX*-Deficient Engineered *Salmonella* via Glucose Deprivation and STING Activation in Bladder Cancer

**DOI:** 10.34133/cancomm.0060

**Published:** 2026-10-15

**Authors:** Leyang Wu, Xinyue Qiao, Wanru Wang, Jiahui Qiu, Xiao Dong, Liyuan Qiao, Chengxuan Lei, Xiaoxin Zhang, Ying Sun, Chenyang Li, Bohao Wang, Zengzheng Du, Heng Liu, Zhengbing Lv, Xiaofeng Jiang, Zichun Hua

**Affiliations:** ^1^ State Key Laboratory of Pharmaceutical Biotechnology, School of Life Sciences, Nanjing University, Nanjing, Jiangsu, P. R. China.; ^2^ Nanjing Generecom Biotechnology Co. Ltd., Nanjing, Jiangsu, P. R. China.; ^3^ Changzhou High-Tech Research Institute of Nanjing University and Jiangsu TargetPharma Laboratories Inc., Changzhou, Jiangsu, P. R. China.; ^4^ Department of Urology, Nanjing Drum Tower Hospital, Affiliated Hospital of Medical School, Nanjing University, Nanjing, Jiangsu, P. R. China.; ^5^ Yunnan Provincial Key Laboratory of Entomological Biopharmaceutical R&D, College of Pharmacy, Dali University, Dali, Yunnan, P. R. China.; ^6^ College of Life Sciences and Medicine, Zhejiang Provincial Key Laboratory of Silkworm Bioreactor and Biomedicine, Zhejiang Sci-Tech University, Hangzhou, Zhejiang, P. R. China.; ^7^ Faculty of Pharmaceutical Sciences, Xinxiang Medical University, Xinxiang, Henan, P. R. China.; ^8^ School of Biopharmacy, China Pharmaceutical University, Nanjing, Jiangsu, P. R. China.

## Abstract

**Background:** Bacteria-mediated cancer therapy is emerging as a promising approach due to its low cost, scalability, and sustained efficacy. While bacterial immunomodulation has been extensively studied, the role of bacterial metabolic competition, particularly for glucose, in tumor suppression remains unclear. We aimed to investigate how engineered *Salmonella* strains exploit metabolic competition to trigger antitumor immunity and improve therapeutic outcomes in bladder cancer models. **Methods:** Using genetic screening, we generated a gluconeogenesis-deficient *Salmonella* strain, the AISIH strain with the *glpX* gene knocked out (GLA1), with enhanced glucose uptake. In vitro and in vivo assays were performed to assess tumor cell glucose deprivation, mitochondrial stress, cytosolic mitochondrial DNA (mtDNA) release, and activation of the cGAS/STING pathway. An engineered strain, GLA2, was generated from the AISIH strain by knockout of the *glpX* gene and was subsequently further engineered to locally deliver an anti-tumor necrosis factor-α (TNF-α) nanobody under quorum-sensing control. Efficacy was evaluated in subcutaneous and orthotopic bladder cancer models, with analysis of tumor-infiltrating immune cells, dendritic cell (DC) maturation, and T cell priming. **Results:** GLA1 competitively depleted glucose in the bladder tumor microenvironment, inducing energy stress, mitochondrial damage, and mtDNA release, which noncanonically activated the STING pathway. This promoted type I interferon production, DC maturation, and antitumor immune responses. However, TNF-α-mediated vascular abnormalities limited efficacy. GLA2 normalized tumor vasculature via local anti-TNF-α delivery, enhancing nutrient competition and immune activation. Both strains markedly suppressed tumor growth, with GLA2 showing superior efficacy due to synergistic metabolic and immune reprogramming. **Conclusions:** This study demonstrates that bacterial competition for glucose is a pivotal mechanism of antitumor activity in bladder cancer, linking metabolic stress to STING-dependent immune activation. Engineered *Salmonella* strains that combine enhanced glucose uptake with local vascular normalization offer a robust platform for bladder cancer. These findings highlight a novel strategy to integrate microbial metabolic engineering with immune modulation, providing a foundation for precision bacteriotherapy in oncology.

## Introduction

The utilization of bacteria for antitumor therapy has a history that spans nearly a century, with extensive studies involving diverse bacterial agents, including the attenuated Bacille Calmette–Guérin (BCG) strain, as well as members of the *Salmonella* and *Listeria* genera [1–6]. Preclinical research has shown that these bacteria can selectively accumulate and proliferate within tumor tissues, markedly suppressing the tumor growth and prolonging the survival of tumor-bearing mice by inducing tumor cell death and remodeling the tumor immune microenvironment [1,5,7–10]. Among them, VNP20009, an attenuated *Salmonella* strain, which was derived from wild-type (WT) *Salmonella enterica* serovar Typhimurium (*S. Typhimurium*) 14028 through deletion of the *purI* and *msbB* genes and whose clinical safety was validated, exhibits substantially reduced systemic toxicity and enhanced biosafety while retaining its capacity for tumor-specific colonization and growth inhibition [1,3]. However, despite its promising preclinical efficacy, monotherapy with attenuated *Salmonella* has achieved only modest therapeutic outcomes in clinical trials [11]. Even when bacterial colonization within tumors is confirmed, patient responses remain suboptimal. In recent years, a variety of newly engineered attenuated *Salmonella* strains have been developed; however, their design strategies have largely focused on virulence gene knockout to increase safety [1,9,12]. Reports of chassis strains, which are intrinsically capable of increasing antitumor efficacy, remain scarce. Consequently, developing novel *Salmonella* strains that combine high biosafety with potent antitumor activity and pursuing synergistic engineering strategies grounded in a mechanistic understanding of bacteria–tumor interactions have emerged as critical research frontiers.

Metabolic reprogramming is a fundamental hallmark of tumor biology. Compared with normal cells, tumor cells exhibit markedly increased aerobic glycolysis, a phenomenon that is driven primarily by hypoxia-induced stress and mitochondrial respiratory dysfunction and collectively known as the Warburg effect [13]. Through glycolysis-dominated adenosine triphosphate (ATP) synthesis, tumor cells generate excessive amounts of lactate, which leads to the acidification of the tumor microenvironment, abnormal angiogenesis, and the suppression of antitumor immune activity [14–16]. This metabolic adaptation further enhances tumor tolerance to hypoxia while supporting the biosynthesis of lipids and other macromolecules via auxiliary metabolic pathways, such as the pentose phosphate pathway, thereby sustaining rapid proliferation. Because glycolysis yields far less ATP than oxidative phosphorylation does, tumor cells require large quantities of glucose to maintain energy homeostasis [17]. This metabolic vulnerability renders them particularly sensitive to metabolic interventions, creating new therapeutic opportunities. Current strategies that target tumor glucose metabolism include the inhibition of key enzymes, such as hexokinase 2 (HK2), and the glucose transporters GLUT1 and GLUT4 [13,18,19]. However, similar to conventional small-molecule inhibitors, these inhibitors often demonstrate poor tumor penetration, systemic toxicity, and a short duration of action. Although energy competition via artificial cells has emerged as an alternative approach, it remains technically complex and costly [20]. Therefore, achieving sustained and efficient energy deprivation of tumor cells remains a major challenge in the field of metabolic targeted therapy.

Bacteria, as rapidly proliferating organisms, have high energy demands. For example, a single *Escherichia coli* cell requires approximately 8 × 10^9^ ATP molecules to complete one cell division [21]. Extracellular bacteria acquire nutrients directly from their surrounding environment, whereas intracellular pathogens, such as *Salmonella*, sustain survival and replication by hijacking host cell metabolites [22]. On the basis of these insights, we hypothesized that upon tumor colonization, attenuated *Salmonella* cells could act as natural glucose competitors and indirectly suppress tumor growth by depleting glucose within the tumor microenvironment. Our findings confirmed that attenuated *Salmonella* and tumor cells engaged in substantial glucose competition, which involved both intracellularly retained and extracellular free bacteria. By using CRISPR/Cas9 technology, we constructed a series of *Salmonella* mutants with single deletions in gluconeogenesis- or glycolysis-related genes. Strikingly, gluconeogenesis-deficient strains presented increased antitumor activity, whereas glycolysis-deficient strains presented reduced efficacy. These results provide the genetic evidence that bacterial competition for glucose uptake represents a pivotal mechanism underlying bacteria-mediated antitumor effects. Building upon this discovery, we generated a highly glucose-competitive attenuated *Salmonella* strain, designated GLA1, which demonstrated superior antitumor efficacy in bladder tumor models relative to its parental strain. Mechanistically, GLA1-intensified glucose competition induced energy stress and mitochondrial damage to tumor cells. This process activated the stimulator of interferon gene (STING)/TANK binding kinase 1 (TBK1)/interferon regulatory factor 3 (IRF3) signaling axis, resulting in robust release of type I interferons (IFN-I), C-X-C motif chemokine ligand 10 (CXCL10), and interleukin-6 (IL-6) and promoting dendritic cell (DC) maturation. Notably, the metabolic reprogramming induced by bacterial competition for energy was accompanied by the aberrant up-regulation of tumor necrosis factor-α (TNF-α) expression, leading to abnormal tumor angiogenesis. To mitigate these adverse effects, we further engineered *Salmonella* and obtained GLA2, an improved strain that was designed to express a TNF-α-neutralizing nanoantibody (TNF-αnb) under controllable conditions. GLA2 achieved potent tumor suppression in a bladder cancer model through the following 3 mechanisms: (a) inhibition of tumor angiogenesis, (b) activation of the STING signaling pathway in tumor tissue, and (c) enhancement of both local and lymph node immune responses. Overall, this study expands our mechanistic understanding of bacteria-mediated antitumor activity and elucidates the central role of metabolic competition in microbiota–tumor interactions. Moreover, this study provides a theoretical and experimental foundation for the rational design of next-generation engineered microbial therapeutics that integrate metabolic, immunological, and vasculature-modulating functions for more effective cancer treatment.

## Materials and Methods

### Cells and strains

The mouse bladder cancer cell lines MB49, MB49-GFP (MB49 cells stably overexpressing green fluorescent protein), and MB49-Luxi (MB49 cells stably overexpressing a bioluminescent reporter) were maintained in our laboratory under standard culture conditions. Other cell lines maintained in our laboratory included the mouse breast cancer cell line 4T1, mouse lung cancer cell line LLC, mouse colorectal cancer cell line MC38, mouse melanoma cell line B16F10, mouse pancreatic cancer cell line Panc02, mouse hepatocellular carcinoma cell line H22, and mouse ascites tumor cell line S180. Bone marrow-derived dendritic cells (BMDCs) were generated from murine bone marrow monocytes by induction with IL-4 (catalog no. BK0246, Bioworld) and granulocyte-macrophage colony-stimulating factor (GM-CSF) (catalog no. HY-P7361, MedChemExpress) for 7 d, following established protocols.

The chassis strain AISIH used in this study was obtained as previously described [5]. Briefly, AISIH is an engineered attenuated *S. Typhimurium* strain developed for tumor-targeting applications. Novel single-gene-deficient mutants and engineered derivatives based on the AISIH strain were constructed in our laboratory, with detailed strain descriptions provided in Table S1. Single-gene knockout strains were generated via CRISPR-based genome editing as previously reported. Briefly, single-guide RNAs (sgRNAs) targeting the gene of interest were designed, and the target gene was deleted through CRISPR-mediated genome editing followed by homologous recombination. The resulting mutants were screened and verified by polymerase chain reaction (PCR) and DNA sequencing [5]. Genomic DNA from the resulting mutants was isolated and subjected to PCR amplification via the primers listed in Table S2, followed by agarose gel electrophoresis to confirm the targeted deletions. Engineered bacterial strains harboring expression plasmids were obtained by electroporating the desired plasmids into competent cells prepared from the corresponding recipient strains. To enable fluorescence visualization, the AISIH strain was transformed with a plasmid constitutively expressing red fluorescent protein (RFP), generating an AISIH-RFP strain for subsequent imaging analyses.

### Bacterial growth and colony morphology assays

Overnight cultures were inoculated into 3 mL of fresh Luria–Bertani (LB) medium and grown until reaching an OD₆₀₀ (optical density at 600 nm) of 0.6 to 0.8. The cultures were then diluted to 1 × 10^5^ colony-forming units (CFUs)/ml, and 10 μl of the resuspended bacterial suspension was added to each well containing 300 μl of LB in a honeycomb plate. Real-time growth monitoring was performed via a Bioscreen C system. LB medium alone served as the negative control, and each condition was assessed in triplicate. Growth curves were generated on the basis of optical density measurements collected at defined time intervals. For comparison of colony morphology among different bacterial strains, overnight cultures were adjusted to an identical OD₆₀₀ and serially diluted, and 2 μl of each dilution was spotted onto LB agar plates. Following overnight incubation, the plates were imaged to assess the morphological differences among the strains.

### Glucose uptake assay

Glucose uptake in MB49 mouse bladder cancer cells infected or not infected with *Salmonella* was assessed via the fluorescent glucose analog 2-NBDG (2-[*N*-(7-nitrobenz-2-oxa-1,3-diazol-4-yl)amino]-2-deoxy-d-glucose) (catalog no. MX4511, MKbio). Briefly, MB49 cells were cultured to the exponential growth phase, after which the medium was replaced with glucose-free, phenol red-free Dulbecco’s modified Eagle’s medium supplemented with 10 μM 2-NBDG. The cells were then cocultured with various *Salmonella* and their engineered strains at a multiplicity of infection (MOI) of 100 for the indicated durations. After incubation, the supernatant of the tumor cell culture was discarded to remove noninternalized bacteria, and the cell nuclei were stained with Hoechst dye (catalog no. KGA1804-10, KeyGEN BioTECH). Fluorescence images were acquired via a Zeiss fluorescence microscope to evaluate 2-NBDG uptake under different conditions. To evaluate 2-NBDG uptake by bacteria, overnight cultures were adjusted to the same OD₆₀₀, serially diluted, and added to medium containing 10 μM 2-NBDG. At the designated time points, fluorescence signals were quantified via a fluorometer by measuring emission at 542 nm (excitation: 488 nm). The fluorescence intensities at the dominant emission wavelengths were compared across strains.

### Transcriptomic analysis

The RNA sequencing dataset generated from MB49 mouse bladder cancer cells in this study (accession ID: GSE328957) was deposited in the National Center for Biotechnology Information (NCBI) Gene Expression Omnibus (GEO) database. The datasets generated and analyzed during this study are publicly accessible. Tumor cell RNA sequencing datasets generated from lung adenocarcinoma cell lines (accession ID: GSE286573) and HeLa cells (accession ID: GSE53280) were obtained from the GEO database. Principal components analysis (PCA) and heatmap visualizations of differential gene expression between tumor and normal tissues were generated via the R package “tinyarray”. Differential gene expression was performed via the DESeq2 package, applying significance thresholds of Benjamini–Hochberg-adjusted *P* < 0.05 and an absolute log_2_ fold change (log_2_FC) > 1 [23]. Kyoto Encyclopedia of Genes and Genomes (KEGG) pathway enrichment analysis was performed via the clusterProfiler R package (v4.0) to identify biological pathways significantly associated with the differentially expressed genes (DEGs) [24]. KEGG over-representation analysis (ORA) was performed using the DEGs as input with Benjamini–Hochberg correction, and pathways with *P*_adj_ < 0.05 were considered significantly enriched. Gene set enrichment analysis (GSEA) was performed using genes ranked by log₂FC, with a minimum gene-set size set to 10 and the maximum gene-set size set according to the gene-set collection used. GSEA was conducted using curated gene sets from the Molecular Signatures Database (MSigDB). Visualization of the enrichment results was carried out via bubble plots generated with ggplot2. The metabolic pathway enrichment of the single-cell RNA sequencing (scRNA-seq) data was analyzed through the ReactomeGSA R package [25]. For the transcriptomic analysis of MB49 cells, the attenuated *Salmonella* strain AISIH was cocultured with MB49 cells at an MOI of 100 for 6 h. The tumor cells were then harvested and submitted to OE Biotech Co. Ltd. (Shanghai, China) for RNA sequencing. Angiogenesis-related genes were classified according to their reported functions in the literature. The pro-angiogenic genes analyzed included vascular endothelial growth factor A (*Vegfa*), tumor necrosis factor (*Tnf),* prostaglandin-endoperoxide synthase 2 (*Ptgs2*), AKT serine/threonine kinase 3 (*Akt3*), polo-like kinase 2 (*Plk2*), heme oxygenase 1 (*Hmox1*), and GATA binding protein 2 (*Gata2*), whereas the anti-angiogenic genes included transforming growth factor β receptor 3 (*Tgfbr3*), thrombospondin 1 (*Thbs1*), forkhead box O1 (*Foxo1*), collagen type XVIII α1 chain (*Col18a1*), and TIMP metallopeptidase inhibitor 3 (*Timp3*). Subsequent bioinformatic analyses were carried out following the strategy described above.

### Protein expression assay

To evaluate the in vivo expression of target proteins driven by different promoters—including the quorum-sensing promoter pLux and the constitutive promoter pJ23100—the luxCDABE operon was used as a bioluminescent reporter. The corresponding engineered bacterial strains were administered intraperitoneally to MB49 tumor-bearing mice. Bioluminescence imaging was performed via an IVIS imaging system (PerkinElmer) at multiple time points following administration to monitor bacterial protein expression dynamics in vivo. Major organs were collected postnecropsy and imaged to further assess strain-specific reporter expression.

### Apoptosis and proliferation assays

Overnight cultures of attenuated *Salmonella* strains were cocultured with MB49 tumor cells in media containing low, moderate, or high glucose concentrations at an MOI of 100 for the indicated durations. All the cells from the culture system were then collected and washed 1 to 2 times with ice-cold 1× binding buffer. The cells were resuspended in 400 μl of 1× binding buffer, mixed with Annexin V–fluorescein isothiocyanate (FITC) (homemade), and incubated at room temperature in the dark for 30 min. Propidium iodide (PI) was subsequently added for nuclear staining, and apoptosis was quantified via flow cytometry (FCM). To assess tumor cell proliferation, different bacterial strains were continuously cocultured with equal numbers of MB49-GFP cells at an MOI of 100. The GFP fluorescence intensity in the culture system (excitation: 488 nm; emission: 525 nm) was measured via a microplate reader (BioTek) to evaluate relative proliferation over time.

### Real-time quantitative PCR

Real-time quantitative PCR (qPCR) was performed as previously described [5]. Briefly, total RNA was extracted via FreeZol (catalog no. R711-01, Vazyme), and cDNA was synthesized via a reverse transcription kit (catalog no. M16315, Thermo Fisher Scientific). qPCR was carried out via the use of cDNA templates and a commercial qPCR mixture (catalog no. Q111-02, Vazyme). Each experiment included 3 to 4 biological replicates. Relative mRNA expression levels were calculated via the 2^−ΔΔ*C*t^ method, with β-actin serving as the internal reference gene. Gene expression analyses included markers associated with cellular energy metabolism [solute carrier family 2 member 1 (*Slc2a1*), solute carrier family 2 member 4 (*Slc2a4*), phosphofructokinase, platelet (*Pfkp*), hexokinase 2 (*Hk2*), cytochrome c oxidase subunit 6B1 (*Cox6b1*), and cyclin B1 (*Ccnb1*)] and indicators of STING pathway activation [interferon-β1 (*Ifnb1*), C-X-C motif chemokine ligand 10 (*Cxcl10*), and interleukin-6 (*Il-6*)]. The primer sequences are provided in Table S2.

### Mitochondrial damage assessment

For the quantification of mtDNA in the cytosolic extracts, freshly harvested MB49 cells were resuspended in 500 μL of buffer containing 150 mM NaCl, 50 mM Hepes (pH 7.4), and 20 μg/ml digitonin (Sigma, D141). The cell suspensions were incubated on a rotating shaker for 10 min to selectively permeabilize the plasma membrane, followed by centrifugation at 800×*g* for 5 min, which was repeated 3 times to pellet intact cells. The resulting supernatants were transferred to new tubes and centrifuged at 25,300*g* for 10 min to remove residual cellular debris, yielding cytosolic fractions devoid of nuclear, mitochondrial, and endoplasmic reticulum contamination. Protein concentrations were normalized via a bicinchoninic acid (BCA) protein assay kit (catalog no. BL521A, Biosharp). DNA was extracted from equal amounts of cytosolic fractions via the QIAquick Nucleotide Removal Kit (catalog no. 28306, Qiagen) according to the manufacturer’s instructions. mtDNA levels were quantified via a StepOne real-time PCR system with the mtDNA-specific primers listed in Table S2. For transmission electron microscopy (TEM) analyses of mitochondrial morphology, MB49 cells were incubated with different bacterial strains or the phosphate-buffered saline (PBS) control in medium containing 1 mM glucose for 6 h (MOI 100). The tumor cells were then processed following established fixation and sample preparation procedures [26] and submitted to Sevicebio Company (Wuhan, China) for TEM processing and imaging.

### Assessment of intracellular reactive oxygen species

The intracellular reactive oxygen species (ROS) levels were measured via a 2',7'-dichlorodihydrofluorescein diacetate (DCFH-DA) fluorescent probe (Solarbio, D6470, Beijing, China). MB49 cells were seeded in 6-well plates and allowed to adhere. The cells were then infected with the indicated bacterial strains at an MOI of 100 in medium containing 1 mM glucose. After 3 h of infection, the samples designated for confocal laser scanning microscopy (CLSM) were washed and incubated with serum-free RPMI 1640 medium containing 5 μM DCFH-DA and Hoechst 33342 for 20 min. The cells were subsequently washed 3 times with PBS and imaged via a fluorescence inverted microscope. For FCM analysis, cells were treated under identical conditions and digested to obtain single-cell suspensions, and analysis was performed via a BD FACSCanto II.

### Mitochondrial membrane potential assay

The mitochondrial membrane potential (MMP) was assessed via Mitochondrial Membrane Potential Assay Kit with tetramethylrhodamine ethyl ester (TMRE, catalog no. C2001S, Beyotime). Briefly, MB49 cells were seeded into 6-well plates. After cell attachment, the culture medium was replaced with medium containing 1 mM glucose. The tumor cells were then infected with different bacterial strains at an MOI of 100. After 3 h, the medium was replaced with detection buffer containing TMRE according to the manufacturer’s instructions. Following a 20-min incubation, the cells were washed 3 times with PBS, collected as a single-cell suspension, and analyzed via a flow cytometer (BD Biosciences).

### Cell viability assay

Cell viability was assessed using a Cell Counting Kit-8 (catalog no. A311-01, Vazyme) according to the manufacturer’s instructions. Briefly, cells were seeded into 96-well plates. After the indicated treatments, 10 μl of CCK-8 solution was added to each well, followed by incubation at 37 °C for 1 to 2 h. The absorbance was measured at 450 nm using a microplate reader. Cell viability was calculated after subtraction of the background absorbance from blank wells. Results were expressed as a percentage relative to the control group.

### Cytokine quantification

Supernatants collected from MB49 cells treated with different bacterial strains (MOI 100, 2 × 10^5^ cells/ml in medium added with 1 mM glucose) were analyzed for cytokine production via commercial enzyme-linked immunosorbent assay (ELISA) kits according to the manufacturers’ instructions, including assays for IFN-β (catalog no. EK2236, Multi Sciences), IL-6 (catalog no. BY-EM220188, BYabscience), and CXCL10 (catalog no. EK268, Multi Sciences). For in vivo cytokine assessment, MB49 tumor-bearing C57BL/6 mice received the indicated treatments. Tumors were harvested 3 d post-treatment, homogenized, and analyzed via ELISA kits to quantify intratumoral IFN-β following the manufacturer’s protocol.

### Western blotting

For tumor cell lysates, cells treated with the indicated bacterial strains were collected and homogenized in ice-cold RIPA lysis buffer supplemented with protease inhibitors (catalog no. PK10014, Proteintech) and phosphatase inhibitors (catalog no. RM02997, ABclonal). Protein concentrations were quantified via a BCA assay kit according to the manufacturer’s protocol. Total protein extracts (12 to 45 μg) were mixed with 5× loading buffer (catalog no. C516031, Sangon Biotech). For bacterial lysates, cultures were adjusted to OD₆₀₀ = 1.0, pelleted by centrifugation, resuspended in PBS, and mixed with 5× loading buffer. After SDS-PAGE and protein transfer, the membranes were incubated overnight at 4 °C with the following primary antibodies: anti-phosphorylated STING (p-STING), STING, phosphorylated TBK1 (p-TBK1), TBK1, phosphorylated IRF3 (p-IRF3), IRF3, glyceraldehyde-3-phosphate dehydrogenase (GAPDH), and tubulin. The membranes were washed 3 times with tris-buffered saline with Tween 20 (TBST) and incubated with horseradish peroxidase (HRP)-conjugated goat anti-rabbit or anti-mouse immunoglobulin G (IgG) secondary antibodies. The protein bands were visualized via chemiluminescence. Detailed antibody information is provided in Table S3.

To detect TNF-αnb expression and secretion by the GLA2 strain, high-density cultures of GLA2 were collected. The intracellular and extracellular protein fractions were harvested following established procedures [8]. After normalization to equal protein amounts, samples were subjected to SDS-PAGE and immunoblotting using an anti-FLAG primary antibody (catalog no. F1804, Sigma) to examine the expression and secretion of the target protein across strains. Before analyzing bacterial pellets and secreted proteins, protein concentrations were quantified using a BCA assay, and equal amounts of protein were loaded for each sample.

### Animal models

All animal experiments were approved by the Animal Care and Use Committee of Nanjing University (IACUC-2003167) and performed in accordance with institutional ethical guidelines. Female C57BL/6J mice (6 to 8 weeks old) were obtained from Changzhou Cavens Animal Company. MB49 cells (2 × 10^5^ per mouse) were injected subcutaneously into the right axillary region. When the tumors reached 80 to 120 mm^3^, the mice were treated intraperitoneally with the indicated bacterial strains. The tumor dimensions were measured every 1 to 2 d via calipers, and the tumor volume (*V*, mm^3^) was calculated as *V* = *a*^2^ × *b* × 0.52, where *a* is the smaller radius and *b* is the larger radius. Tumor growth curves were generated on the basis of serial measurements. Body weight was monitored throughout the experiment. The tumor volume doubling time (TVDT) was calculated as follows: TVDT = (Δ*t* × ln 2)/ln(*V*₂/*V*₁). Survival curves were generated from daily monitoring of tumor-bearing mice. The mice were euthanized upon signs of distress—such as pain, lethargy, or tumor necrosis—or when humane endpoints were reached (tumor mass ≥10% of body weight).

To eliminate TNF-α within tumors, 0.5 mg per mouse of anti-mouse TNFα-InVivo (catalog no. A2124, Selleck) or 5 mg/kg TNF-αnb (homemade) was administered intraperitoneally. To establish the orthotopic bladder cancer model, MB49 cells were instilled intravesically into 8-week-old female C57BL/6J mice. The mice were anesthetized, the bladder was emptied, and 2 × 10^5^ MB49 cells were introduced into the bladder and retained for >40 min. On day 6 after tumor cell instillation, a biotin tracer (catalog no. DD1210, Vazyme; 150 mg/kg) was injected intraperitoneally, and in vivo bioluminescence imaging was used to monitor orthotopic tumor growth via an IVIS imaging system (PerkinElmer).

The body weights and clinical symptoms of the mice were monitored throughout the study to ensure their welfare. Humane endpoints were applied when the tumor mass reached ≥10% of body weight or when clinical conditions met veterinary criteria. For hematoxylin and eosin (H&E) and TUNEL (terminal deoxynucleotidyl transferase-mediated deoxyuridine triphosphate nick end labeling) staining, tumor and normal tissues were collected postmortem, fixed in paraformaldehyde, and submitted to Servicebio (Wuhan, China) for histological processing.

### FCM analysis

To investigate changes in the tumor microenvironment, tumor-bearing mice were euthanized following different treatments, and tumors were harvested. Tumors were digested in collagenase-containing medium for 45 min to prepare single-cell suspensions, which were then passed through a 40-μm cell strainer to remove cell clumps. The cells were stained with a fixable viability dye (catalog no. 564407, BD Biosciences) and incubated at 4°C in the dark for 30 to 45 min. The surface markers were stained with the following anti-mouse antibodies: CD45–BV510, CD3–FITC, CD4–peridinin chlorophyll protein (PERCP)–CY5.5, CD8–phycoerythrin (PE)–CY7, CD69–BV605, CD11b–FITC, CD11c–PE–CY7, CD103–PERCP–CY5.5, CD86–PE, and F4/80–BV421. The intracellular markers were stained with the following anti-mouse antibodies: granzyme B (GzmB)–allophycocyanin (APC), Foxp3–PE, Ki67–BV421, and CD206–APC. Detailed antibody information is provided in Table S3. A permeabilization and fixation kit, BD Pharmingen Transcription Factor Buffer Set (catalog no. 562574, BD Biosciences), was used for cell fixation and permeabilization according to the manufacturer’s instructions.

For the detection of BMDC maturation, tumor cells were seeded at a density of 4 × 10^6^ cells/ml with different strains for 8 h. The collected supernatant was mixed with normal medium (1:1) and used to culture 2 × 10^5^ DC cells for 24 h. The anti-mouse antibodies CD11c-APC (catalog no. G0093, Selleck), CD80-FITC (catalog no. G0211, Selleck), and CD86-PE (catalog no. 12-0862-82, Invitrogen) were used for surface marker staining.

### Statistical analysis

Statistical analyses were conducted via GraphPad Prism 7 (GraphPad Software). Between-group differences were assessed via 2-tailed *t* tests. For experiments involving 3 or more groups, one-way analysis of variance (ANOVA) was performed, followed by Dunnett’s multiple comparison test to determine statistical significance. Quantitative analysis of fluorescence images was performed via ImageJ. The figures in the text were created via Adobe Illustrator. The results are presented as the means ± standard deviations (SDs).

## Results

### Antitumor bacteria induce aberrations in tumor cell glucose metabolism

The intravenous or intraperitoneal administration of oncolytic bacteria enables their selective accumulation and rapid proliferation within tumors, with intratumoral bacterial titers often reaching 10^9^ CFU/g. Bacterial proliferation depends on the uptake of glucose as an energy source [21]. In general, extracellular bacteria derive energy from the extracellular milieu, whereas intracellular bacteria acquire energy through host cell metabolites. *S. Typhimurium*, for example, can absorb glucose from host cells and utilize glycolysis-derived energy for intracellular replication [22]. On the basis of these observations, we hypothesized that antitumor bacteria might directly interfere with tumor cell glucose metabolism. To test this hypothesis, we analyzed transcriptomic datasets from coculture systems of antitumor bacteria and tumor cells, including *E. coli* with the human non-small cell lung cancer cell line A549 [27], *E. coli* with the human lung adenocarcinoma cell line H2009 [27], and *S. Typhimurium* with the human cervical carcinoma cell line HeLa [28]. These bacterial species are known to colonize tumors and, importantly, exhibit well-documented anticancer activity [1,11]. KEGG pathway enrichment analysis coupled with GSEA revealed significant activation of apoptosis-related pathways across diverse tumor–bacteria coculture systems (Figs. S1 and S2). These observations are consistent with previous findings showing that oncolytic bacteria can directly trigger tumor cell apoptosis [29]. Notably, glucose metabolism-related pathways were markedly altered in tumor cells following bacterial infection (Fig. S1). These findings suggest that tumor cells adapt to bacterial challenge by reprogramming glucose metabolism to enhance energy acquisition. Further GSEA revealed that coculture with both *E. coli* and *S. Typhimurium* significantly activated glycolysis-related pathways while suppressing oxidative phosphorylation-related pathways in tumor cells (Fig. S2). These findings indicate that bacterial infection leads to the amplification of the Warburg effect—favoring glycolysis and lactate production while attenuating oxidative phosphorylation—to meet heightened energy demands. Collectively, these results demonstrate that oncolytic bacteria can reprogram tumor cell glucose metabolism.

Different tumor types exhibit variability in their glucose dependencies; for example, bladder cancer shows heightened sensitivity to nutrient deprivation, which suggests increased susceptibility to metabolic perturbation [14]. Importantly, BCG therapy, a microbe-based approach, has been approved as a first-line treatment for bladder cancer, making this cancer type a promising candidate for further clinical exploration of oncolytic bacteria [2]. To further elucidate this possibility, we performed transcriptomic profiling of mouse bladder cancer cells (MB49) following a 6-h coculture with attenuated *Salmonella* AISIH (Fig. 1A). The AISIH strain is an engineered attenuated *S. Typhimurium* strain that has excellent biosafety, as it exhibits strong immunogenicity only at high bacterial density or upon activation [5,30]. PCA revealed distinct transcriptional separation between the AISIH-treated group and the saline-treated control group, with the tight intragroup clustering indicating high data quality and reproducibility (Fig. 1B). Volcano plot analysis revealed 421 up-regulated and 266 down-regulated genes compared with those in the control (Fig. 1C). Consistent with prior results, GSEA based on MSigDB demonstrated significant activation of apoptosis pathways (Fig. S3A and B). Immune-related pathways were also prominently enriched, suggesting that a robust immune response was elicited by the AISIH strain in MB49 cells (Fig. S3C). Notably, compared with the saline group, MB49 cells in the AISIH group exhibited enrichment of the glycolytic pathway, whereas the oxidative phosphorylation and tricarboxylic acid (TCA) cycle pathways were depleted, indicating that AISIH induces profound metabolic dysregulation in MB49 cells, similar to that observed in other tumor cell types (Fig. 1D to G and Figs. S1 and S2). KEGG pathway enrichment analysis further revealed substantial modulation of cellular metabolism, particularly glucose, pyruvate, and galactose metabolism (Fig. 1H). Specifically, genes promoting glucose import and glycolysis were markedly up-regulated in AISIH strain-treated cells, whereas genes involved in the TCA cycle and oxidative phosphorylation were down-regulated (Fig. 1I). These findings suggest that tumor cells adapt to bacterial competition for glucose by increasing glycolytic flux and reducing their reliance on oxidative phosphorylation to sustain energy production [15,16]. Together, these findings indicate that oncolytic bacteria profoundly reprogram tumor glucose metabolism, driving a metabolic shift from oxidative phosphorylation toward glycolysis. This metabolic remodeling highlights a potential mechanism of bacteria-mediated anticancer activity.

**Fig. 1.**
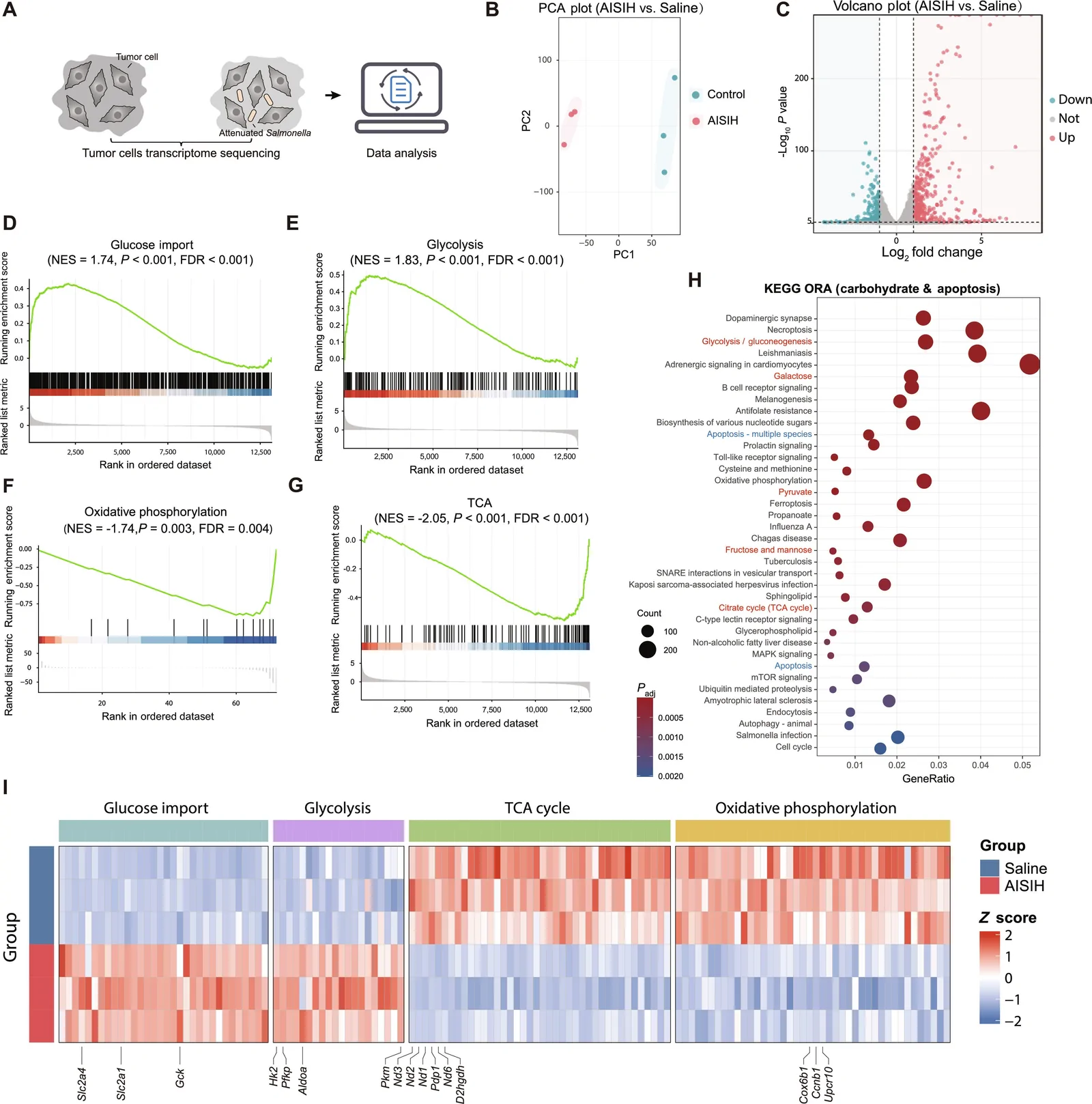
Antitumor bacteria induce metabolic reprogramming in bladder cancer cells. (A) MB49 mouse bladder cancer cells were cocultured with attenuated *Salmonella* AISIH (MOI = 100) for 6 h. Following coculture, the MB49 cells were harvested for RNA-seq, whereas MB49 cells treated with physiological saline under identical conditions were used as the control. (B and C) PCA (B) and volcano plot (C) of the RNA-seq data (AISIH versus Saline) from MB49 tumor cells collected from the experimental model shown in (A). (D to G) GSEA using curated gene sets from MSigDB revealed that genes associated with the glucose import signaling pathway (D) and glycolysis signaling pathway (E) were up-regulated, whereas those associated with the oxidative phosphorylation pathway (F) and TCA cycle pathway (G) were down-regulated in AISIH-treated cells compared with saline-treated control cells. (H) KEGG ORA revealed enrichment of pathways related to carbohydrate metabolism and apoptosis in MB49 cells treated with AISIH compared with saline-treated MB49 cells. The dot plot displays the enrichment results, where the GeneRatio indicates the degree of enrichment, and the Benjamini–Hochberg-adjusted *P* values (*P*_adj_) reflect the statistical significance of each pathway. Pathways related to apoptosis are highlighted in blue, whereas those associated with carbohydrate metabolism are highlighted in red. (I) Clustering heatmap of the expression of representative DEGs related to glucose import (*Slc2a4*, *Slc2a1*, *Gck*), glycolysis (*Hk2*, *Pfkp*, *Aldoa*, *Pkm*), TCA cycle (*Nd3*, *Nd2*, *Nd1*, *Pdp1*, *Nd6*, *D2hgdh*), and oxidative phosphorylation (*Cox6b1*, *Ccnb1*, *Uqcr10*) pathways in MB49 mouse bladder cancer cells following 6 h of coculture with AISIH or physiological saline treatment. Genes known to promote glucose import and glycolysis were generally up-regulated in bacteria-treated tumor cells, whereas genes involved in the TCA cycle and oxidative phosphorylation were generally down-regulated. *n* = 3 biologically independent cells per group. PCA, principal components analysis; TCA, tricarboxylic acid; KEGG, Kyoto Encyclopedia of Genes and Genomes; GSEA, gene set enrichment analysis; MOI, multiplicity of infection; NES, normalized enrichment score; FDR, false discovery rate; ORA, over-representation analysis; *Aldoa*, aldolase A; *D2hgdh*, D-2-hydroxyglutarate dehydrogenase; *Gck*, glucokinase; *Hk2*, hexokinase 2; *Nd1*, NADH dehydrogenase subunit 1; *Nd2*, NADH dehydrogenase subunit 2; *Nd3*, NADH dehydrogenase subunit 3; *Nd6*, NADH dehydrogenase subunit 6; *Pdp1*, pyruvate dehydrogenase phosphatase 1; *Pfkp*, phosphofructokinase, platelet; *Pkm*, pyruvate kinase M1/2; *Slc2a1*, solute carrier family 2 member 1; *Slc2a4*, solute carrier family 2 member 4; *Ccnb1*, cyclin B1; *Cox6b1*, cytochrome c oxidase subunit 6B1; *Uqcr10*, ubiquinol-cytochrome c reductase complex III subunit X.

### *Salmonella* can directly compete with tumor cells for glucose

We hypothesized that the abnormal glucose metabolism observed in tumor cells upon infection with the attenuated antitumor *Salmonella* strain was due to the direct modulation of glucose uptake in tumor cells by the bacterium (Fig. 2A). This hypothesis was preliminarily supported by the observation that heat-inactivated AISIH caused less pronounced alterations in glucose metabolism-related gene expression in tumor cells than live AISIH. In addition, we observed that the expression of genes associated with oxidative phosphorylation was significantly down-regulated following bacterial infection, whereas heat-inactivated AISIH did not induce significant down-regulation of these genes. These findings suggest that bacterial infection promotes a metabolic shift in tumor cells toward glycolysis for glucose utilization (Fig. 2B). Next, the AISIH strain was cocultured with the mouse bladder cancer cell line MB49 in vitro to assess its ability to invade tumor cells and survive intracellularly. Gentamicin was used to eliminate extracellular bacteria without affecting intracellular bacterial viability [31]. The results demonstrated that the AISIH strain efficiently infiltrated tumor cells; after 4 h of coculture, a mean of approximately 461 intracellular bacteria per 100 tumor cells was detected. Fluorescence imaging provided direct visualization of infiltrated AISIH bacteria within tumor cells (Fig. S4A and B). These bacteria survived for more than 20 h and continued to proliferate (Fig. S4C and D), which is consistent with the previous reports that described the persistence of *Salmonella* within tumor cells [32]. We subsequently assessed glucose uptake in tumor cells after coculture by measuring the level of intracellular fluorescently labeled glucose (2-NBDG) (Fig. 2C). 2-NBDG is a fluorescent analog of 2-deoxyglucose in which the second oxygen atom is replaced by a fluorescent moiety. It enters cells through glucose transporters and is widely used to evaluate glucose uptake in cells [33]. Uninfected MB49 cells displayed strong green fluorescence, indicating effective 2-NBDG uptake. In contrast, compared with uninfected controls, MB49 cells infected with the AISIH strain presented a significantly reduced intracellular fluorescence intensity (approximately a 23.8% reduction) (Fig. 2D and E), which suggested a diminished glucose uptake capacity.

**Fig. 2.**
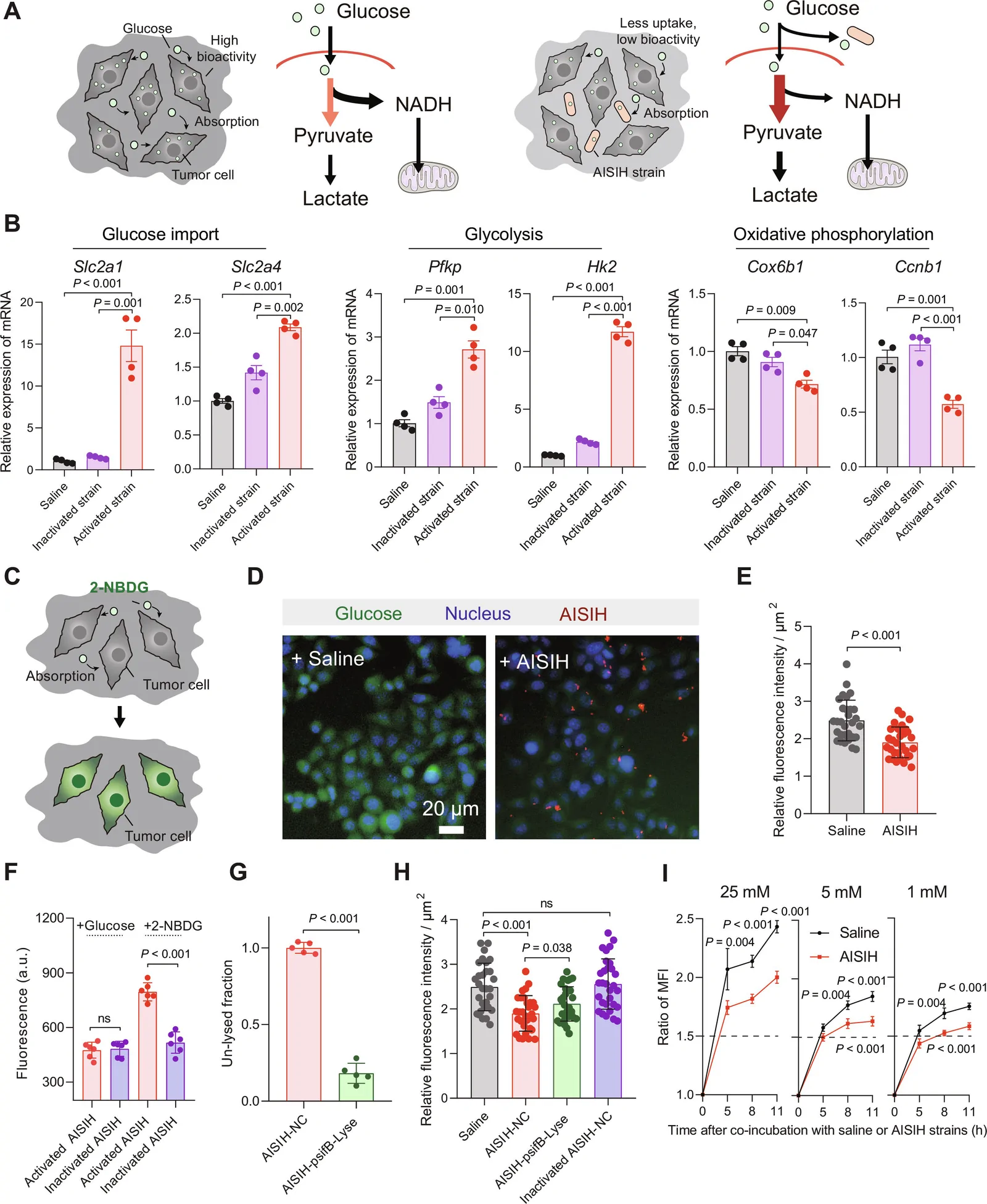
Attenuated *Salmonella* can directly compete with tumor cells for glucose nutrition. (A) Schematic diagram of hypothesized attenuated *Salmonella*-induced alterations in tumor cell glucose metabolism. Under normal conditions, tumor cells absorb glucose, which subsequently undergoes intracellular glycolysis and oxidative phosphorylation. When attenuated *Salmonella* AISIH strain coexist with tumor cells, they consume the limited amounts of glucose, reducing glucose availability for tumor cells and triggering a metabolic shift toward faster glycolysis. The thicker red arrow indicates increased glycolytic flux and pyruvate production in tumor cells under glucose competition with attenuated *Salmonella*. (B) Changes in the expression of representative genes related to cellular glucose metabolism and oxidative phosphorylation pathways after 6 h of treatment with saline, heat-inactivated AISIH strain, or viable (activated) AISIH bacteria. These genes include the glucose uptake genes *Slc2a1* and *Slc2a4*, the glycolysis genes *Pfkp* and *Hk2*, and the oxidative phosphorylation genes *Cox6b1* and *Ccnb1*. Inactivation of the bacteria significantly alleviated the changes in the levels of these genes (*n* = 4). (C) Schematic of 2-NBDG uptake by tumor cells. Cellular uptake of 2-NBDG was detected by measuring green fluorescence intensity. (D) Representative fluorescence images of tumor cells cocultured with the AISIH strain in 2-NBDG-supplemented medium for 4 h (MOI of 100). An engineered strain expressing spontaneous red fluorescence was used. Scale bar, 20 μm. (E) Comparative data from fluorescence quantification of cytoplasmic regions in individual tumor cells, which were randomly selected from 3 to 5 fields in (D) (*n* = 30). (F) Determination of relative fluorescence intensity by a microplate assay in AISIH and paraformaldehyde-inactivated AISIH strains incubated with either unlabeled glucose (negative control for background fluorescence) or the fluorescent glucose analog 2-NBDG. Increased fluorescence in the 2-NBDG-treated groups reflects uptake of the fluorescent glucose analog. a.u. denotes arbitrary units (*n* = 6). (G) Plate count analysis was used to determine the survival of the AISIH and AISIH-psifB-Lyse strain within tumor cells. Unlike the blank control group, most intracellular *Salmonella* bacteria with psifB-Lyse were lysed (*n* = 5). (H) Quantitative analysis of tumor cell 2-NBDG uptake following different treatments. Tumor cells were cocultured with saline, AISIH strain, AISIH-psifB-Lyse strain, and the paraformaldehyde-inactivated AISIH strain for 6 h. The fluorescence intensity of 2-NBDG within tumor cells was quantified as an indicator of glucose uptake. Three to 5 fields of view were randomly selected for fluorescence quantification of cytoplasmic regions in individual tumor cells (*n* = 30). Active strains effectively reduced 2-NBDG uptake in tumor cells. The AISIH-psifB-Lyse strain also reduced 2-NBDG uptake, although to a lesser extent. The inactivated AISIH strain failed to reduce 2-NBDG uptake in tumor cells. (I) Proliferation of MB49 tumor cells after incubation with saline or AISIH strains in the presence of 1, 5, or 25 mM glucose. The cell nuclei dye Hoechst was used to detect changes in the number of cells. The “Ratio of MFI” refers to the ratio of the MFI emitted by tumor cells that were stained with Hoechst at each time point relative to that measured at 0 h. The data are presented as the means ± SDs (B and E to I). All the data are representative of 2 independent experiments. ns, not significant. 2-NBDG, 2-[*N*-(7-nitrobenz-2-oxa-1,3-diazol-4-yl)amino]-2-deoxy-d-glucose; MOI, multiplicity of infection; NADH, nicotinamide adenine dinucleotide; MFI, mean fluorescence intensity.

We hypothesized that this reduction in tumor cell glucose uptake might be due to concurrent glucose consumption by both extracellular and intracellular bacteria. Indeed, the metabolically active extracellular bacteria displayed robust glucose uptake, whereas the paraformaldehyde-inactivated bacteria exhibited markedly diminished glucose uptake (Fig. 2F). AISIH-psifB-Lyse is an engineered strain harboring a plasmid in which *Lyse* expression is regulated by the *sifB* promoter, enabling intracellular activation and spontaneous bacterial lysis (Fig. S5A and B) [32,34]. The growth rate and glucose uptake capacity of AISIH-psifB-Lyse in culture medium did not differ significantly from those of the control AISIH strain (Fig. S5C and D). However, AISIH-psifB-Lyse had dramatically reduced intracellular survival (>80% bacterial death) (Fig. 2G). We next measured glucose uptake after coincubation of AISIH-psifB-Lyse with tumor cells. Similar to the AISIH strain, AISIH-psifB-Lyse also competed with tumor cells for a limited supply of glucose, leading to a reduction in glucose uptake by tumor cells compared with that in the saline-treated group (Fig. 2H). The AISIH-psifB-Lyse strain had lost most of its intracellular activity and thus the ability to compete for glucose intracellularly. These findings indicate that bacteria can compete with tumor cells for glucose both intracellularly and extracellularly. Notably, the fluorescence intensity of tumor cells treated with AISIH-psifB-Lyse was 11.3% greater than that of AISIH-treated cells (Fig. 2H). Conversely, paraformaldehyde-inactivated AISIH failed to suppress glucose uptake in tumor cells, suggesting that this competitive effect occurs primarily in the extracellular compartment and depends on bacterial metabolic activity (Fig. 2H). The proliferation of tumor cells is directly limited by extracellular glucose availability [35]. Consistently, indirect coculture of bacterial strains with tumor cells, which was performed by using a Transwell system, led to pronounced inhibition of tumor cell growth across different extracellular glucose concentrations (Fig. 2I and Fig. S5E). This mechanism may partly explain how bacteria can achieve substantial tumor growth suppression under relatively low-glucose conditions, which are typical for the tumor microenvironment [36]. In summary, our findings demonstrate that *Salmonella* effectively competes with tumor cells for glucose, thereby indirectly inhibiting tumor cell proliferation.

To assess whether this competition for glucose markedly affects immune cells, primary immune cells isolated from mouse spleens were cultured under different glucose conditions. Compared with tumor cells, immune cells are less sensitive to glucose deprivation. Specifically, immune cell viability was 20.16% greater than that of tumor cells at 6 h. With prolonged culture, immune cell viability remained at 65.7% at 24 h, whereas tumor cell viability decreased to only 12.3% (Fig. S6), indicating that immune cells are pronouncedly less sensitive to glucose deprivation than tumor cells are. This can be explained by a greater metabolic plasticity of immune cells and their ability to switch to alternative energy sources and metabolic pathways to sustain their function [37].

### Attenuated *Salmonella* with an enhanced glucose absorption ability has improved anticancer effects

We confirmed that a single administration of the AISIH strain led to direct competition of bacteria with tumor cells for glucose in vitro, thereby modulating glucose metabolism at both the individual tumor cell and whole-tumor levels. Importantly, as the environmental glucose concentration decreased, the inhibitory effect of bacterial competition on tumor cells became increasingly pronounced (Fig. 2). Typically, bacteria involve abundant intracellular enzymes to absorb nutrients such as glucose from their surroundings to sustain their metabolic processes [38,39]. We hypothesized that genetic modification of the gluconeogenesis regulatory pathway in the AISIH strain could increase its glucose uptake and absorption capacities, thereby enabling more potent nutritional competition with tumor cells and ultimately yielding more effective tumor therapy (Fig. 3A). Accordingly, we sequentially knocked out major genes, including *yidA* [catalyzing the dephosphorylation of glucose 6-phosphate (Glc-6-P)], *yigL* (also dephosphorylating Glc-6-P), *glpX* (converting fructose 1,6-bisphosphate to fructose 6-phosphate), and *ppsA* (catalyzing the reverse conversion from pyruvate to phosphoenolpyruvate) [38,39], that are involved in gluconeogenesis in *Salmonella* to generate the chassis strains Δ*yidA*-AISIH, Δ*yigL*-AISIH, Δ*glpX*-AISIH, and Δ*ppsA*-AISIH (Fig. 3B and C). The colony morphologies of these mutants did not pronouncedly differ from those of the parental AISIH strain. However, the growth rates of the Δ*yidA*-AISIH and Δ*yigL*-AISIH strains were markedly lower than those of the other strains (Fig. 3D and E). This may result from elevated levels of intracellular sugar phosphate intermediates, which inhibit carbon metabolic pathways and trigger stress responses that suppress growth [39]. We then evaluated glucose uptake by the mutant strains. It has been reported that blocking bacterial gluconeogenesis triggers a compensatory increase in the glucose uptake capacity [40]. Consistently, glucose uptake by the Δ*yidA*-AISIH, Δ*yigL*-AISIH, Δ*glpX*-AISIH, and Δ*ppsA*-AISIH strains increased 1.48-, 1.47-, 1.20-, and 1.15-fold, respectively, relative to that by the WT AISIH strain (measured at the 2-NBDG-specific absorption wavelength of 542 nm). Among these strains, the Δ*yidA*-AISIH and Δ*yigL*-AISIH strains showed the largest increase in glucose uptake (Fig. 3F). Next, we assessed the therapeutic efficacy of these strains in a subcutaneous bladder cancer syngeneic model. Compared with the saline-treated group, a single intraperitoneal dose of AISIH produced substantial antitumor activity in tumor-bearing mice (Fig. 3G and H), which is consistent with the findings of earlier reports [5]. Among the 4 modified strains, the Δ*yidA*-AISIH and Δ*yigL*-AISIH strains showed no significant difference in antitumor activity compared with the WT AISIH strain. The Δ*ppsA*-AISIH strain exhibited moderately enhanced antitumor activity relative to the above 3 strains, whereas the Δ*glpX*-AISIH strain (subsequently designated GLA1) demonstrated significantly enhanced antitumor activity, which was in line with its sufficient glucose uptake capacity and stable growth performance (Fig. 3G and H and Fig. S7A and B).

**Fig. 3.**
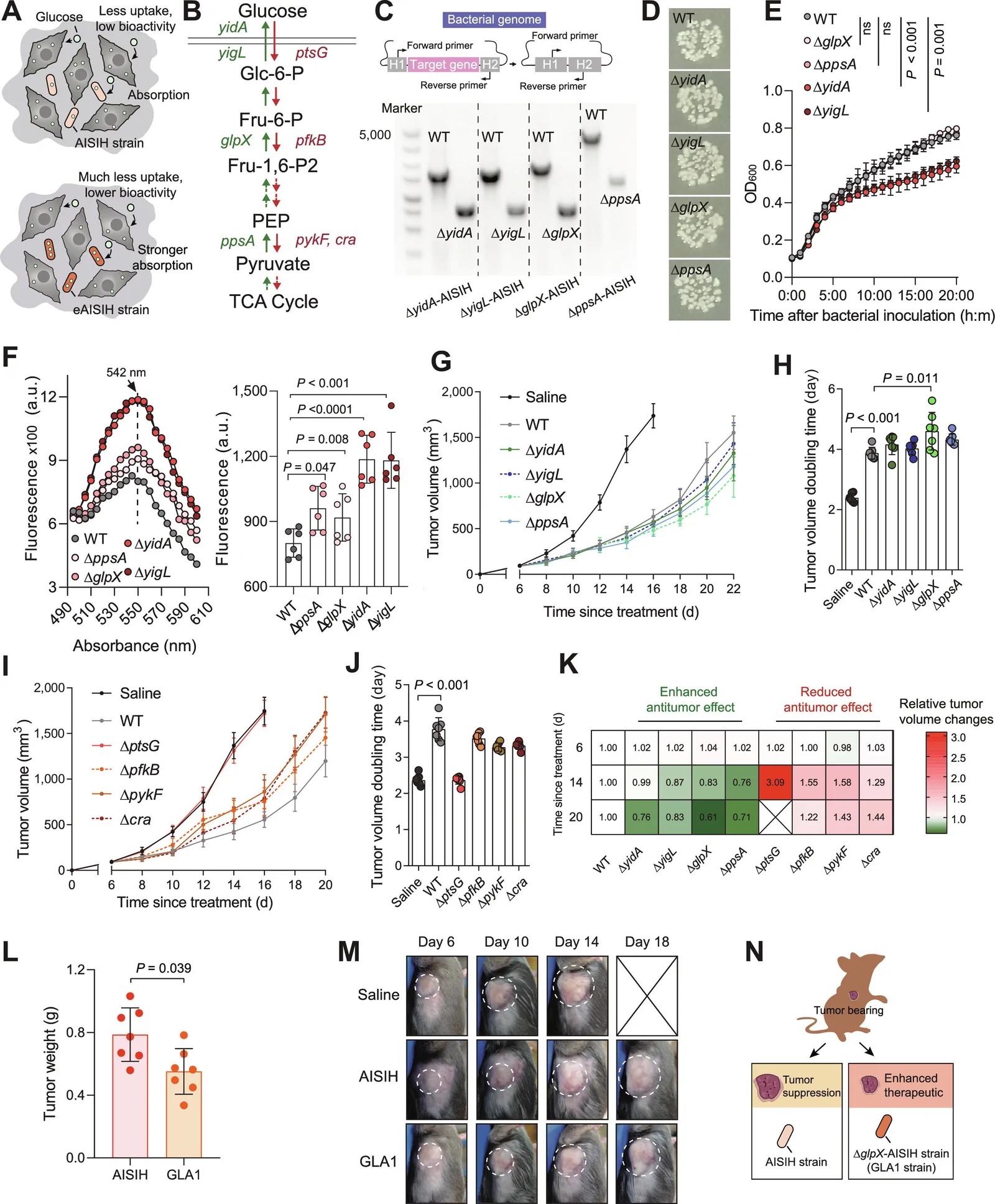
Glucose uptake and antitumor activity are increased in the *glpX*-deficient attenuated *Salmonella* strain. (A) Schematic of hypothesized competitive glucose uptake between attenuated *Salmonella* and tumor cells. WT attenuated *Salmonella* (AISIH) directly competes with tumor cells for glucose uptake in the environment. An improved genetically modified strain (eAISIH) should achieve more efficient competitive glucose uptake against tumor cells. (B) Schematic of key genes involved in glycolysis (including *ptsG*, *pfkB*, *pykF*, and *cra*) and gluconeogenesis (including *yidA*, *yigL*, *glpX*, and *ppsA*) in bacteria. (C) Schematic diagram of the CRISPR-modified attenuated *Salmonella* genome (top) and agar plate verification confirming the successful construction of 4 novel monogenic gluconeogenesis-deficient attenuated *Salmonella* strains (Δ*yidA*-AISIH, Δ*yigL*-AISIH, Δ*glpX*-AISIH, and Δ*ppsA*-AISIH). The WT unmodified AISIH strain served as the control. (D) Comparison of plate colony morphologies among the 5 attenuated *Salmonella* strains in (C). (E) Comparison of growth curves in LB medium for the 5 attenuated *Salmonella* strains in (C). With the exception of the *yidA* and *yigL* gene-deficient strains, the remaining strains presented similar growth rates (*n* = 3). (F) The glucose uptake capacity of AISIH and its gluconeogenic mutant strains, which were incubated with 2-NBDG for 30 min, was measured at different emission wavelengths with a fluorometer (left), and comparison of fluorescence intensity among the strains at the peak emission wavelength (542 nm) (right) (excitation wavelength: 488 nm). Compared with the WT strain, all 4 mutant strains presented significantly greater glucose uptake (*n* = 6). (G) Evaluation of the efficacy of AISIH and its gluconeogenesis gene mutant strains in the treatment of a subcutaneously transplanted tumor model of MB49 bladder cancer in mice. MB49 tumor-bearing mice were treated with intraperitoneal injection of AISIH, ∆*glpX*-AISIH, ∆*pps*-AISIH, ∆*yidA*-AISIH, or ∆*yigL*-AISIH at a dose of 1 × 10^6^ CFU per mouse (*n* = 7). Among these mutants, ∆*glpX*-AISIH showed the strongest antitumor efficacy, followed by ∆*ppsA*-AISIH, ∆*yidA*-AISIH, and ∆*yigL*-AISIH. (H) Comparison of tumor doubling times after treatment with AISIH and its gluconeogenesis-related gene mutant strain in the tumor-bearing mice in (G). (I) Evaluation of the therapeutic efficacy of WT AISIH and its glycolysis-related gene mutant strains in a subcutaneous MB49 bladder cancer model. MB49 tumor-bearing mice were treated with intraperitoneal injection of AISIH or the indicated glycolysis-related gene mutant strains at a dose of 1 × 10^6^ CFU per mouse (*n* = 7). Compared with the WT AISIH strain, all glycolysis-related gene mutant strains exhibited reduced antitumor efficacy. Among the tested strains, WT AISIH showed the strongest antitumor effect, followed by ∆*pfkB*-AISIH, ∆*pykF*-AISIH, ∆*cra*-AISIH, and ∆*ptsG*-AISIH. (J) Comparison of tumor doubling times after treatment with AISIH and its glycolysis-related gene mutant strain in the tumor-bearing mice in (H). (K) Relative tumor volume changes at different time points (6, 14, and 20 d) after treatment of tumor-bearing mice with WT AISIH, its gluconeogenesis-deficient mutant strain, and its glycolysis-deficient mutant strain. Compared with the WT AISIH strain, the gluconeogenesis-deficient mutant strain presented enhanced anticancer effects, whereas the glycolysis-deficient mutant strain presented reduced anticancer effects. All the mice infected with the *ptsG*-deficient AISIH strain died by day 18. The color scale indicates relative antitumor efficacy: Green denotes enhanced antitumor effects, and red denotes reduced antitumor effects. Increasing color intensity reflects a greater magnitude of enhancement (green) or reduction (red) in antitumor efficacy. (L) Tumor weights were measured at day 20 after treatment in mice receiving WT AISIH or the *glpX*-deficient strain (GLA1) (*n* = 7). (M) Representative tumor images of MB49 subcutaneous tumor-bearing mice at 6, 10, 14, and 18 d after treatment with the AISIH and GLA1 strains. The tumor locations are indicated by white dashed circles. The saline group served as a control. “×” denotes mouse death. (N) Schematic comparison of the anticancer effects of the AISIH and GLA1 strains. The data are presented as the means ± SDs. All the data are representative of 2 independent experiments. ns, not significant; WT, wild-type; GLA1, *glpX*-deficient strain; 2-NBDG, 2-[*N*-(7-nitrobenz-2-oxa-1,3-diazol-4-yl)amino]-2-deoxy-d-glucose; *ptsG*, phosphotransferase system glucose-specific transporter component; *pfkB*, phosphofructokinase B; *pykF*, pyruvate kinase F; *cra*, catabolite repressor/activator; *yidA*, putative phosphatase YidA; *yigL*, haloacid dehalogenase-like hydrolase YigL; *glpX*, fructose-1,6-bisphosphatase II; *ppsA*, phosphoenolpyruvate synthase.

To further validate the role of the bacterial glucose uptake capacity in anticancer efficacy, we also targeted key glycolytic genes in *Salmonella*, including *ptsG* (mediating efficient glucose transport and phosphorylation to Glc-6-P), *pfkB* (catalyzing the conversion of fructose 6-phosphate to fructose 1,6-bisphosphate), *pykF* (catalyzing the conversion of phosphoenolpyruvate to pyruvate), and *cra* (a principal global transcription regulator of carbohydrate metabolism), to construct the Δ*ptsG*-AISIH, Δ*pfkB*-AISIH, Δ*pykF*-AISIH, and Δ*cra*-AISIH strains (Fig. 3B) [38,39]. In vitro growth curve analysis revealed no significant differences in the growth rates between these defective strains and the WT AISIH strain, but all the mutant strains presented reduced glucose uptake capacity to varying degrees (Fig. S7C and D). Subsequent therapy experiments using the same bladder cancer model revealed that deletion of the glucose metabolism-related genes (*cra, ptsG, pfkB, and pykF*) impaired the antitumor efficacy of the corresponding strains to varying degrees. Among these mutants, the Δ*ptsG*-AISIH strain exhibited the most pronounced defect, displaying virtually no anticancer activity (Fig. 3I to K and Fig. S7E and F). Heatmap visualization of relative changes in the tumor volume (Fig. 3K) provided a clearer comparison of antitumor efficacy across the various strains than did the tumor doubling time data alone. Compared with the parental AISIH strain, the gluconeogenesis-deficient strains with increased glucose uptake (Δ*yidA*-AISIH, Δ*yigL*-AISIH, Δ*glpX*-AISIH, and Δ*ppsA*-AISIH) increased tumor doubling times by 7.39%, 4.08%, 18.83%, and 11.65%, respectively. In contrast, the glycolytic gene-deficient strains with reduced glucose uptake (Δ*ptsG*-AISIH, Δ*pfkB*-AISIH, Δ*pykF*-AISIH, and Δ*cra*-AISIH) shortened tumor doubling times by 37.29%, 6.84%, 13.19%, and 12.04%, respectively (Fig. 3H, J, and K and Fig. S7B and E). Collectively, these findings indicate that increasing the glucose uptake capacity of antitumor *Salmonella* strains is accompanied by enhanced anticancer efficacy. Compared with the original chassis strain, the Δ*glpX*-AISIH strain (GLA1) exhibited significantly greater anticancer efficacy. The tumor weight on day 20 after treatment with GLA1 was 70.2% lower than that in the AISIH-treated group (Fig. 3L to N). The GLA1 strain is therefore a superior novel attenuated *Salmonella* chassis, and it was used in subsequent experiments.

### The GLA1 strain can markedly activate the STING pathway in tumor cells by inducing their starvation

Under nutrient-poor conditions, tumor cells often exhibit dysfunction of antioxidant systems, such as the glutathione system, rendering them unable to clear ROS effectively [41]. In our experiments, compared with those cultured under high-glucose conditions, tumor cells treated with saline and cultured under moderate- and low-glucose conditions presented elevated intracellular ROS levels (~1.12- and 1.19-fold increases, respectively; Fig. 4A and B). Notably, compared with the saline-treated group, coincubation of attenuated *Salmonella* with tumor cells increased intracellular ROS levels. Relative to the AISIH strain, the GLA1 strain induced even greater increases, with ROS levels being 1.07-, 1.15-, and 1.18-fold higher under high-, medium-, and low-glucose conditions, respectively (Fig. 4A to C). These findings confirm that bacteria-induced glucose deprivation promotes the tumor cell starvation state, which is accompanied by elevated ROS levels. Given that excessive ROS may cause mitochondrial damage, we next assessed mitochondrial integrity in tumor cells that were subjected to glucose limitation and bacterial competition. Compared with the saline-treated group, MMP assays revealed that both bacterial strains induced significant mitochondrial membrane depolarization, with GLA1 exerting a more pronounced effect than AISIH (Fig. 4D). TEM provided further direct visualization of pronounced mitochondrial structural abnormalities in MB49 tumor cells exposed to GLA1 compared with those exposed to the control strain (Fig. 4E). Together, these data suggest that the GLA1 strain induces substantial mitochondrial stress in tumor cells. mtDNA damage is a hallmark of mitochondrial stress, and mtDNA may be released into the cytoplasm via the mitochondrial permeability transition pore [42]. We therefore quantified cytoplasmic mtDNA levels in MB49 cells across different treatment groups via qPCR. The results indicated that compared with the control or AISIH groups, the GLA1 group presented higher cytoplasmic mtDNA levels, which were consistent with the increased ROS production in this group (Fig. 4F). Consistent with the elevated ROS levels and resulting mitochondrial damage [41], significantly greater levels of apoptosis were detected in the GLA1 group than in the AISIH group of cocultured MB49 cells (MOI of 100, 6 h) at various glucose concentrations (Fig. S8A and B). Under low-glucose conditions, the AISIH strain induced nearly 20% tumor cell apoptosis, whereas GLA1 induced >25% apoptosis; the saline control group displayed <5% apoptosis. In the absence of bacterial strains, the levels of tumor cell apoptosis did not significantly vary with respect to glucose concentrations (Fig. S8A and B). Furthermore, tumor cells exhibited greater sensitivity to bacterially triggered apoptosis as the glucose concentration decreased, indicating that glucose-deprived tumor cells are more responsive to extrinsic proapoptotic stimuli such as bacterial infection.

**Fig. 4.**
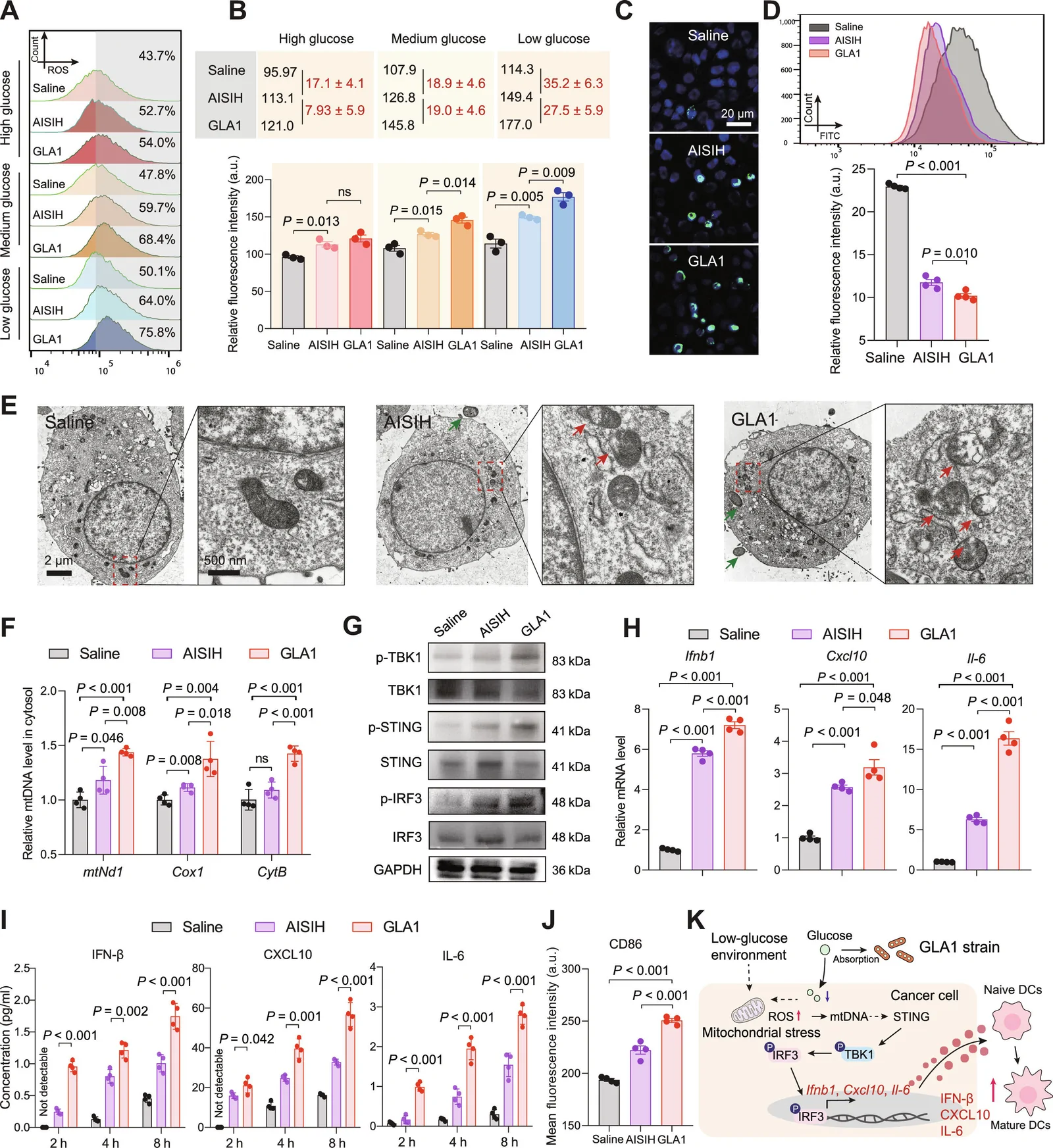
The GLA1 strain induces STING pathway activation in tumor cells, which is accompanied by increased levels of IFN-I, and promotes DC maturation. (A) Intracellular ROS production was determined after 3 h of coculture of MB49 tumor cells with various attenuated *Salmonella* strains, including AISIH and GLA1, at low (1 mM), medium (5 mM), and high (25 mM) glucose concentrations (MOI of 100). Representative FCM plots are shown. (B) Quantification of cellular ROS levels under different conditions from (A). The data are presented as the MFI per group, with red numbers indicating the difference between groups ± SD (top). Bar chart of the statistical analysis (bottom) (*n* = 3). The ROS levels induced by *Salmonella* in tumor cells progressively increased with decreasing glucose concentrations in the medium. (C to H) Attenuated *Salmonella* and MB49 tumor cells were cocultured at an MOI of 100 for 3 h under low-glucose conditions, followed by tumor cell analysis. Saline-treated cells served as blank controls. (C) Representative fluorescence images of ROS detection in tumor cells under low-glucose conditions. Compared with the AISIH strain, the GLA1 strain induced significantly greater ROS production in tumor cells. Scale bar, 20 μm. (D) Changes in the MMP of tumor cells induced by different strains under low-glucose conditions. The GLA1 strain induced significant changes in the MMP (*n* = 4). (E) Representative TEM images of mitochondrial changes in tumor cells induced by different strains under low-glucose conditions. The mitochondrial images from each group are magnified for display. The green arrows indicate the AISIH and GLA1 strains, while the red arrows indicate the swollen mitochondrial structures within the tumor cells. Compared with saline-treated cells, GLA1-treated cells exhibited significant mitochondrial damage. Scale bars, 2 μm or 500 nm. (F) Representative results of detection of free mtDNA (including *mtNd1*, *Cox1*, and *CytB*) in the cytoplasm after different treatments (*n* = 4). (G) Western blot analysis of STING pathway-related protein levels in tumor cells after different treatments. The GLA1 strain significantly activated the STING pathway in tumor cells. (H) Real-time quantitative PCR detection of the expression of representative genes, including *Ifnb1*, *Cxcl10*, and *Il-6* (*n* = 4), upon STING pathway activation. (I) ELISA detection of STING pathway-related cytokine levels in the supernatants of tumor cells subjected to different treatments (MOI of 100; 5 × 10^5^ tumor cells in 1.0 ml of culture medium) under low-glucose conditions. The supernatants were analyzed at 2, 4, and 8 h after bacterial treatment of tumor cells. The GLA1 strain effectively promoted the production of related cytokines (*n* = 4). (J) Assessment of DC maturation (CD86^+^ BMDCs) induced by tumor cell supernatants. Tumor cells were seeded at a density of 4 × 10^6^ cells/ml and cocultured with *Salmonella* strains (MOI of 100) for 8 h; the mixed supernatant was used to culture 2 × 10^5^ DCs for 24 h. The differences in the mean fluorescence intensity of CD86 on the cells were compared (*n* = 4). DC maturation was significantly induced by the supernatants of GLA1-treated tumor cells. (K) Schematic illustration of GLA1-induced STING pathway activation in tumor cells, which is accompanied by increased levels of IFN-I and enhanced DC maturation. Through competition for glucose, the GLA1 strain induces tumor cells to enter a starvation state, which is accompanied by mitochondrial stress, elevated ROS levels, subsequent mitochondrial damage, and the release of mtDNA into the cytoplasm, resulting in the activation of the cellular STING pathway. Increased secretion of proinflammatory factors, such as IFN-β, CXCL10, and IL-6, by activated tumor cells leads to the induction of DC maturation. The data are presented as the means ± SDs. All the data are representative of 2 independent experiments. ns, not significant; MFI, mean fluorescence intensity; GLA1, *glpX*-deficient strain; mtDNA, mitochondrial DNA; ROS, reactive oxygen species; BMDCs, bone marrow-derived dendritic cells; 2-NBDG, 2-[*N*-(7-nitrobenz-2-oxa-1,3-diazol-4-yl)amino]-2-deoxy-d-glucose; ELISA, enzyme-linked immunosorbent assay; DC, dendritic cells; IFN-β, interferon-β; CXCL10, C-X-C motif chemokine ligand 10; IL-6, interleukin-6; TEM, transmission electron microscopy; MOI, multiplicity of infection; STING, stimulator of interferon genes; TBK1, tank-binding kinase 1; IRF3, interferon regulatory factor 3; *mtNd1*, mitochondrially encoded NADH dehydrogenase 1; *Cox1*, cytochrome c oxidase subunit 1; *CytB*, cytochrome b; GLA1, *glpX*-deficient AISIH strain (Δ*glpX*-AISIH).

The cyclic GMP-AMP synthase (cGAS)/stimulator of interferon genes (STING) signaling can be an indicator of mtDNA damage and the subsequent release of fragmented DNA, as these processes result in the stimulation of cyclic GMP-AMP (cGAMP) synthesis and effective activation of the STING signaling cascade [43]. Accordingly, we performed Western blotting analysis to assess the phosphorylation levels of key proteins, namely, IRF3, TBK1, and STING, in the cGAS/STING axis. As shown in Fig. 4G and Fig. S8C, the phosphorylation levels of these proteins significantly increased in both the AISIH- and GLA1-treated groups, further confirming that the increase in ROS levels, which is accompanied by mtDNA release, activated the cGAS/STING pathway. Notably, the GLA1 strain caused more pronounced increases, indicating stronger activation of the cGAS/STING pathway in tumor cells. To further evaluate STING pathway activation in tumor cells, we examined changes in the expression of 3 STING-associated genes, namely, *Ifnb1* (a characteristic cytokine in the cGAS/STING pathway), *Cxcl10* (a key chemokine involved in STING signaling and T cell recruitment), and *Il-6* (a major proinflammatory cytokine induced by STING activation), in response to treatment. Compared with saline-treated cells, bacteria-treated cells presented significant up-regulation of all 3 genes, and the increases in the GLA1 strain-treated group were significantly greater than those in the AISIH strain-treated group (Fig. 4H). Under low-glucose conditions, we subsequently measured the levels of IFN-β, CXCL10, and IL-6 in the tumor cell supernatants at different time points after treatment. Consistent with the qPCR results, the levels of STING pathway-dependent type I IFN-β and its downstream cytokines (CXCL10 and IL-6) increased rapidly and significantly in both bacterial strain groups, with the greatest increases in the GLA1 group; at 2 h after treatment, the cytokine levels in the supernatants of the GLA1-treated cells increased 3.87-fold (IFN-β), 1.32-fold (CXCL10), and 5.93-fold (IL-6) compared with those in the AISIH group (Fig. 4I). These findings further demonstrated stronger activation of the cGAS/STING pathway in tumor cells treated with the GLA1 strain. Given the pivotal role of IFN-β, even at low levels, in innate immune activation [44], we assessed the maturation of BMDCs following coculture with tumor cell supernatants. Because the number of tumor cells far exceeds that of DCs in the tumor microenvironment, we increased the number of tumor cells used for supernatant preparation. The final concentration of IFN-β in the mixed supernatant from the GLA1 strain group was measured to be over 25 pg/ml (Fig. S8D). FCM analysis revealed a significant increase in the number of CD11c^+^CD80^+^CD86^+^ cells following AISIH strain treatment compared with that in the control group, with an even more pronounced increase in the GLA1 strain group (Fig. 4J and Fig. S8E and F). This enhanced BMDC maturation was attributed to the triggering of IFN-β production by cGAS/STING pathway activation in tumor cells. Collectively, these findings indicate that the GLA1 strain not only is a pronounced inducer of apoptosis in tumor cells but also activates the cGAS/STING pathway by inducing ROS accumulation and mtDNA release in tumor cells, thereby triggering innate immune responses (Fig. 4K).

### The engineered GLA2 strain achieves tumor vascular normalization through anti-TNF-α nanoantibody expression to enhance anticancer efficacy

Under hypoxic or nutrient-deprived conditions, tumors increase their nutrient supply by inducing aberrant intratumoral angiogenesis—a major factor limiting the efficacy of conventional starvation-based cancer therapies [45]. Herein, we demonstrated that attenuated *Salmonella* could compete with tumor cells for glucose, thereby inducing their metabolic starvation, which was accompanied by robust activation of the STING signaling pathway (Fig. 4). Transcriptomic profiling revealed that *Salmonella* infection caused profound transcriptional reprogramming in tumor cells. Analysis of the transcriptomic data identified the TNF signaling pathway as the most significantly altered pathway under *Salmonella* infection (Figs. S3A and S9A). This finding is notable, as elevated TNF-α levels are key initiators and amplifiers of the abnormal tumor vasculature [46,47]. Antibody-mediated blockade of TNF-α, combined with other treatments, can significantly increase tumor suppression [48,49]. Indeed, bacterium-induced TNF-α up-regulation has been linked to vascular abnormalities [50], and the clearance of TNF-α from the body may enhance the anticancer effects of bacteria [51]. Coculture of AISIH with MB49 cells resulted in marked up-regulation of pro-angiogenic genes (e.g., *Vegfa* and *Tnf*), marked down-regulation of anti-angiogenic genes (e.g., *Thbs1*), and extensive remodeling of angiogenesis-associated signaling pathways. The expression shift was calculated as the log_2_FC between the AISIH and saline groups, defined as log_2_(expression in AISIH/expression in saline). Positive values indicate higher expression in the AISIH group, whereas negative values indicate lower expression in the AISIH group (Fig. 5A and B and Fig. S9B). Elevated TNF-α levels were also detected in the supernatant of tumor cells that were cocultured with AISIH and GLA1, likely originating from STING pathway activation in tumor cells (Fig. S9C) [52]. To investigate whether *Salmonella* infection promotes abnormal tumor vascularization in vivo, we administered the GLA1 strain to bladder tumor-bearing mice and evaluated angiogenesis after 3 d. Tumors of comparable sizes were analyzed to control for volume-related confounders. Compared with the saline-treated control group, the GLA1-treated group exhibited pronounced intratumoral vascular proliferation (Fig. 5C). Fluorescent detection of the endothelial marker CD31 further confirmed the increased intratumoral vascular density in tumors infected with the GLA1 strain (Fig. 5D to F). Importantly, the intratumoral administration of a TNF-α-neutralizing antibody effectively suppressed GLA1-induced aberrant vascularization (Fig. S9D). These results suggest that combining bacterial anticancer therapy with TNF-α blockade may increase the metabolic starvation and STING pathway activation, which are induced by attenuated *Salmonella* through the inhibition of abnormal angiogenesis.

**Fig. 5.**
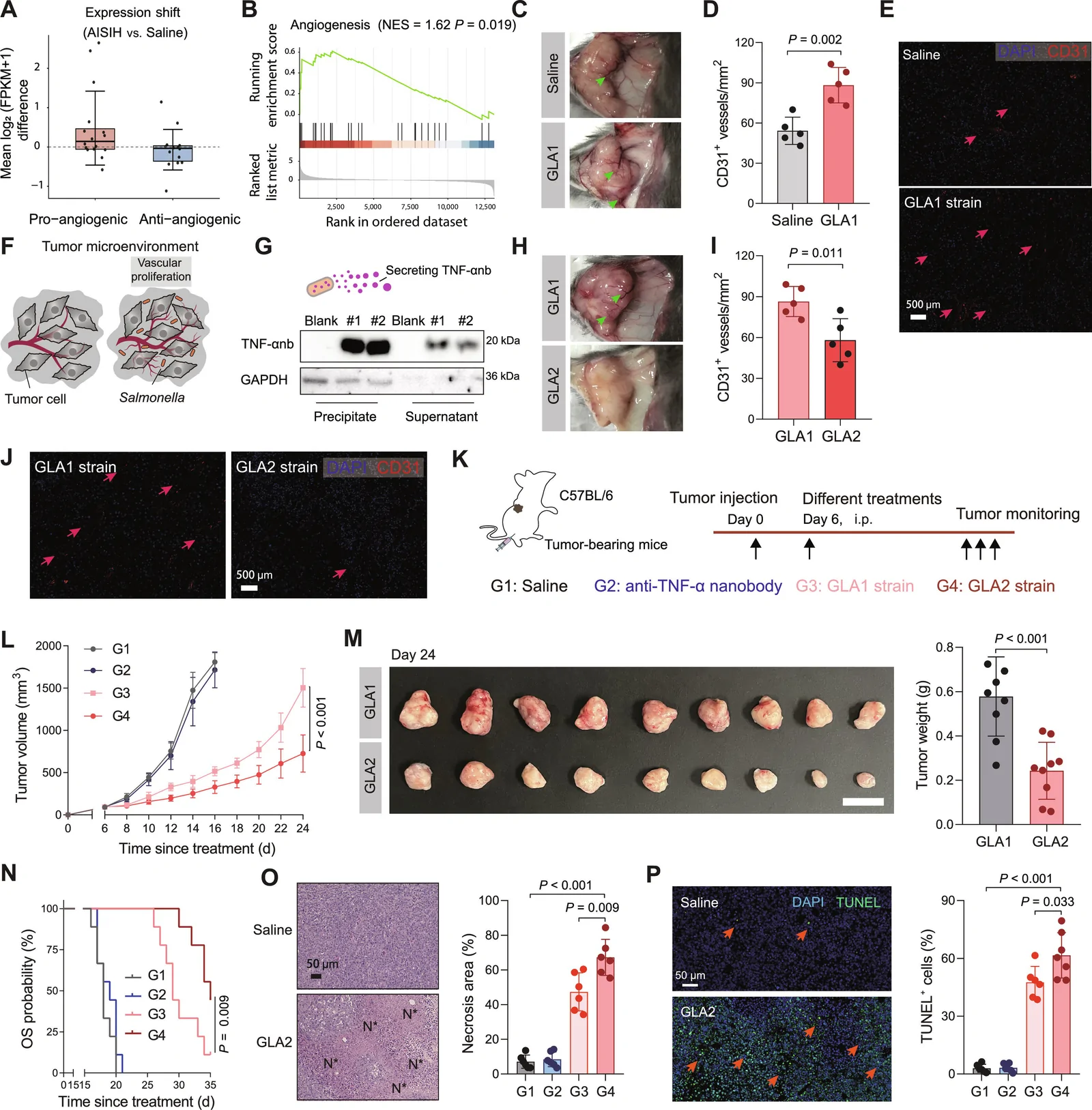
The GLA2 strain, which expresses an anti-TNF-α nanobody, achieves enhanced bladder cancer treatment efficacy. (A) Comparison of expression of pro-angiogenic and anti-angiogenic genes in MB49 tumor cells after coculture with AISIH or saline. Gene expression data were obtained from RNA-seq analysis of MB49 cells collected after 6 h of coculture with AISIH or saline treatment. (B) GSEA of an angiogenesis-related gene set from the MSigDB using RNA-seq data from MB49 cells cocultured with AISIH or saline for 6 h. (C to E) Comparison of differences in tumor vascularization on day 3 after the intraperitoneal administration of saline or the attenuated *Salmonella* GLA1 strain (3 × 10^6^ CFU/mouse). (C) Representative tumor dissection images are shown, with green arrows indicating tumor vessels. (D) The tumor vascular density was quantified as the number of vessel-like structures expressing CD31 (*n* = 5). (E) Tumor sections were immunofluorescently stained for the endothelial marker CD31; nuclei were counterstained with 4′,6-diamidino-2′-phenylindole (DAPI). Scale bars, 500 μm. The red arrows indicate blood vessels within the tumor. (F) Schematic of tumor vascular abnormalities induced by attenuated *Salmonella*. Compared with saline treatment, attenuated *Salmonella* treatment induces abnormal proliferation of tumor vessels. (G) Immunoblotting was used to detect TNF-αnb expression and secretion by the GLA2 strain. #1 and #2 represent 2 cherry-picked monoclones of the engineered strain. Total bacterial proteins are shown in the precipitate, and total proteins secreted into the extracellular space are shown in the supernatant. Engineered bacteria carrying a blank plasmid (blank) served as a control. (H to J) Comparison of differences in tumor vascularization 3 d after the intraperitoneal administration of the attenuated *Salmonella* GLA1 or GLA2 strain (3 × 10^6^ CFU/mouse). (H) Representative tumor dissection images are shown, with green arrows indicating tumor vessels. (I) The tumor vascular density was quantified as the number of vessel-like structures expressing CD31 (*n* = 5). (J) Tumor sections were immunofluorescently stained for the endothelial marker CD31; nuclei were counterstained with DAPI. Scale bars, 500 μm. The red arrows indicate blood vessels within the tumor. (K) Schematic of the therapeutic effects of different treatments (G1: saline, G2: anti-TNF-α nanoantibody, G3: GLA1 strain, and G4: GLA2 strain) on MB49 subcutaneous syngeneic tumor model mice. (L) Tumor growth profiles after different treatments in (K) (*n* = 9). (M) Photographs of tumors from mice in the GLA1 strain group and GLA2 strain group on day 24 (left) and comparison of tumor weights (right). Scale bar, 10 mm. (N) Survival curves of mice after tumor cell inoculation. The mice were euthanized upon reaching a humane endpoint. (O) Representative H&E-stained images of tumors from the saline and GLA2 treatment groups (left). Six randomly selected visual fields were analyzed, and the percentage of necrotic areas was quantified and presented as a bar chart (right). The label N* indicates a necrotic region. Scale bar, 50 μm. (P) Representative TUNEL images of tumors from the saline and GLA2 treatment groups (left). Six visual fields were randomly selected to determine the percentage of fluorescent cells (right). The orange arrows indicate the positive regions. Scale bar, 50 μm. The data are presented as the means ± SDs. All the data are representative of 2 independent experiments. ns, not significant; GSEA, gene set enrichment analysis; MSigDB, Molecular Signatures Database; DAPI, 4′,6-diamidino-2′-phenylindole; TNF-α, tumor necrosis factor-α; H&E, hematoxylin and eosin staining; TUNEL, terminal deoxynucleotidyl transferase-mediated deoxyuridine triphosphate nick end labeling; i.p., intraperitoneal injection; GLA1, *glpX*-deficient AISIH strain (Δ*glpX*-AISIH); GLA2, GLA1-derived strain expressing an anti-TNF-α nanobody under quorum-sensing control.

Next, we designed engineered strains for tumor-specific bacterial delivery of a TNF-α inhibitor—TNF-αnb—to achieve vascular normalization. Previous studies have shown that TNF-α blockade effectively suppresses aberrant vascular proliferation [53,54]. To ensure tumor-confined expression, the TNF-αnb sequence was fused downstream of the quorum-sensing promoter *pLuxI* (Fig. S10A). As bacterial populations expanded, the bioluminescence (LuxCDABE) signal increased in a density-dependent manner (Fig. S10B). In vivo imaging confirmed that quorum sensing-regulated bacteria exhibited negligible off-target expression, with protein production restricted to tumor tissue (Fig. S10C and D). We subsequently engineered a GLA1-derived strain (designated GLA2), which expressed TNF-αnb under quorum-sensing control (Fig. S10E and F). Immunoblotting confirmed the quorum sensing-mediated secretion of TNF-αnb into the supernatant via a pelB signal peptide (Fig. 5G). The GLA2 strain maintained comparable colony morphology and growth kinetics to those of GLA1 (Fig. S10G and H). Both the GLA1 and GLA2 strains were intraperitoneally administered to syngeneic bladder tumor-bearing mice. Three days posttreatment, histological analyses revealed that GLA2 markedly suppressed tumor vascular proliferation, which was accompanied by a significant reduction in the intratumoral CD31 level (Fig. 5H to J). Tumor growth curve analysis further demonstrated that compared with GLA1 treatment, GLA2 treatment resulted in superior tumor suppression (Fig. 5K and L). On day 24, the mean tumor weight in the GLA2-treated group was 0.2429 g, only 42.0% of that in the GLA1-treated group (0.5782 g) (Fig. 5M). Compared with saline treatment (18 d) or GLA1 treatment (29 d), GLA2 treatment also significantly prolonged the survival of tumor-bearing mice (to 35 d), resulting in 94.4% and 20.7% increases, respectively (Fig. 5N). Consistent with its enhanced antitumor efficacy, GLA2 induced more extensive tumor necrosis, as shown by H&E staining and TUNEL assays on day 3 (Fig. 5O and P). Notably, TNF-αnb monotherapy did not significantly inhibit tumor progression, whereas treatment with GLA2 resulted in greater tumor suppression than GLA1 alone, suggesting that TNF-αnb enhanced the antitumor efficacy of attenuated *Salmonella* (Fig. 5L). In summary, compared with GLA1, the engineered GLA2 strain achieves tumor vascular normalization through controlled, quorum sensing-regulated secretion of TNF-αnb, thereby increasing tumor necrosis and overall antitumor efficacy.

### The GLA2 strain exerts anticancer effects through intratumoral STING activation and immune remodeling

Given that attenuated *Salmonella* can directly activate the STING pathway in tumor cells, we examined the expression of cGAS/STING pathway-associated proteins and cytokines in tumors following bacterial treatment. Tumor tissues were collected 3 d post-administration, which corresponded to the near-peak bacterial load within tumors, and were analyzed by Western blotting. Compared with tumors in the saline control and AISIH-treated groups, tumors in the GLA1-treated group presented markedly increased levels of p-STING, p-TBK1, and p-IRF3, which was consistent with the increased STING pathway activation in cultured tumor cells. Notably, GLA2-treated tumors exhibited even stronger increases in the levels of these phosphorylated proteins. Compared with the GLA1-treated group, GLA2-treated tumors presented elevated phosphorylation levels of STING (p-STING), TBK1 (p-TBK1), and IRF3 (p-IRF3), showing 2.17-, 1.67-, and 1.72-fold increases, respectively, indicating more robust STING activation (Fig. S11A and B). These increases may have been due to more effective induction of tumor cell starvation following vascular normalization. An ELISA further revealed significantly elevated intratumoral IFN-β levels in GLA2-treated tumors, with a 2.13-fold increase compared with the GLA1-treated group (Fig. S11C and D), confirming that GLA2 robustly activated the STING pathway and downstream immune responses, which likely contributed to its enhanced antitumor efficacy.

Given the critical role of STING-induced IFN-β in the remodeling of the tumor immune microenvironment, particularly through DC activation [52], we next used FCM to analyze the immune cell composition within tumors (Figs. S12 and S13). The proportion of mature DCs was markedly greater in tumors from the GLA2-treated group than in those from the saline, AISIH, and GLA1 groups (2.71-, 1.70-, and 1.21-fold greater, respectively) (Fig. 6A). In particular, the proportion of CD103^+^ mature DCs (tumor-associated migratory cDC1s) was significantly greater in the GLA2 group than in the saline group (16.9% versus 7.61%; *P* < 0.001) (Fig. 6B). CD103^+^ cDC1s are critical for effective antigen presentation and the subsequent activation of effector T (Teff) cells [55,56]. Consistent with these findings, the proportion of CD4^+^ regulatory T (Treg) cells was significantly lower in tumors treated with GLA2 than in those treated with saline or GLA1 (Fig. 6C). Concurrently, the proportion of proliferating CD8^+^ Teff cells (Ki67^+^GzmB^+^CD8^+^) increased substantially following GLA2 treatment (21.1%) compared with those in the saline (6.4%; *P* < 0.001) and GLA1 (16.8%; *P* = 0.008) groups (Fig. 6D to F). Moreover, compared with saline treatment, GLA2 treatment significantly increased the abundance of antitumor M1 macrophages while reducing the number of pro-tumorigenic M2 macrophages (Fig. S14A and B). Collectively, these results indicate that the GLA2 strain elicits a potent immune activation response within the tumor microenvironment. Activated cDC1s effectively migrated to tumor-draining lymph nodes (TdLNs), where they further amplified antitumor immune activation [56] (Fig. 6G). FCM analysis of TdLNs further demonstrated enhanced DC activation following GLA2 treatment. GLA2 significantly increased the proportion of mature DCs among total DCs (58.7% versus 42.4% in the saline group, *P* < 0.001; versus 53.7% in the GLA1 group, *P* = 0.043). Consistently, GLA2 also increased the proportion of mature cDC1s (CD103^+^ mature DCs) to 59.0%, compared with 22.6% in the saline group (*P* < 0.001) and 47.5% in the GLA1 group (*P* = 0.044) (Fig. 6H and I). Notably, early activated CD69^+^CD8^+^ T cells were also significantly enriched in the TdLNs following GLA2 treatment (GLA2: 9.70% versus saline: 2.27%, *P* < 0.001; versus GLA1: 6.60%, *P* = 0.046) (Fig. 6J and K). In addition, the frequency of CD4^+^CD8^+^ double-positive T (DPT) cells, a subset known for potent antitumor cytotoxicity, was increased following GLA2 treatment. The proportion of early activated CD69^+^ DPT cells was also significantly greater in the GLA2 group (21.1% versus saline: 3.38%, *P* < 0.001; versus GLA1: 15.3%, *P* = 0.009) (Fig. 6K to M). The enrichment of early activated CD69^+^CD8^+^ T cells suggests efficient induction of antigen-specific cytotoxic T cell responses, which mediate tumor cell killing via GzmB secretion. Accordingly, GLA2 treatment significantly increased the proportions of both proliferating CD8^+^ Teff cells (Ki67^+^GzmB^+^) and CD4^+^ Teff cells (Ki67^+^GzmB^+^). Compared with the saline-treated control group (CD8^+^ Teff cells: 1.67%; CD4^+^ Teff cells: 3.72%), the GLA2 group presented a 6.23-fold increase in the proportion of proliferating CD8^+^ Teff cells (10.4%) and a 1.91-fold increase in the proportion of CD4^+^ Teff cells (7.11%) (Fig. 6N to P). These findings demonstrate that GLA2 effectively promotes T cell activation and differentiation toward tumor-killing phenotypes within TdLNs, which is consistent with its potent tumor-suppressive effects. Importantly, histopathological examination 7 d posttreatment revealed no significant pathological alterations in major organs, including the heart, liver, spleen, lungs, and kidneys, indicating favorable biosafety (Fig. S14C). In conclusion, these findings demonstrate that the engineered GLA2 strain enhances antitumor immunity by efficiently activating the intratumoral STING pathway, remodeling the immunosuppressive tumor microenvironment, and augmenting immune activation within both tumors and draining lymph nodes. Mechanistically, the GLA2 strain colonizes tumor tissues and proliferates within them post-administration, competing locally with tumor cells for glucose while secreting TNF-αnb to promote vascular normalization and increase nutrient deprivation. The resulting metabolic stress triggers STING activation in tumor cells and the release of proinflammatory cytokines, which, in concert with bacterial immune stimulation, drive the reprogramming of TdLN-resident immune cells—including DCs and T cells—toward potent antitumor phenotypes (Fig. 6Q).

**Fig. 6.**
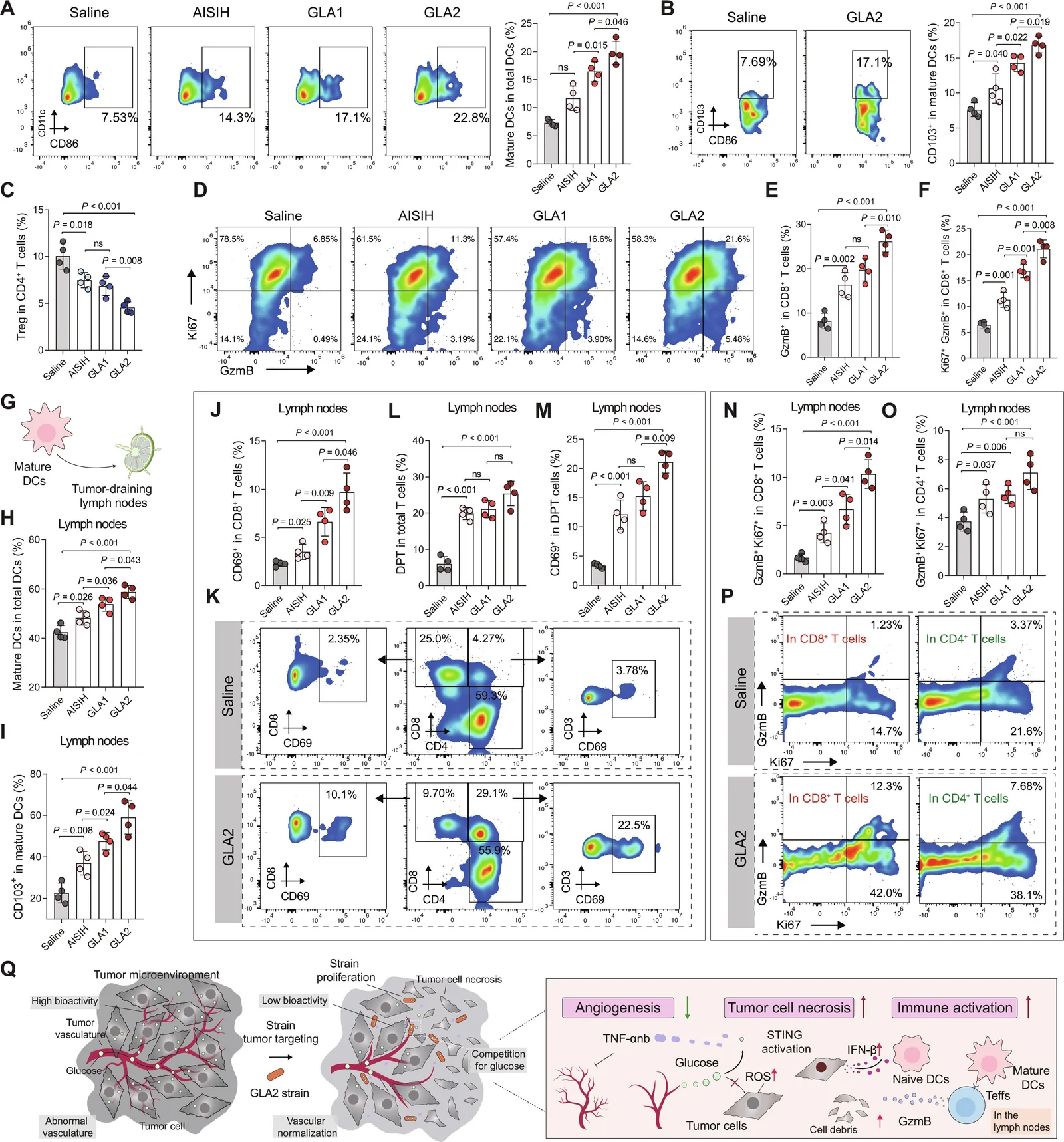
The GLA2 strain achieves potent intratumoral immune remodeling and lymph node activation. (A to P) Three days after the intraperitoneal administration of different strains to subcutaneous syngeneic bladder tumor-bearing model mice, changes in immune cells within tumors and TdLNs were detected in the saline, AISIH strain, GLA1 strain, and GLA2 strain treatment groups. (A) Representative FCM plot (right) and a bar comparison chart (left) showing the changes in the frequency of mature DCs (CD11c^+^CD86^+^) in tumors following different treatments (*n* = 4). (B) Representative FCM plot (left) and a bar comparison chart (right) of the changes in the frequency of mature cDC1s (CD103^+^CD11c^+^CD86^+^) in tumors subjected to different treatments (*n* = 4). (C) Changes in the frequency of CD4^+^ Treg cells (FoxB^+^CD4^+^) in tumors subjected to different treatments (*n* = 4). (D to F) A representative FCM plot (D) and a bar chart showing the changes in the frequencies of GzmB^+^CD8^+^ T cells (E) and proliferating GzmB^+^CD8^+^ T cells (GzmB^+^Ki67^+^CD8^+^) (F) in tumors subjected to different treatments (*n* = 4). (G) Schematic diagram of the migration of tumor-infiltrating mature DCs to TdLNs. (H) Changes in the frequency of mature DCs (CD11c^+^CD86^+^) in TdLNs after different treatments (*n* = 4). (I) Changes in the frequency of CD103^+^ mature DCs (CD103^+^CD11c^+^CD86^+^) in TdLNs after different treatments (*n* = 4). (J to M) Changes in the frequency of CD69^+^CD8^+^ T cells (J). (K) Representative FCM plot of saline and GLA2 groups. The proportion of CD4/CD8 DPT cells (L), and the proportion of CD69^+^ DPT cells (M) in TdLNs on day 3 posttreatment. (N to P) Changes in the frequencies of GzmB^+^Ki67^+^CD8^+^ T cells (N) and GzmB^+^Ki67^+^CD4^+^ T cells (O) in TdLNs on day 3 posttreatment. (P) Representative FCM plot of saline and GLA2 groups. (Q) Schematic of GLA2 strain antitumor therapy. The GLA2 strain proliferates specifically within tumors and competes with tumor cells for limited glucose. This process involves (1) secretion of TNF-αnb to inhibit abnormal vascular proliferation within tumors; (2) starvation of tumor cells to activate the STING pathway, which is accompanied by increased secretion of proinflammatory cytokines such as IFN-β; and (3) the enhanced effects of proinflammatory cytokines combined with the GLA2 strain in activating tumor-infiltrating DCs, promoting their maturation and migration to TdLNs, where they facilitate T lymphocyte activation and contribute to tumor cell elimination. The data are presented as the means ± SDs. All the data are representative of 2 independent experiments. ns, not significant; DC, dendritic cells; DPT, double-positive T cells; IFN-β, interferon-β; GLA1, *glpX*-deficient AISIH strain (Δ*glpX*-AISIH); GLA2, GLA1-derived strain expressing an anti-TNF-α nanobody under quorum-sensing control; TNF-α, tumor necrosis factor-α.

### The GLA2 strain exhibits potent in situ anticancer effects on bladder cancer

Next, we employed an MB49 in situ bladder cancer model to assess the clinical translational potential of the GLA2 strain. Mice bearing orthotopic MB49 tumors received intraperitoneal injections of the GLA1 and GLA2 strains (Fig. 7A). Bladder tissues collected 3 d posttreatment revealed that the GLA1 strain induced pronounced abnormal vascular proliferation within the bladder, whereas the GLA2 strain markedly alleviated this effect (Fig. 7B), which was consistent with the findings from subcutaneous tumor models (Fig. 5E and J). Intratumoral CD31 immunofluorescence staining further confirmed these results; compared with saline treatment, GLA1 treatment induced significant tumor vascular expansion, whereas GLA2 treatment effectively suppressed vascular proliferation (Fig. 7C). Tumor progression in live mice was subsequently monitored in real time via an in vivo imaging system. Compared with saline, the GLA1 and GLA2 strains exhibited rapid and substantial tumor growth inhibition. By day 5 posttreatment, the bladder tumor bioluminescence signals in the GLA1- and GLA2-treated groups were significantly lower than those in the control group and remained suppressed through day 13 (Fig. 7D and E). Quantitatively, the mean tumor bioluminescence intensities in the GLA1 and GLA2 groups on day 5 decreased to approximately 38.2% and 23.7% of the control levels, respectively (Fig. 7F). These results closely mirrored the antitumor effects observed in subcutaneous syngeneic models. Notably, the antitumor efficacy of the GLA2 strain was significantly stronger than that of the GLA1 strain. By day 13 posttreatment, the average tumor bioluminescence intensity in the GLA2 group was only 35.9% of that in the GLA1 group (Fig. 7G). Survival analyses revealed that compared with the control and GLA1 groups, which exhibited mean survival times of 19 and 31 d, respectively, the GLA2-treated group presented the most pronounced improvement, with an average survival duration of 37 d, 94.7% and 19.4% greater, respectively (Fig. 7H). Histopathological examination of bladder tissues via H&E staining revealed markedly increased vascularization in GLA1-treated tumors compared with that in saline control-treated tumors (Fig. 7I). In contrast, tumors from the GLA2-treated mice significantly regressed, with a visibly reduced tumor burden compared with those in both the saline- and GLA1-treated groups, confirming the superior therapeutic efficacy of the GLA2 strain against in situ bladder cancer (Fig. 7I).

**Fig. 7.**
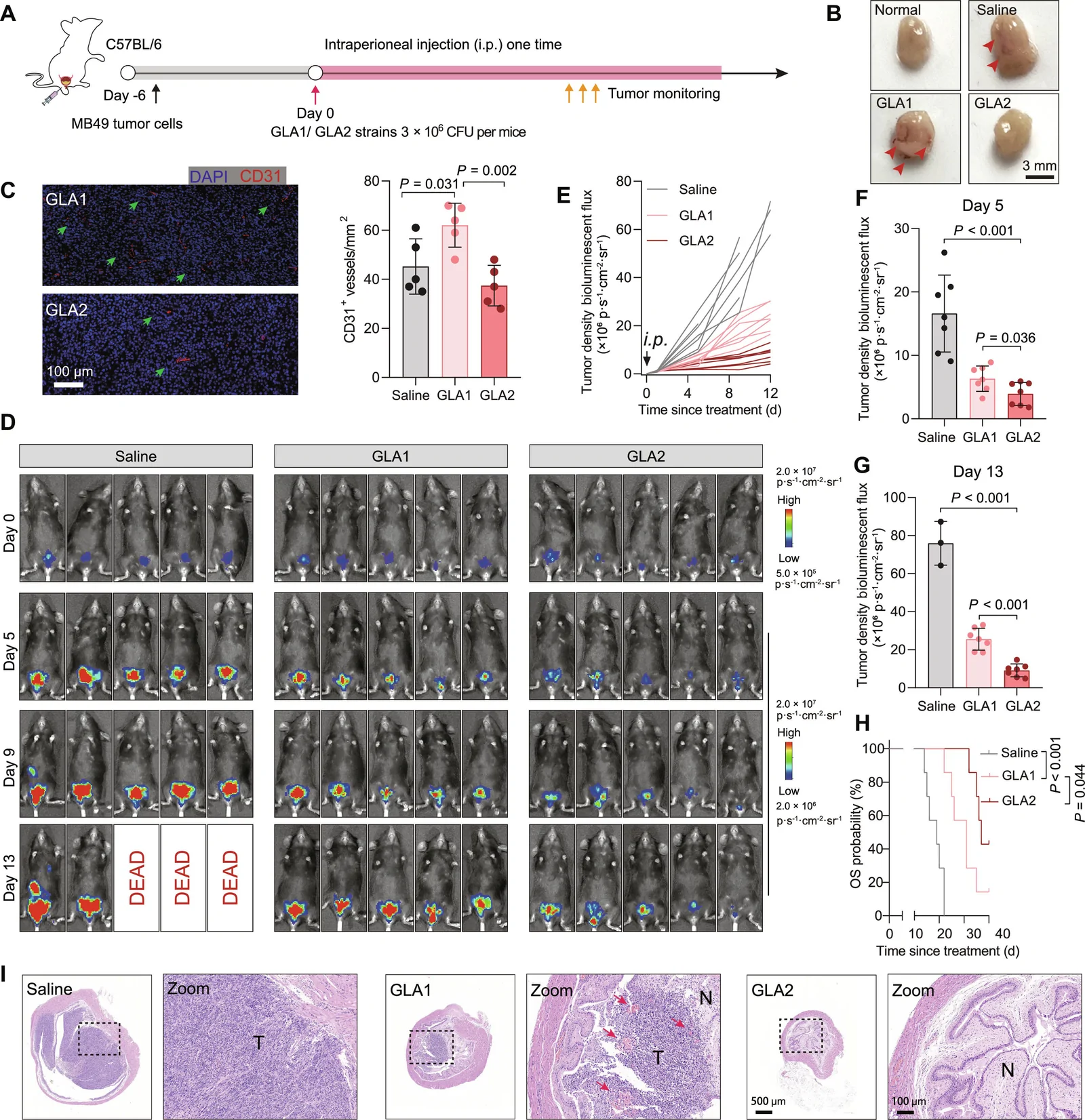
The GLA2 strain achieves potent in situ bladder cancer treatment. (A) Schematic of drug administration in mice with in situ bladder cancer. On day 6 post-tumor induction, the mice received intraperitoneal injection of saline, the GLA1 strain, or the GLA2 strain (3 × 10^6^ CFU per mouse), followed by in vivo imaging to monitor bladder tumor progression. (B) Differences in bladder histology 3 d after treatment in mice with in situ bladder cancer. Normal indicates bladder tissue from healthy mice; Saline indicates bladder tissue from tumor-bearing mice treated with saline; GLA1 indicates bladder tissue from tumor-bearing mice treated with the GLA1 strain; GLA2 indicates bladder tissue from tumor-bearing mice treated with the GLA2 strain. Red arrows indicate blood vessels within bladder tissue. Scale bar, 3 mm. (C) Representative immunofluorescence images of CD31 expression in bladder cancer tumor tissue (left) and quantitative analysis comparison (right). Scale bar, 100 μm. (D) Representative in vivo bioluminescence images of mice from the saline, GLA1, and GLA2 groups at the indicated time points. “DEAD” indicates mouse death. (E) Real-time bioluminescence intensity curve of mouse bladders from (D) (*n* = 7). (F and G) Comparison of bladder tumor tissue bioluminescence in groups from (E) on day 5 (F) and day 13 (G). (H) Survival curves of mice after tumor cell inoculation. The mice were euthanized upon reaching a humane endpoint (*n* = 7). (I) Representative H&E images of bladder cancer on day 7 posttreatment. The red arrows indicate intralesional vessels. “T” denotes a tumor, and “N” denotes necrosis. Scale bars, 500 μm (normal field) and 100 μm (magnified field). The data are presented as the means ± SDs. All the data are representative of 2 independent experiments. ns, not significant; H&E, hematoxylin and eosin staining; GLA1, *glpX*-deficient AISIH strain (Δ*glpX*-AISIH); GLA2, GLA1-derived strain expressing an anti-TNF-α nanobody under quorum-sensing control.

To evaluate the potential of metabolic competition therapy, the sensitivity of various tumor cells to glucose deprivation was assessed. Under low-glucose conditions, most tumor cells exhibited a rapid decline in viability (Fig. S15). After 24 h, the viability of MB49, LLC, and 4T1 cells decreased to approximately 8% of that under normal-glucose conditions, while B16F10, MC38, and S180 cells declined to 12.96%, 25.9%, and 29.7%, respectively. Panc02 cells showed relatively higher tolerance but still decreased to 38.50%, whereas H22 cells were the most resistant, maintaining 65.3% viability. These findings are consistent with a previous report [57] and indicate that most tumor cell types are highly sensitive to glucose deprivation. Thus, restricting glucose availability via metabolic competition represents a broadly applicable strategy for tumor suppression and underscores the potential of the GLA2 strain.

## Discussion

Bacteria-mediated antitumor therapy has gained increasing attention because of its low cost, sustained efficacy, and high scalability potential. The therapeutic mechanisms involve primarily toll-like receptor- or NOD-like receptor-dependent activation of tumor-infiltrating immune cells and direct induction of tumor cell apoptosis, both of which are under extensive investigation [1,5,9]. However, the influence of bacterial glycolytic metabolism on tumor metabolic reprogramming and its contribution to antitumor efficacy remain largely unexplored. In this study, we demonstrated for the first time that oncolytic bacteria profoundly disrupted glucose metabolism in tumor cells. Attenuated *S. Typhimurium* induced tumor cell starvation through both direct intracellular glucose deprivation and indirect extracellular nutrient competition, which was accompanied by elevated intracellular ROS levels, mitochondrial damage, and mtDNA release. Notably, we report that deletion of the glycolytic gene *glpX*, which encodes fructose-1,6-bisphosphatase, an enzyme responsible for converting fructose 1,6-bisphosphate into fructose 6-phosphate, further increases bacterial nutrient competition and markedly enhances anticancer efficacy. Attenuated *Salmonella*-induced tumor cell starvation subsequently activates the STING pathway via the mtDNA/STING/TBK1/IRF3 signaling axis, leading to robust proinflammatory cytokine release and DC maturation. These findings demonstrate that bacterial nutritional competition can trigger STING activation in tumor cells. Furthermore, we engineered *Salmonella* strains to controllably express and release a TNF-αnb via quorum sensing, thereby promoting tumor vascular normalization and exacerbating tumor cell starvation. The resulting GLA2 strain achieved potent immune activation within both tumors and TdLNs, resulting in synergistic antitumor efficacy in subcutaneous and in situ bladder cancer models.

Cancer cells, which are characterized by rapid proliferation, require large amounts of nutrients such as glucose and amino acids and often undergo metabolic pathway reprogramming to utilize available substrates. Thus, targeting cancer metabolism represents an attractive therapeutic strategy [15,18]. Previous studies have shown that *Salmonella* can inhibit tumor growth by modulating the tumor immune microenvironment. Following bacterial treatment, indoleamine 2,3-dioxygenase (IDO) levels decrease, gap junction levels increase, and the numbers of Tregs are reduced, which shifts the immune milieu toward a T helper 1 (Th1)-type response, with macrophage polarization shifting toward an M1-like phenotype [1,9]. Additionally, alterations in bacterial metabolic pathways profoundly influence bacterial antitumor activity. Disruption of the leucine or arginine biosynthetic pathway enhances the tumoricidal efficacy of *Salmonella* [58,59], and deletion of the *aroA* gene not only causes nutritional auxotrophy but also increases bacterial immunogenicity and antitumor potency [4]. Our findings reveal that in addition to amino acid metabolic pathways, glycolytic pathways are equally crucial for bacterial antitumor efficacy. Knockout of glycolytic genes—including *cra*, *ptsG*, *pfkB*, and *pykF*—partially or completely abrogated the antitumor effects of attenuated *Salmonella* against subcutaneously implanted bladder tumors. Conversely, the deletion of genes involved in gluconeogenesis, such as *yidA*, *yigL*, *glpX*, and *ppsA*, significantly increased the antitumor activity of *Salmonella*. We demonstrated that attenuated *Salmonella*-mediated nutrient competition triggered increased TNF-α production in tumor cells. Furthermore, immune cells that infiltrate tumors in response to bacterial stimulation often produce substantial amounts of TNF-α [1,50]. Interestingly, the clearance of TNF-α does not impair the anticancer effects of *Salmonella*; rather, these effects are moderately enhanced, which suggests that TNF-α derived from immune cells does not contribute to bacteria-mediated anticancer effects [51]. Therefore, we further utilized GLA2 to continuously secrete an anti-TNF-α nanobody within the tumor to enhance the *Salmonella*-induced nutrient competition effect by blocking abnormal vascular proliferation. In fact, previous studies have demonstrated that inhibiting TNF-α can effectively suppress tumors, for example, by using combination therapy with anti-TNF-α and cisplatin [48] or anti-TNF antibody therapy combined with nivolumab and ipilimumab [49]. These findings suggest that TNF-α from the tumor as a whole (including both tumor cells and intratumoral immune cells) may contribute to tumor progression, which warrants further investigation.

We confirmed that *Salmonella* with defective *glpX* exhibits superior antitumor efficacy by increasing the degree of competition between glucose and tumor cells. Given the metabolic heterogeneity among cancer types, we selected bladder cancer as the target for glucose deprivation, considering that BCG, another live biotherapeutic, is already approved as a first-line clinical treatment for bladder cancer [2] and that bladder tumors exhibit heightened sensitivity to metabolism-targeting interventions [14]. As shown in Fig. S1, transcriptomic analyses of tumor cells, including lung cancer cells (A549 and H2009) and cervical cancer cells (HeLa), which were treated with different bacterial strains, consistently revealed energy metabolism reprogramming, suggesting that microbe-mediated nutrient competition may represent a broadly conserved antitumor mechanism. We further demonstrated that both intracellular and extracellular bacteria effectively competed for glucose with tumor cells in mouse bladder tumors. Variations in the expression of glucose metabolism-related genes, such as glucose-6-phosphate dehydrogenase (G6PD) overexpression in lung cancer [60], have been reported across tumor types. Our study demonstrated that most tumor cells, including breast cancer, colorectal cancer, and melanoma cells, were highly sensitive to glucose deprivation in vitro, which is consistent with the data from a previous report [57]. These findings suggest that the anticancer strategy employed in this study, which relies on oncolytic bacteria to deprive tumor cells of glucose, may be applicable beyond the bladder cancer model used herein. However, the metabolic sensitivity of these tumor cells in vivo warrants further investigation. Moreover, whether *Salmonella*-mediated STING pathway activation occurs universally across tumors, and, if so, to what extent, remains to be clarified. We further demonstrated that glucose deprivation had a substantially weaker impact on immune cell activity than on tumor cell activity, suggesting that bacteria-mediated nutrient competition preferentially impairs tumor cells while largely preserving immune cell function. Moreover, research has revealed that bacteria were localized mainly within the tumor core hypoxic region [61], whereas immune cells were predominantly distributed in peripheral regions [62]. The spatial isolation of bacteria from immune cells within the tumor reduces the likelihood of competition between them for glucose. We observed that bacteria preferentially aggregated within tumor tissues, which is consistent with the findings of a previous report [63]. This localized bacterial enrichment facilitates the quorum sensing-dependent expression of functional proteins [63] and enhances local competition for glucose, thereby triggering robust STING activation in adjacent tumor cells. This mechanism provides a plausible explanation for the increased tumor cell apoptosis observed near bacterial clusters. Importantly, our findings expand the understanding of STING activation in tumor cells—not as a direct response to cytosolic DNA recognition but as a secondary consequence of bacterial competition for glucose. The resulting metabolic stress and mitochondrial damage promote ROS accumulation and mtDNA release, which in turn activate the cGAS/STING/TBK1/IRF3 cascade, amplifying antitumor immune responses. This unique paradigm of bacterially induced STING activation reveals a new dimension of how engineered microbes can reprogram the tumor microenvironment to sustain long-term antitumor immunity.

Despite these promising findings, several limitations should be acknowledged. The molecular mechanisms linking bacterial nutrient competition to mitochondrial dysfunction and mtDNA release require further investigation. In addition, although multiple cancer cell lines exhibited metabolic sensitivity to bacterial glucose competition, the therapeutic efficacy and STING-dependent immune activation should be validated in a broader range of clinically relevant tumor models, including patient-derived and spontaneous tumors. Furthermore, comprehensive evaluations of bacterial persistence, long-term biosafety, pharmacokinetics, and payload release characteristics will be essential before clinical translation. Addressing these questions will facilitate the further optimization and clinical development of metabolically engineered bacterial therapeutics.

In the future, the design of targeted, multifunctional, engineered bacterial strains, which are tailored to tumor profiles of individual patients, holds substantial promise. We demonstrated that the selective knockout of bacterial glucose metabolism genes directly modulated glucose uptake and consequently influenced the therapeutic efficacy of bacteria-mediated anticancer agents via competitive metabolic remodeling. Under hypoglycemic and hypoxic tumor conditions, cancer cells may utilize fatty acids or lipids as alternative energy sources for sustained proliferation [64]. Therefore, the regulation of other metabolites—including amino acids and lipids—warrants systematic investigation to elucidate their roles in both bacterial physiology and antitumor efficacy. This exploration is particularly relevant given the unique persistence and enrichment of bacteria within tumors, which often reach concentrations of 10^8^ CFU/g and persist for more than 14 d [3,26]. A plausible therapeutic hypothesis proposes that combining engineered bacterial strains with agents that modulate lipid metabolism could synergistically inhibit tumor growth through comprehensive nutrient deprivation. Additionally, tumor adaptive responses to starvation—such as autophagy-mediated nutrient recycling or senescence induction to resist metabolic stress—should be considered [15]. The combination of nutrient-blocking bacteria with autophagy inhibitors (e.g., chloroquine) may further potentiate anticancer effects. These strategies represent promising future directions for advancing metabolically engineered bacterial therapies for cancer.

## Ethical Approval

All animal experiments were approved by the Animal Care and Use Committee of Nanjing University (IACUC-2003167) and performed in accordance with institutional ethical guidelines.

## Data Availability

The raw sequencing data for this study have been deposited in the NCBI GEO (https://www.ncbi.nlm.nih.gov/geo/) under accession number GSE328957. Additional datasets used for this study were obtained from public repositories, including GSE53280 and GSE286573 from the GEO database (https://www.ncbi.nlm.nih.gov/geo/).
