# Supplementary material for: Metabolic Competition-Driven Antitumor Immunity of glpX-Deficient Engineered Salmonella via Glucose Deprivation and STING Activation in Bladder Cancer
Source: Cancer Commun (Lond). 2026 Oct 15;46:0060. doi: 10.34133/cancomm.0060 (PMC13669507; doi:10.34133/cancomm.0060)
Supplement: Supplementary file 1 [file cancomm.0060.f1.pdf]

Supplementary Materials for

Metabolic competition-driven antitumor immunity of *glpX*-deficient engineered *Salmonella* via glucose deprivation and STING activation in bladder cancer

Authors:

Leyang Wu[1,2,3,†,*], Xinyue Qiao[1,†], Wanru Wang[1], Jiahui Qiu[1], Xiao Dong[1], Liyuan Qiao[1], Chengxuan Lei[1], Xiaoxin Zhang[1], Ying Sun[1, 4], Chenyang Li[1], Bohao Wang[1], Zengzheng Du[1], Heng Liu[5], Zhengbing Lv[6], Xiaofeng Jiang[6*], Zichun Hua[1,2,3,7,8*]

Affiliations:

[1]State Key Laboratory of Pharmaceutical Biotechnology, School of Life Sciences, Nanjing University, Nanjing, Jiangsu, P. R. China.

[2]Nanjing Generecom Biotechnology Co., Ltd., Nanjing, Jiangsu, P. R. China.

[3]Changzhou High-Tech Research Institute of Nanjing University and Jiangsu TargetPharma Laboratories, Inc., Changzhou, Jiangsu, P. R. China.

[4]Department of Urology, Nanjing Drum Tower Hospital, Affiliated Hospital of Medical School, Nanjing University, Nanjing, Jiangsu, P. R. China.

[5]Yunnan Provincial Key Laboratory of Entomological Biopharmaceutical R&D, College of Pharmacy, Dali University, Dali, Yunnan, P.R. China.

[6]College of Life Sciences and Medicine, Zhejiang Provincial Key Laboratory of Silkworm Bioreactor and Biomedicine, Zhejiang Sci-Tech University, Hangzhou, Zhejiang, P. R. China.

[7]Faculty of Pharmaceutical Sciences, Xinxiang Medical University, Xinxiang, Henan, P. R. China.

[8]School of Biopharmacy, China Pharmaceutical University, Nanjing, Jiangsu, P. R. China.

*Corresponding author. Email:
Zichun Hua, zchua@nju.edu.cn;
Leyang Wu, lywu@nju.edu.cn;
Xiaofeng Jiang, xfjiang@zstu.edu.cn

†These authors contributed equally to this work.

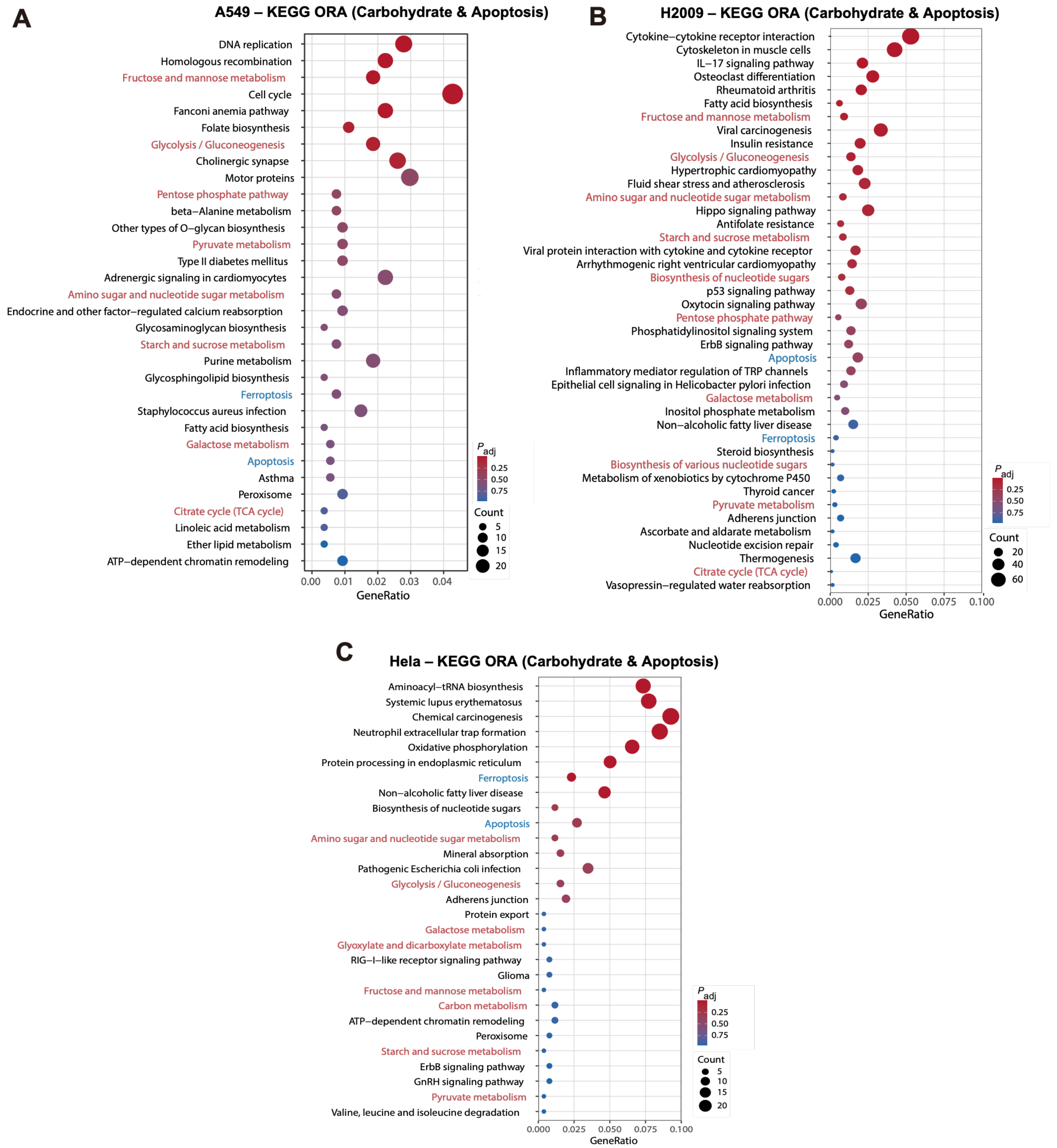

Fig. S1. Functional enrichment analysis of pathway alterations following bacterial infection of different tumor cell types.

(A-C) Functional enrichment analysis identified metabolic pathways significantly enriched in A549 tumor cells infected with *E. coli* (A), H2009 tumor cells infected with *E. coli* (B), and HeLa tumor cells infected with *S. Typhimurium* (C) compared with uninfected tumor cells. Pathways related to apoptosis are highlighted in blue, whereas those associated with carbohydrate metabolism are highlighted in red. Abbreviations: *E. coli, Escherichia coli*; *S. Typhimurium*, *Salmonella enterica serovar* Typhimurium; KEGG ORA, Kyoto Encyclopedia of Genes and Genomes Over-Representation Analysis.

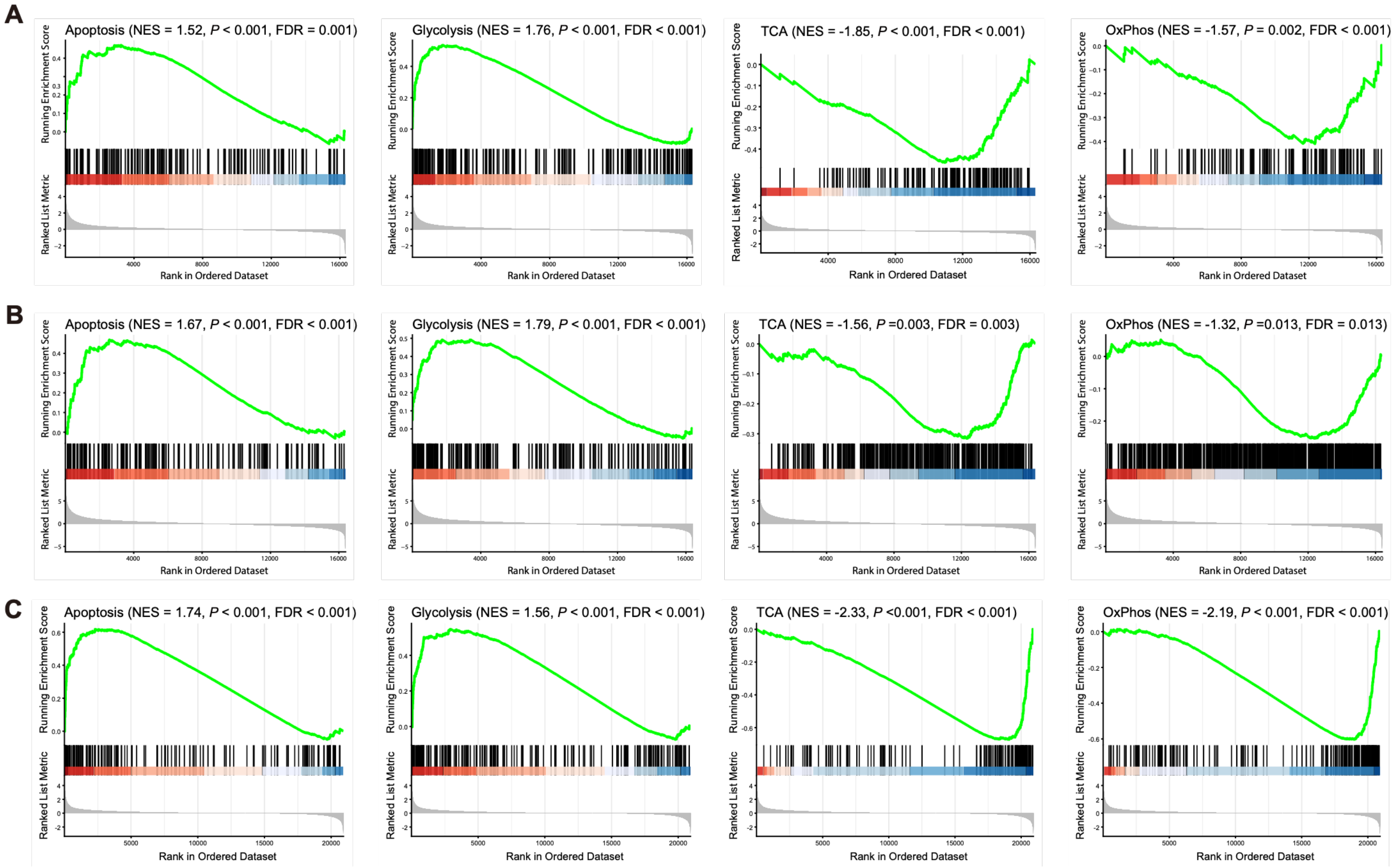

Fig. S2. Alterations in cellular pathways following bacterial infection of different tumor cell types. **(A-C)** GSEA using curated gene sets from the Molecular Signatures Database (MSigDB) revealed enrichment changes in the apoptosis, glycolysis, TCA cycle, and oxidative phosphorylation pathways following bacterial infection of different tumor cell types. (A) *E. coli* infection of A549 tumor cells, (B) *E. coli* infection of H2009 tumor cells, and (C) *S. Typhimurium* infection of HeLa tumor cells. Abbreviations: *E. coli*, *Escherichia coli*; *S. Typhimurium*, *Salmonella enterica serovar* Typhimurium; GSEA, gene set enrichment analysis; TCA, tricarboxylic acid; OxPros, oxidative phosphorylation; KEGG ORA, Kyoto Encyclopedia of Genes and Genomes over-representation analysis; FDR, false discovery rate; NES, normalized enrichment score; MSigDB, molecular signatures database.

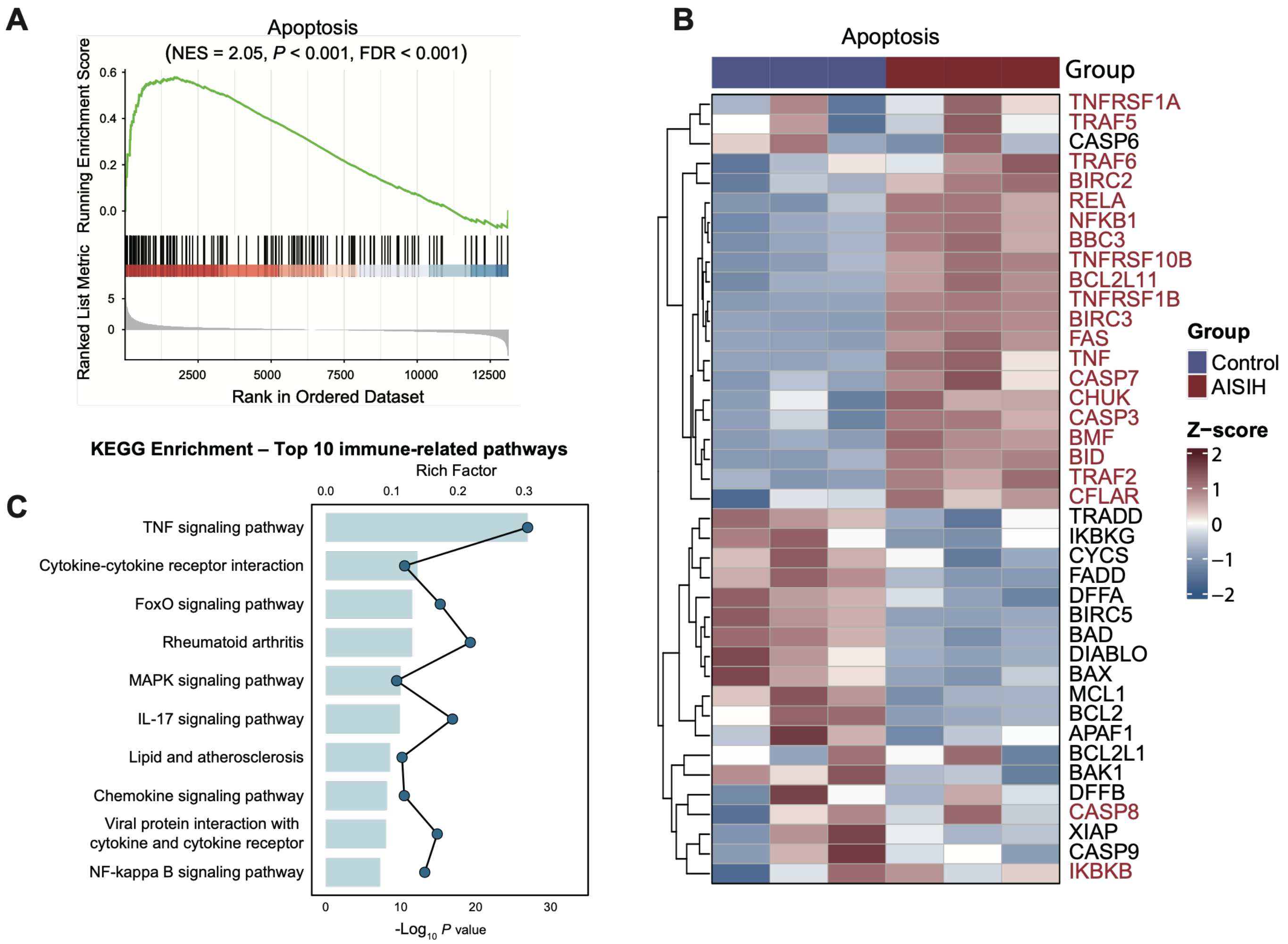

Fig. S3. Infection of MB49 tumor cells with attenuated *Salmonella* AISIH induces the upregulation of apoptosis signaling pathways.

(A) GSEA using curated gene sets from the MSigDB revealed significant positive enrichment of apoptosis-related gene sets in AISIH-treated MB49 tumor cells compared with untreated control cells. **(B)** Clustering heatmap of differentially expressed representative genes from the apoptosis signaling pathway in AISIH-treated MB49 tumor cells. Genes with established pro-apoptotic functions (marked in red) were generally upregulated in AISIH-treated tumor cells. **(C)** Ranking of activated immune-related pathways in tumor cells identified by KEGG enrichment analysis. Abbreviations: KEGG, Kyoto Encyclopedia of Genes and Genomes; GSEA, gene set enrichment analysis; FDR, false discovery rate; NES, normalized enrichment score; MSigDB, molecular signatures database.

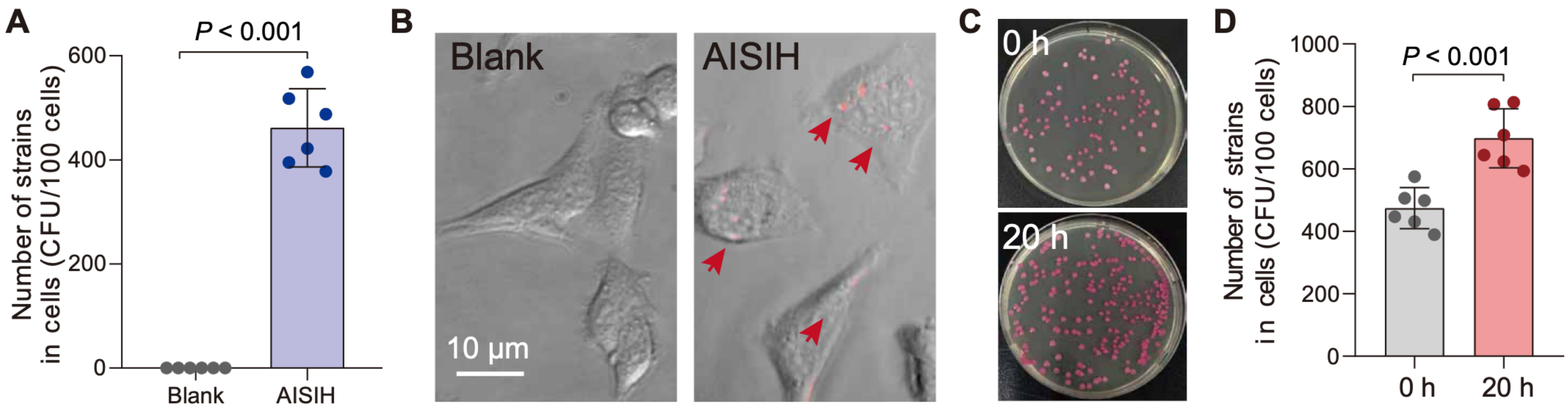

Fig. S4. Attenuated *Salmonella* can rapidly infect MB49 tumor cells

(A) Attenuated *Salmonella* strain AISIH was co-incubated with MB49 bladder cancer cells at an MOI of 100 for 4 hours. Extracellular free bacteria were killed with gentamicin, and intracellular bacterial counts were determined by plating (n = 6). **(B)** Representative microscopy image of *Salmonella* strain AISIH infection of tumor cells from panel (A). An engineered strain expressing red fluorescent protein was used. Red arrows indicate bacteria within tumor cells. **(C-D)** Representative images (C) and quantification (D) of viable intracellular bacteria after the plating of tumor cells infected with *Salmonella* from panel a and cultured for 20 hours in media supplemented with gentamicin (n = 6). *Salmonella* can persist and proliferate within tumor cells over extended periods. The data are reported as the means ± SDs. All the data are representative of two independent experiments. Abbreviations: MOI, multiplicity of infection; CFU, Colony-Forming Units.

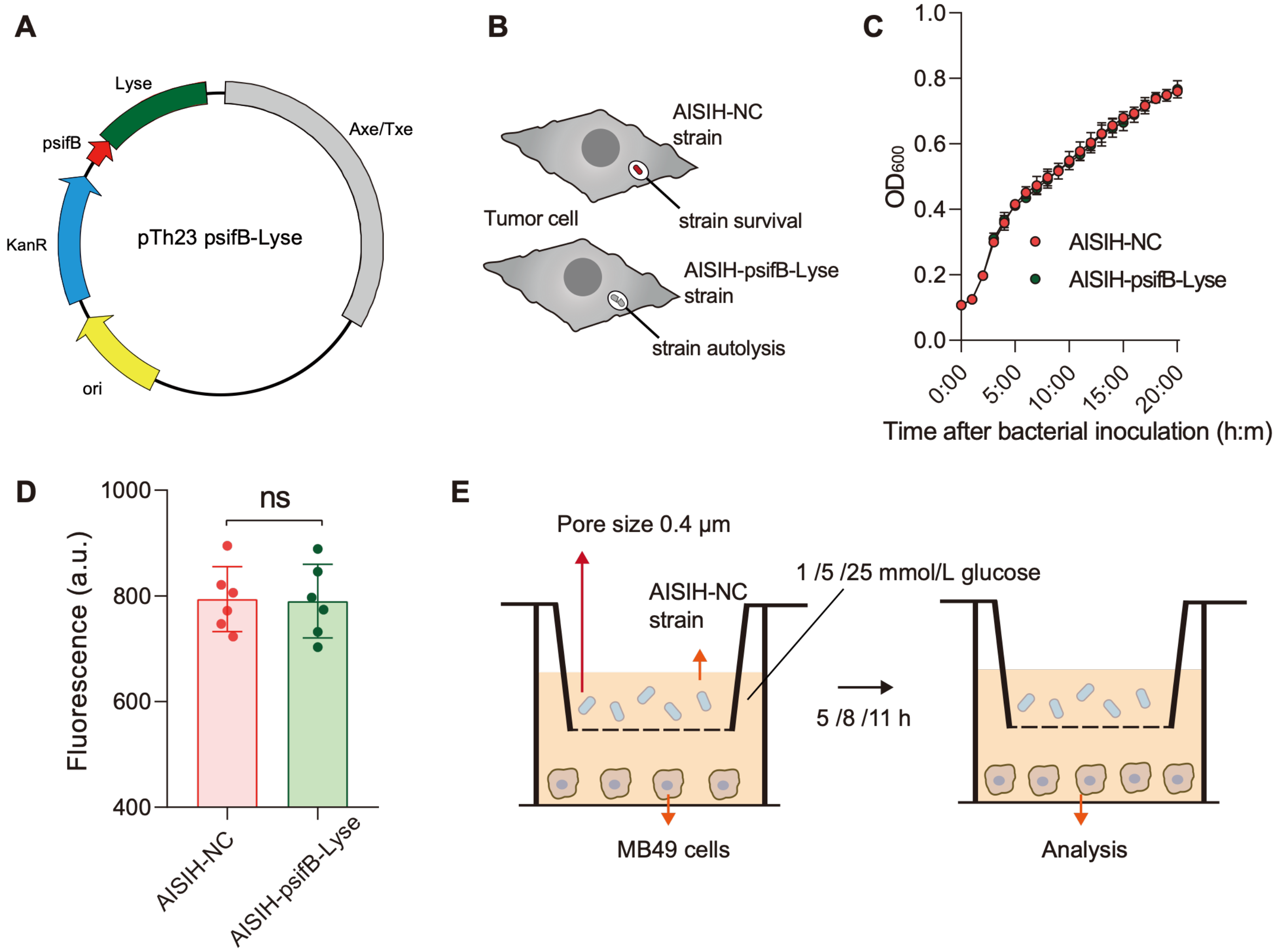

Fig. S5. Construction and evaluation of the AISIH-psifB-Lyse strain.

(A) Schematic of the engineered psifB-Lyse plasmid containing the *sifB* promoter, the target protein Lyse, and the anti-loss element Axe/Txe. The engineered plasmid was electro transformed into the AISIH strain to obtain the engineered strain AISIH-psifB-Lyse. **(B)** Schematic representation of the AISIH-NC (AISIH strain carrying an empty vector) and AISIH-psifB-Lyse strain within tumor cells. AISIH-NC can persistently survive within tumor cells. The AISIH-psifB-Lyse strain triggers Lyse protein expression via the *sifB* promoter due to the specific intracellular environment, leading to lysis and death of intracellular bacteria. The AISIH-NC served as a control. **(C)** Schematic comparison of growth curves between AISIH-NC and AISIH-psifB-Lyse strains (n = 3). **(D)** Comparison of direct 2-NBDG uptake capacity between AISIH-NC and AISIH-psifB-Lyse strains. No significant difference in glucose uptake capacity was observed between the two strains (n = 6). **(E)** Schematic diagram of the indirect co-culture system between tumor cells and the AISIH strain (MOI = 100). The MB49 tumor cells were indirectly co-cultured with the AISIH strain using a 0.4 μm pore size Transwell system in medium added with 1/5/25 mmol/L glucose. Cell proliferation was analyzed by measuring the total fluorescence intensity of the cells (stained with Hoechst) in the lower chamber 5, 8, and 11 hours later. The data are reported as the means ± SDs. All the data are representative of two independent experiments. ns, not significant. Abbreviations: MOI, multiplicity of infection; 2-NBDG, 2-[N-(7-Nitrobenz-2-oxa-1,3-diazol-4-yl)amino] -2-deoxy-D-glucose; a.u.arbitrary units.

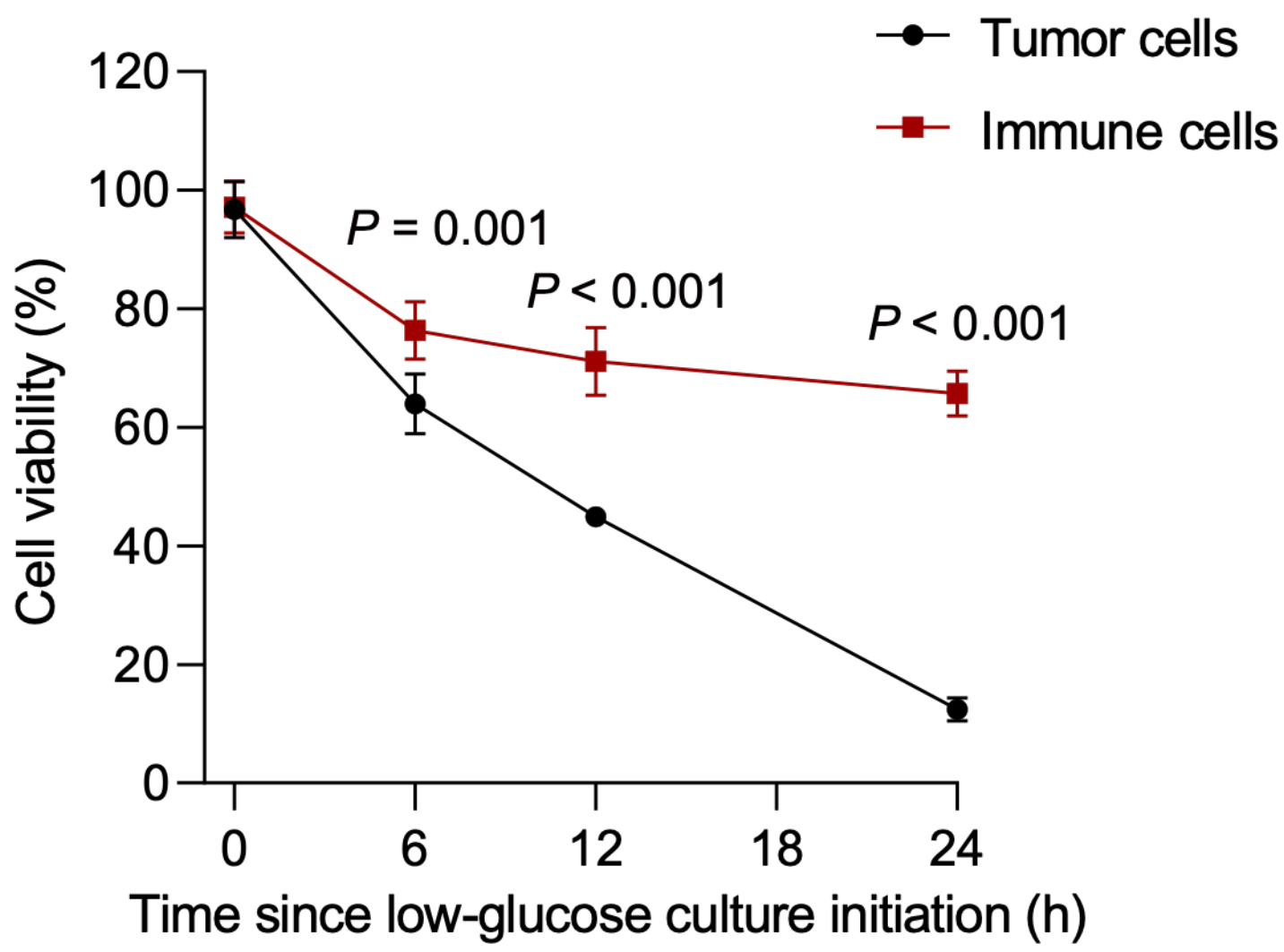

Fig. S6. The relative cell viability assay of MB49 tumor cells and murine splenic immune cells after culturing under low-glucose (1 mmol/L) conditions for different times (n = 5). The CCK-8 was used to measure the cell viability at 0 h, 6 h, 12 h, and 24 h of culture. Cell viability = (OD_{450} measured under low-glucose conditions − OD_{450} of blank medium) / (OD_{450} measured under normal-glucose conditions − OD_{450} of blank medium) × 100%. Normal-glucose conditions: 25 mmol/L. The data are reported as the means ± SDs. All the data are representative of two independent experiments. Abbreviations: CCK-8, cell counting kit-8.

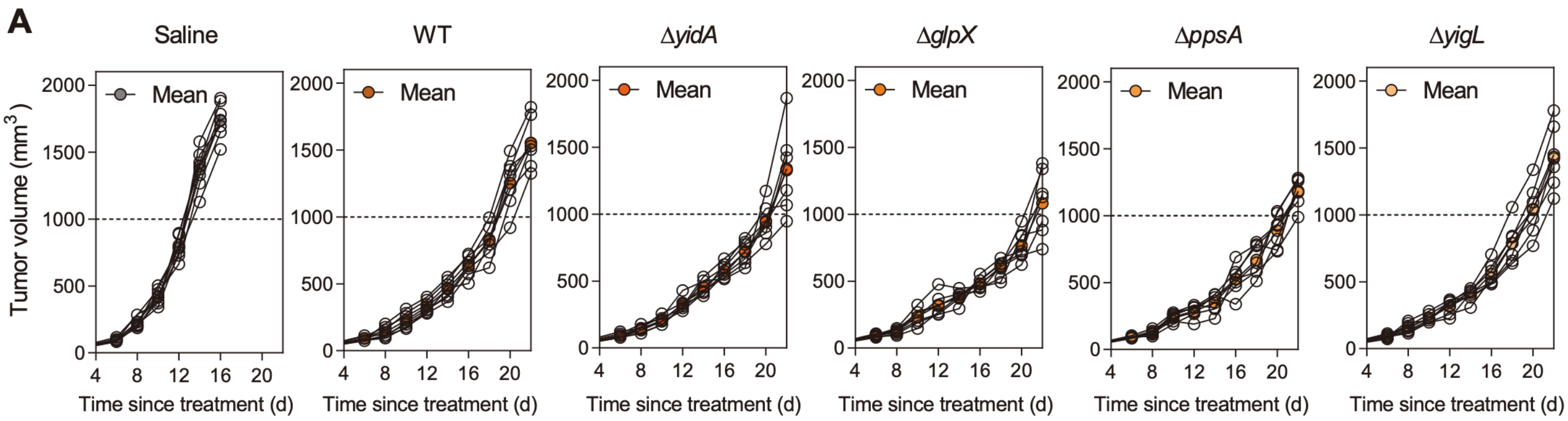

B

Group	Tumor doubling time
Saline	2.391 ± 0.1378 day
WT	3.873 ± 0.1922 day
Δ*yidA*	4.159 ± 0.3330 day
Δ*glpX*	4.602 ± 0.6203 day
Δ*ppsA*	4.324 ± 0.1871 day
Δ*yigL*	4.030 ± 0.2044 day

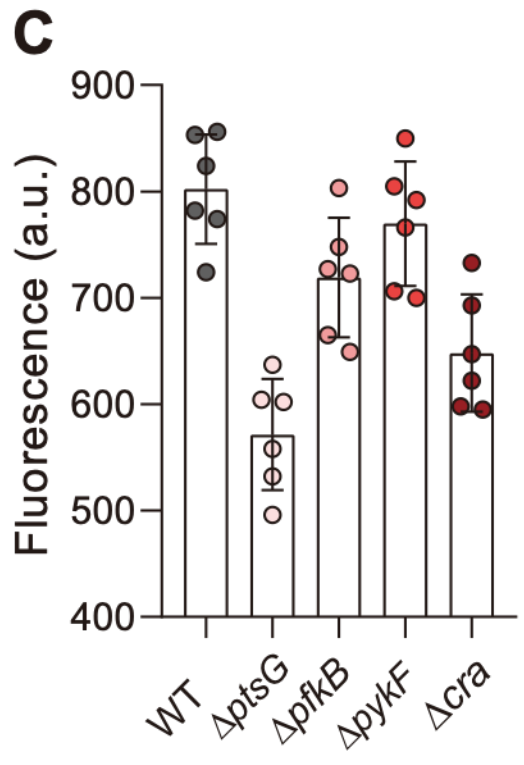

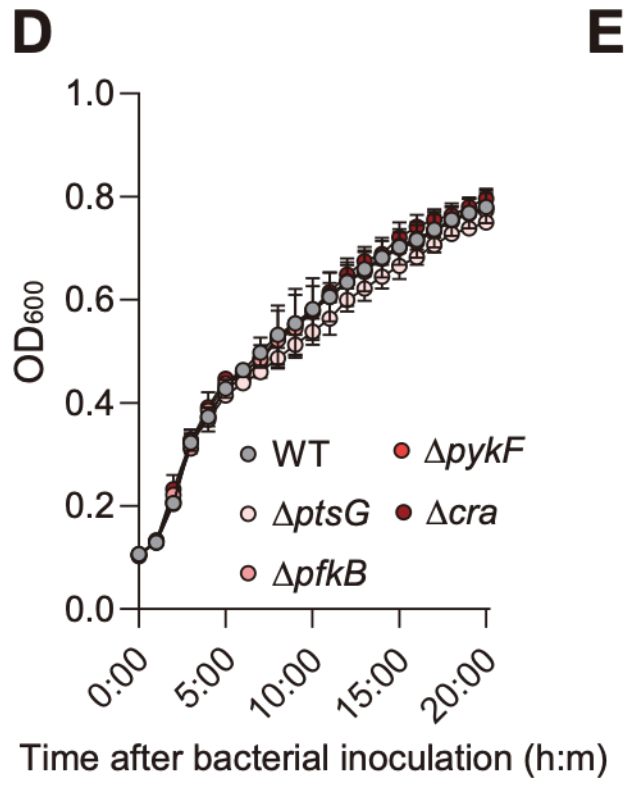

E

Group	Tumor doubling time
Saline	2.362 ± 0.1489 day
WT	3.773 ± 0.3182 day
Δ*ptsG*	2.366 ± 0.1133 day
Δ*pfkB*	3.515 ± 0.1581 day
Δ*pykF*	3.275 ± 0.1095 day
Δ*cra*	3.319 ± 0.1034 day

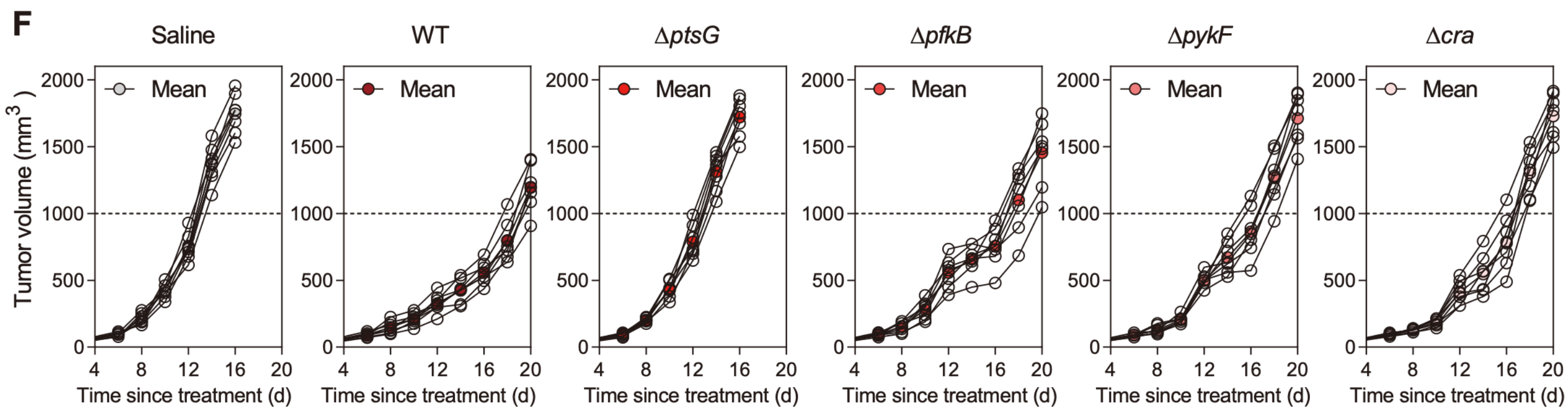

Fig. S7. Evaluation of the antitumor effects of AISIH and its glycolytic and gluconeogenic mutant strains.

(A) Tumor growth curves for each mouse in the different groups in Fig. 3G (*n* = 7). **(B)** Comparison of the tumor volume doubling times in the different groups in Fig. 3I (*n* = 7). **(C)** Fluorescence intensity of several strains was detected at the most significant emission wavelength of 542 nm via a fluorescence microplate reader after 30 minutes of coculture of AISIH and its glycolysis-mutant strains with 2-NBDG (excitation wavelength: 488 nm). All four mutant strains presented weaker glucose uptake capacities than did the wild-type strain. **(D)** Comparison of growth curves for the five attenuated *Salmonella* strains in liquid medium (*n* = 3). The defective strains presented similar growth rates. **(E)** Comparison of the tumor doubling times among the different groups in Fig. 3J (*n* = 7). **(F)** Tumor growth curves for each mouse in the different groups in Fig. 3H (*n* = 7). The data are reported as the means ± SDs. All the data are representative of two independent experiments. Abbreviations: 2-NBDG, 2-[N-(7-Nitrobenz-2-oxa-1,3-diazol-4-yl) amino] -2-deoxy-D-glucose; *ptsG,* phosphotransferase system glucose-specific transporter component; *pfkB*, phosphofructokinase B; *pykF*, pyruvate kinase F; *cra*, catabolite repressor/activator; *yidA*, putative phosphatase YidA; *yigL*, haloacid dehalogenase-like hydrolase YigL; *glpX*, fructose-1,6-bisphosphatase II; *ppsA*, phosphoenolpyruvate synthase.

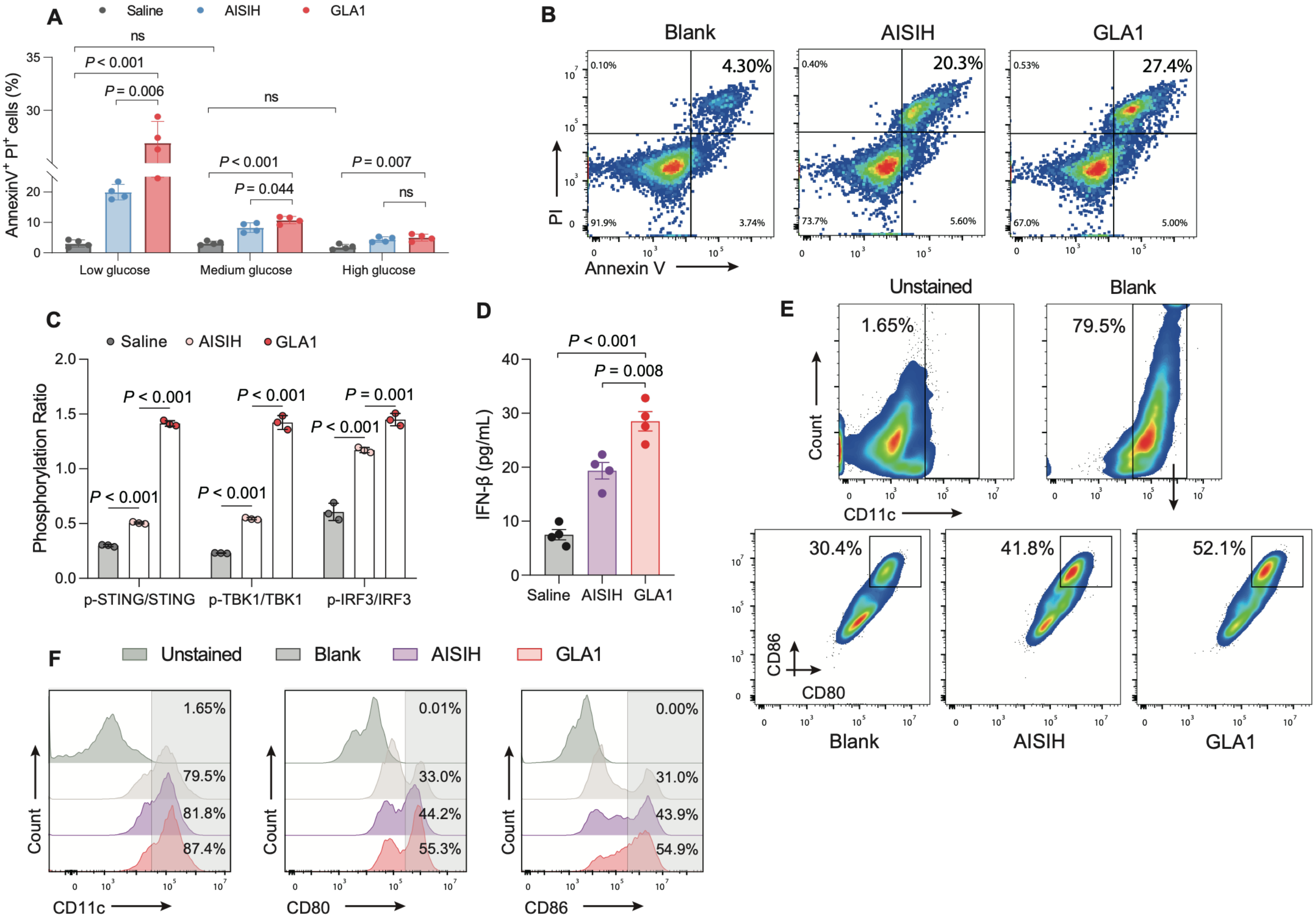

Fig. S8. Attenuated *Salmonella* more effectively induces apoptosis in starved tumor cells.
(A) Flow cytometry analysis of the proportion of late apoptotic tumor cells after different treatments at low (1 mmol/L), medium (5 mmol/L), and high (25 mmol/L) glucose concentrations. Attenuated *Salmonella* strains, including AISIH and GLA1, were cocultured with MB49 tumor cells at an MOI of 100 for 6 hours. **(B)** Representative flow cytometry image of cells treated with low glucose. **(C)** The quantitative analysis of protein expression according to Western blot analysis in cells in Fig. 4G were compared. **(D)** Detection of IFN-β levels in the mixed supernatants of tumor cells subjected to different treatments for 8 hours under low-glucose conditions (MOI 100, with 4×10^6 tumor cells in 1.0 mL culture medium). The GLA1 strain effectively promoted the production of IFN-β ($n = 4$). **(E)** After co-incubating AISIH and GLA1 with MB49 tumor cells for 12 hours, the culture supernatants were collected and incubated with BMDCs for an additional 24 h. Representative flow cytometry plots and quantitative analysis of $CD11c^+CD80^+CD86^+$ BMDCs are shown. **(F)** Histogram analysis of $CD11c^+$, $CD80^+$, and $CD86^+$ cells measured by flow cytometry after co-incubation of BMDCs with supernatants from tumor cells treated with GLA1 or AISIH. "Unstained" refers to unstained BMDCs that were not labeled with fluorescent antibodies and served as the negative control for flow cytometry. The data are reported as the means ± SDs. All the data are representative of two independent experiments. * $P < 0.05$; ** $P < 0.01$; ns, not significant. Abbreviations: BMDC, bone marrow-derived dendritic cells; IFN-β, interferon-β; MOI, multiplicity of infection.

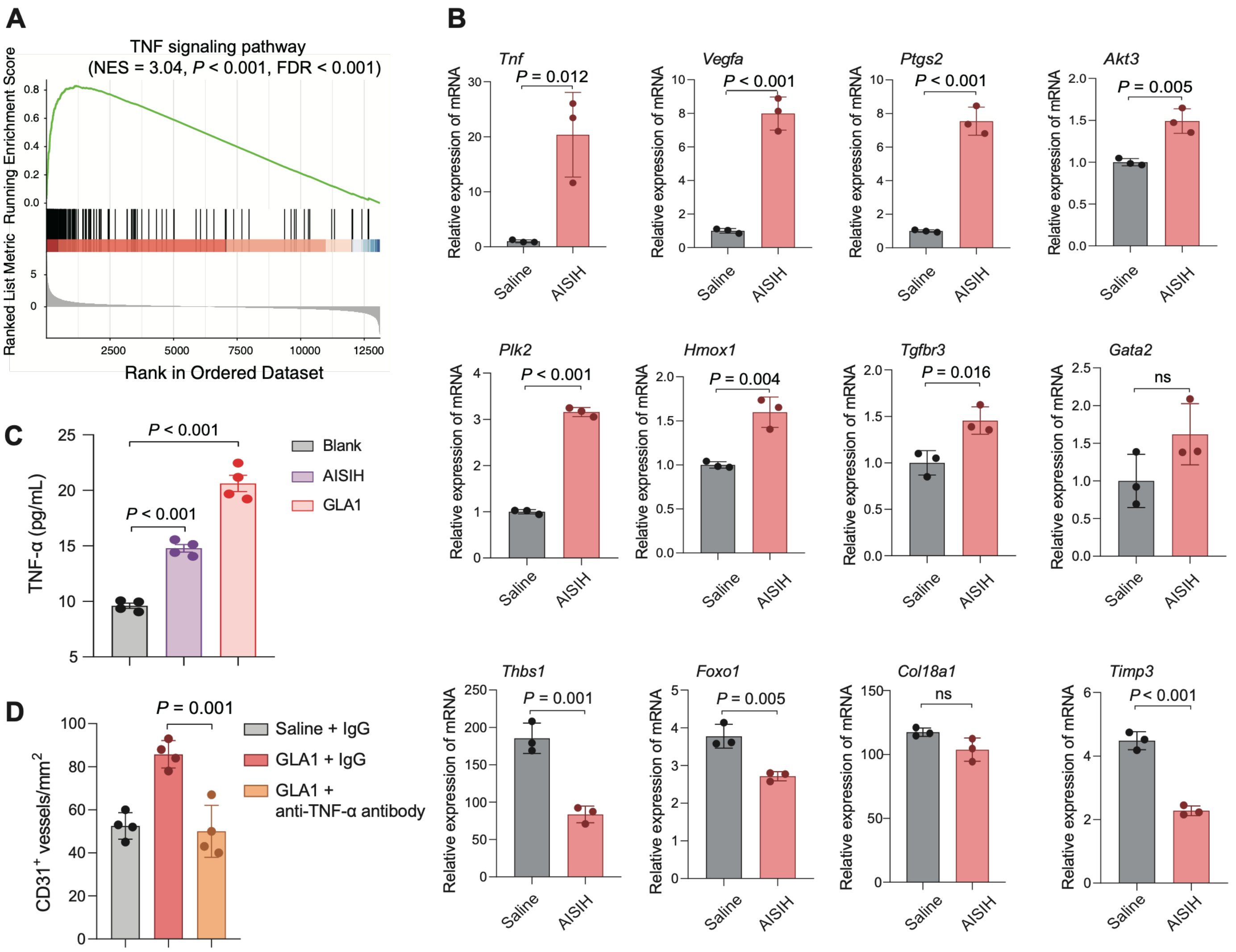

Fig. S9. Attenuated *Salmonella* induces abnormal intravascularization in bladder cancer tumors. **(A)** GSEA of the TNF signaling pathway gene set from MSigDB using RNA-seq dataset of AISIH-treated and control MB49 tumor cells presented in Figure 1. **(B)** Comparison of the expression of several representative proangiogenic genes, including *Tnf*, *Vegfa*, *Ptgs2*, *Plk2*, *Hmox1*, *Akt3*, and *Gata2* and anti-angiogenic genes, including *Tgfbr3, Thbs1, Foxo1, Col18a1, Timp3.* Attenuated *Salmonella* significantly promotes the upregulation of proangiogenic genes. **(C)** TNF-α cytokine detection in MB49 tumor cell culture supernatants after 8 hours of treatment with saline, the AISIH strain, or the GLA1 strain (n = 4). **(D)** GLA1-induced abnormal intratumoral vascular proliferation was effectively reduced by the TNF-α neutralizing antibody (n = 4). IgG (control) or the anti-TNF-α antibody (0.5 mg per mice) was injected intraperitoneally. The data are reported as the means ± SDs. All the data are representative of two independent experiments. * $P < 0.05$; ** $P < 0.01$; *** $P < 0.001$; n.s., not significant. Abbreviations: GSEA, gene set enrichment analysis; *Tnf*, tumor necrosis factor; *Vegfa*, vascular endothelial growth factor; *Ptgs2*, prostaglandin-endoperoxide synthase 2; *Tgfbr3*, transforming growth factor beta receptor 3; *Plk2*, polo-like kinase 2; *Hmox1*, heme oxygenase 1; *Akt3*, AKT serine/threonine kinase 3; *Gata2*, GATA binding protein 2, *Thbs1*, Thrombospondin 1; *Col18a1*, Collagen Type XVIII Alpha 1 Chain; *Timp3*, TIMP Metallopeptidase Inhibitor 3; *Foxo1,* forkhead box O1.

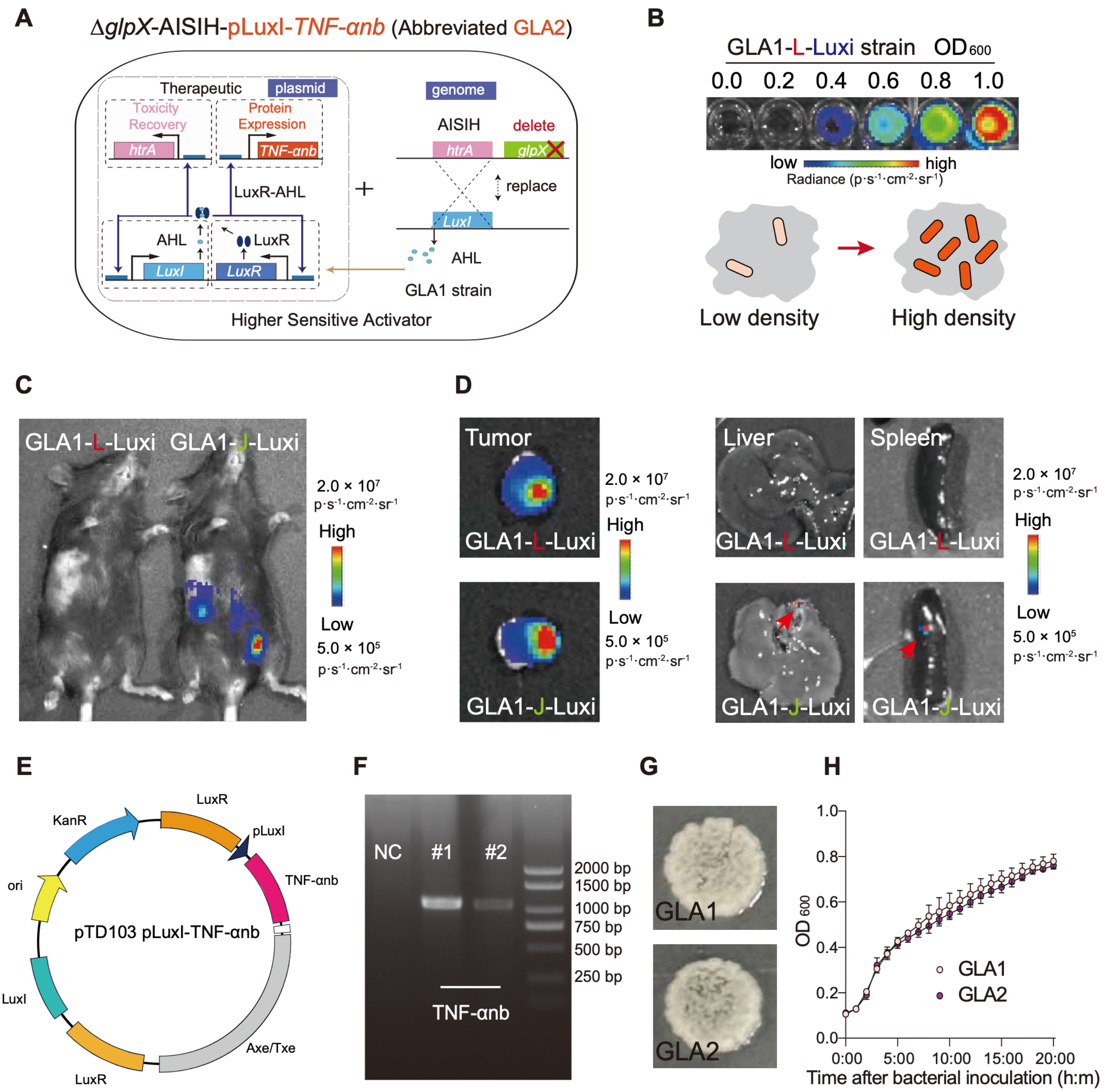

Fig. S10. Construction and Evaluation of the GLA2 Strain.

(A) Schematic of the controllable virulence recovery circuit and TNF-α nanoantibody production based on AISIH chassis bacteria. Regulation of the *htrA* gene and TNF-α gene expression by the LuxI promoter achieves bacterial HtrA and TNF-αnb expression only at high population densities. **(B)** In vitro imaging detection of quorum sensing-mediated LuxCDABE (Luxi) protein expression in the strain. The engineered strains initiated Luxi protein expression only at relatively high population densities. **(C)** In vivo imaging schematic 3 h after the intraperitoneal administration of different strains, including GLA1-L-Luxi (the engineered strain expressing the Luxi protein via the quorum-sensing LuxI promoter) and GLA1-J-Luxi (the engineered strain expressing the Luxi protein via the constitutive J23100 promoter). Significant uncontrolled off-target protein expression was observed in the GLA1-J-Luxi strain. **(D)** Tumor bioluminescence imaging at 24 hours (left) versus healthy organs (liver and spleen, right) in (C). Both the GLA1-L-Luxi and GLA1-J-Luxi strains presented robust Luxi protein expression within tumors, but the GLA1-J-Luxi group presented faint off-target protein expression detectable in the liver and spleen. Red arrows indicate the locations of bacterial enrichment in the corresponding organs. **(E)** Schematic diagram of the key plasmid (pTD103 pLuxI-TNF-αnb) used for the quorum sensing expression system. **(F)** Gel electrophoresis

verification of nucleic acids from the GLA1 strain, confirming the presence of the TNF-αnb sequence. #1 and #2 represent two monoclonal strains. The strain carrying a blank plasmid (NC) served as a control. **(G)** Comparative image of representative single colony morphologies of the GLA1 and GLA2 strains grown on LB agar plates after incubation at 37°C for 18 h. Plates were incubated in an inverted position. **(H)** Comparative growth curves of the GLA1 and GLA2 strains (n = 3). The data are reported as the means ± SDs. All the data are representative of two independent experiments. Abbreviations: GLA1, *glpX*-deficient AISIH strain (Δ*glpX*-AISIH); GLA2, GLA1-derived strain expressing an anti-TNF-α nanobody under quorum-sensing control; Luxi, LuxCDABE; GLA1-L-Luxi, the engineered strain expressing the Luxi protein via the quorum-sensing LuxI promoter; GLA1-J-Luxi, the engineered strain expressing the Luxi protein via the constitutive J23100 promoter.

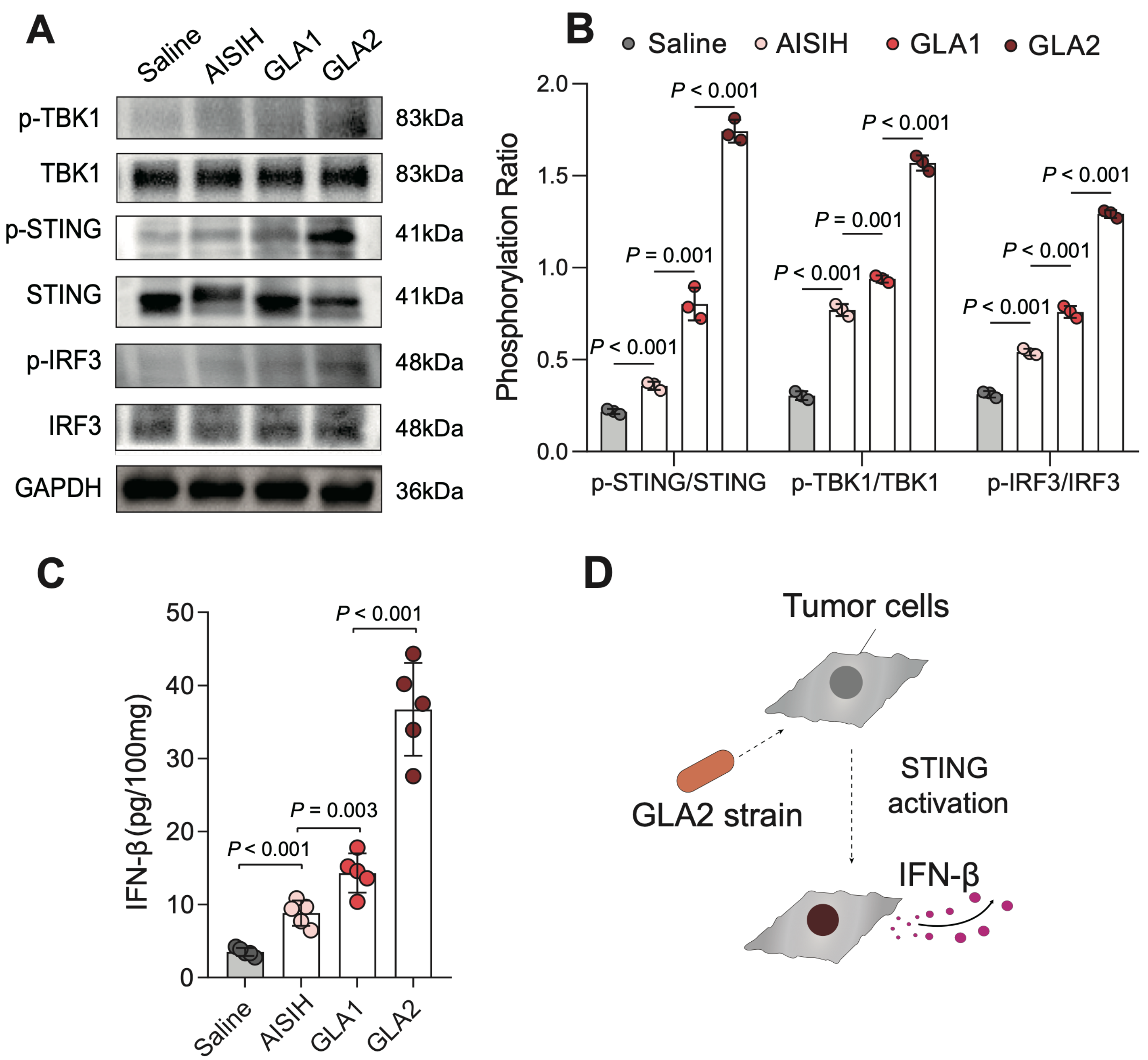

Fig. S11. The GLA2 strain activates the STING pathway in tumors.

(A) Western blot analysis of STING activation in tumor tissues 3 days after different treatments. **(B)** The quantitative analysis of protein expression according to Western blotting analysis in cells in (A) were compared. **(C)** IFN-β cytokines in tumor tissues 3 days after different treatments were detected (n = 5). **(D)** Schematic diagram of the induction of IFN-β secretion by tumor cells by the GLA2 strain. The data are reported as the means ± SDs. All the data are representative of two independent experiments. ns, not significant. Abbreviations: IFN-β, Interferon-β; STING, stimulator of interferon genes; IRF3, Interferon regulatory factor 3; TBK1, TANK-binding kinase 1; GLA1, glpX-deficient AISIH strain (Δ*glpX*-AISIH); GLA2, GLA1-derived strain expressing an anti-TNF-α nanobody under quorum-sensing control.

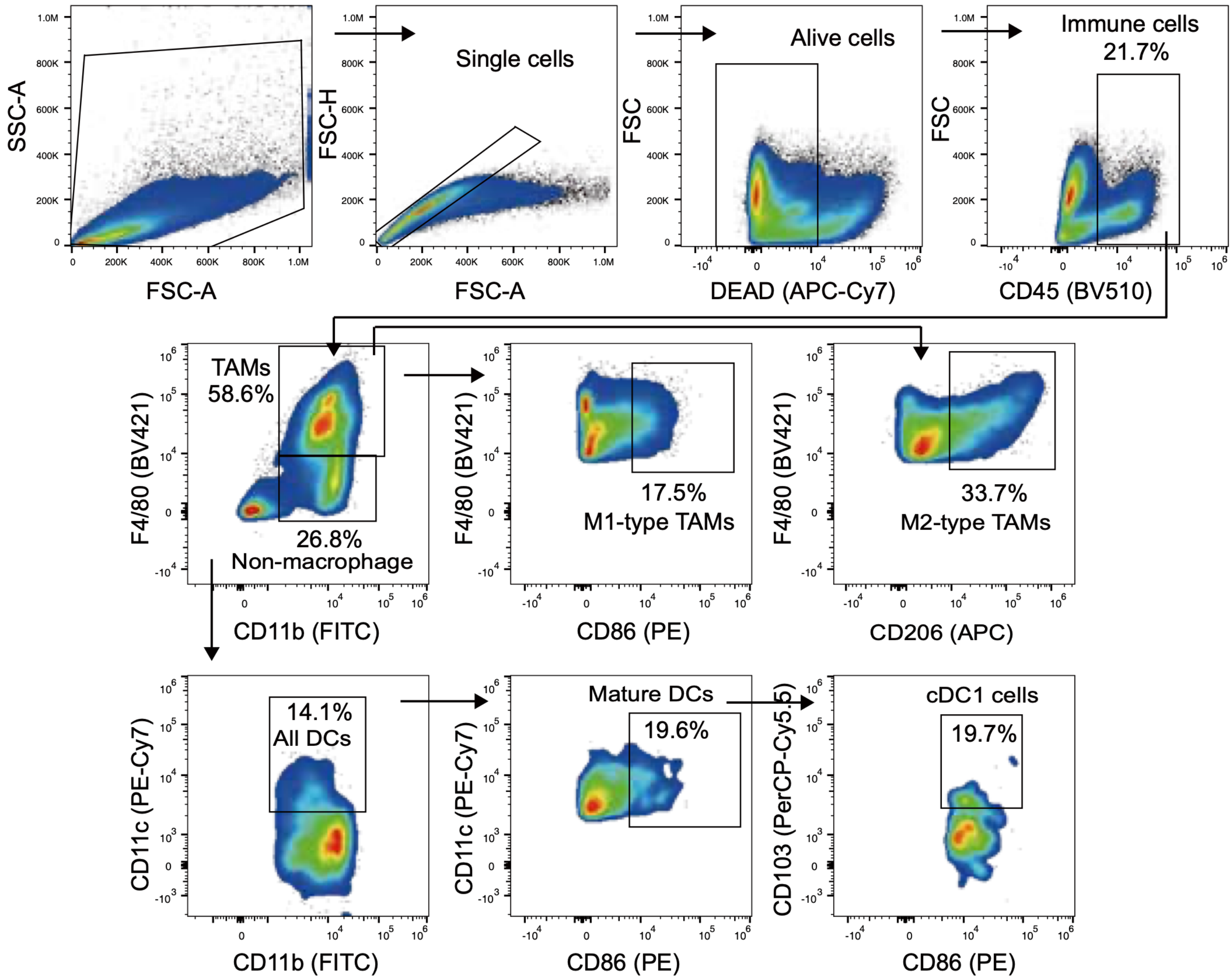

Fig. S12. Representative gating strategy for identifying monocyte cells with different phenotypes.

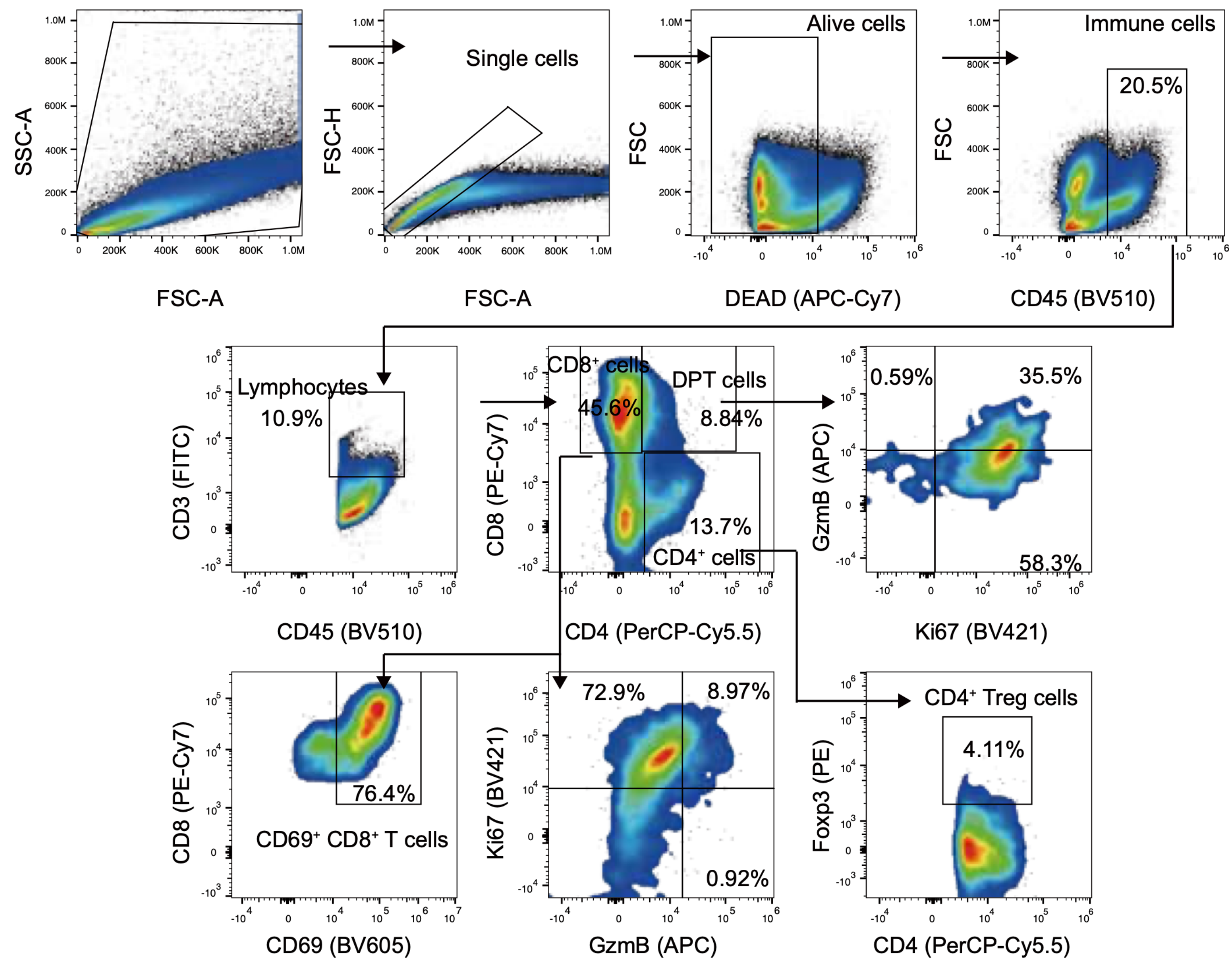

Fig. S13. Representative gating strategy for identifying lymphocytes with different phenotypes.

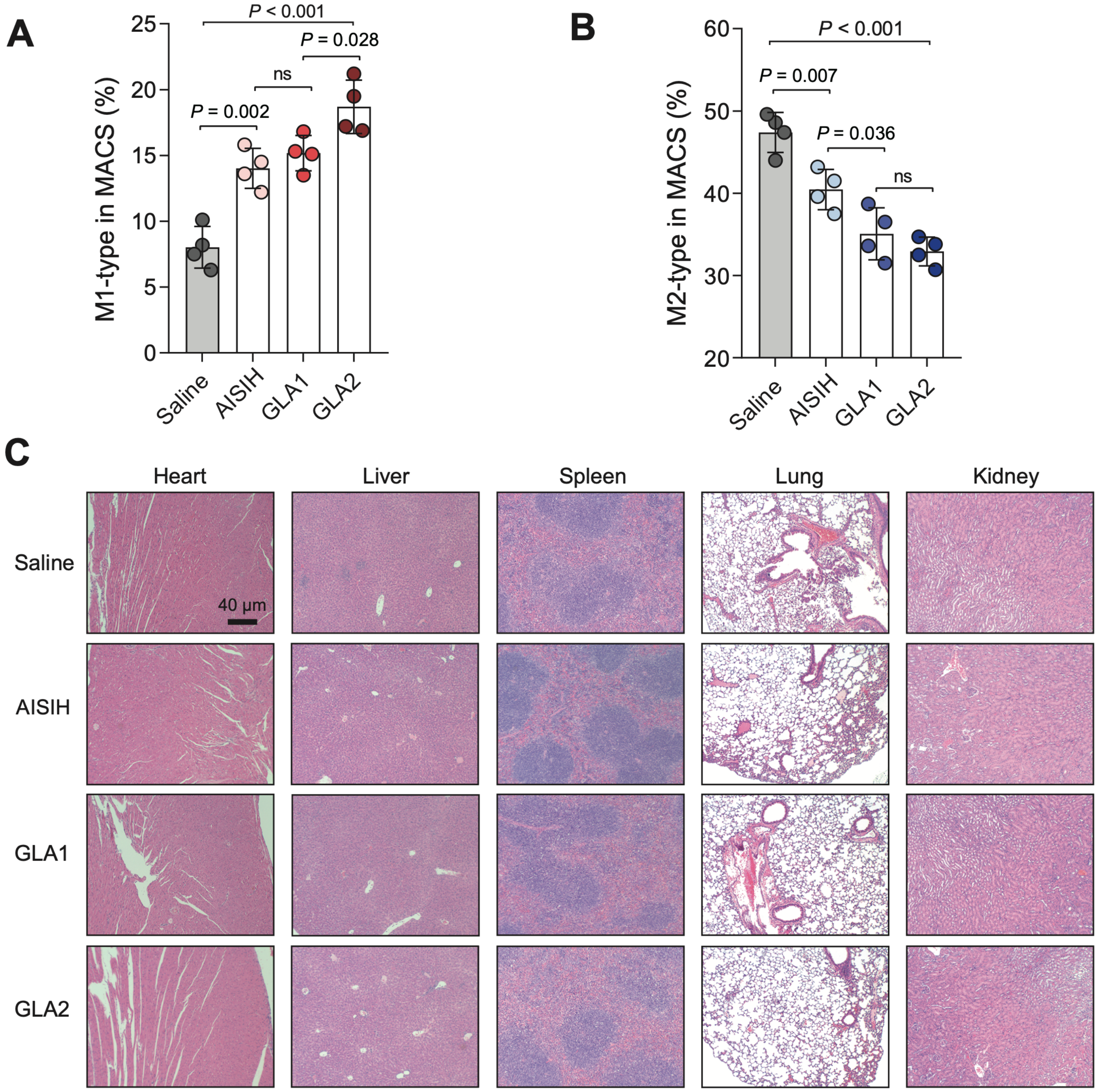

Fig. S14. The GLA2 strain modulates tumor-associated macrophages, exhibits excellent biosafety
(A) Changes in the frequency of M1-type macrophages ($CD86^+$) in tumors subjected to different treatments (n = 4). **(B)** Changes in the frequency of M2-type macrophages ($CD206^+$) in tumors subjected to different treatments (n = 4). **(C)** Close-up view of representative H&E-stained normal tissue, including heart, kidney, lung, and spleen sections, 7 days after different treatments (scale bar = 40 μm). The data are reported as the means ± SDs. All the data are representative of two independent experiments. ns, not significant. Abbreviations: H&E, Hematoxylin and Eosin staining; MACS, Multiply-Accumulate Operations; GLA1, glpX-deficient AISIH strain (Δ*glpX*-AISIH); GLA2, GLA1-derived strain expressing an anti-TNF-α nanoantibody under quorum-sensing control.

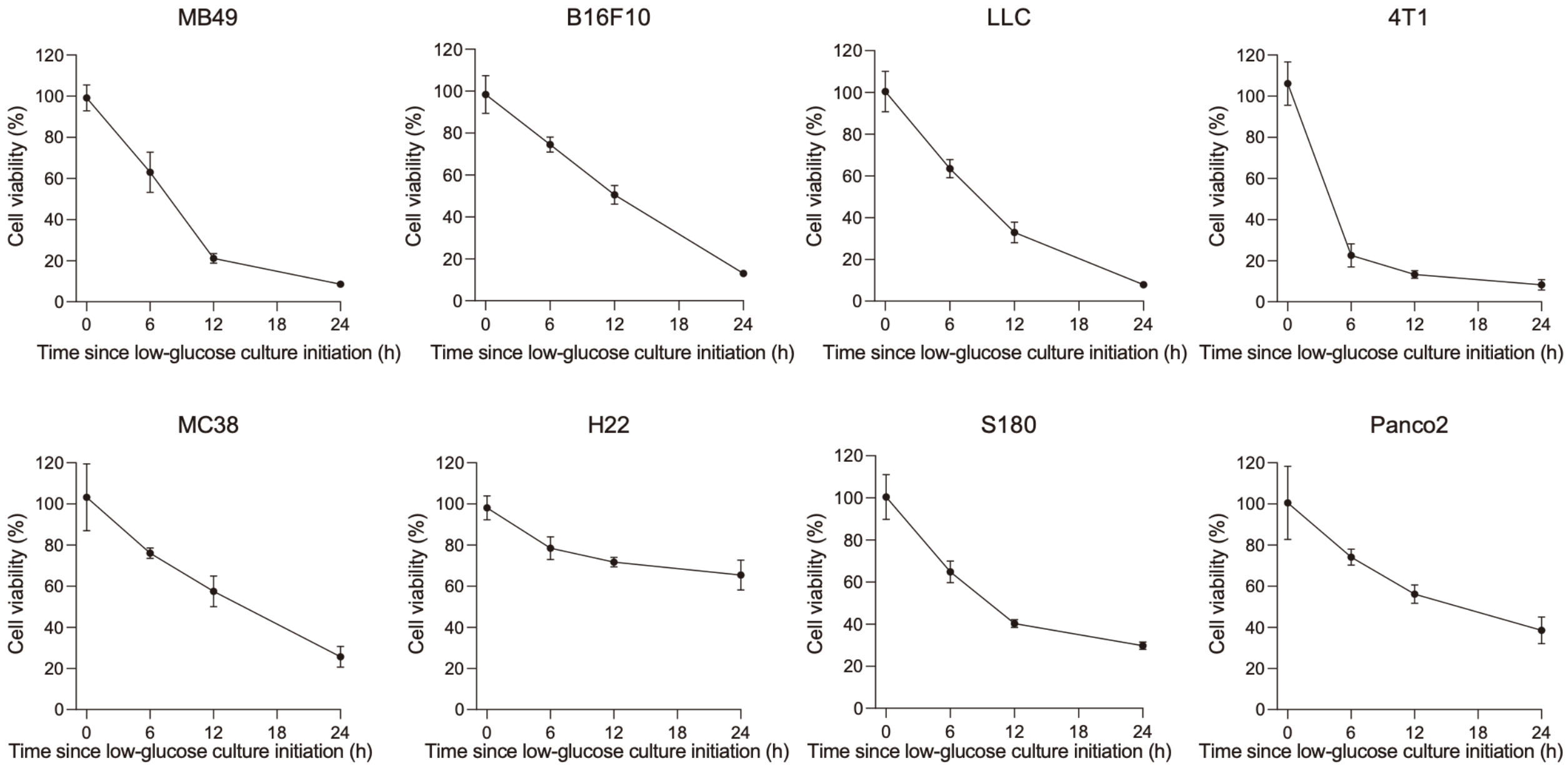

Fig. S15. The relative cell viability assay of different tumor cells after culturing under low-glucose (1 mmol/L) conditions for different times (*n* = 5). The CCK-8 was used to measure the cell viability at 0 h, 6 h, 12 h, and 24 h of culture. Cell viability = (OD_{450} measured under low-glucose conditions − OD_{450} of blank medium) / (OD_{450} measured under normal-glucose conditions − OD_{450} of blank medium) × 100%. Normal-glucose conditions: 25 mmol/L. The data are reported as the means ± SDs. All the data are representative of two independent experiments. Abbreviations: CCK-8, Cell Counting Kit-8.

Supplementary Tables

Table S1. List of the strains used in this study.

Strain #	Host Strain	Plasmid(s)	Description
**AISI**	Δ*htrA::LuxI*-VNP20009	N/A	Replacement of the *htrA* gene in AIS strain with the pJ23100-*LuxI* Seq.
**AISIH**	Δ*htrA::LuxI*-AISI	pTD103 pLuxI-htrA	AISI strains express HtrA in a QS-dependent manner
**AISIH-NC**	Δ*htrA::LuxI*-AISIH	pTh23 empty	AISIH strains carrying empty plasmids
**AISIH-2**	Δ*htrA::LuxI*-AISIH	pTh23 pJ23100-RFP	AISH strains constitutively express RFP
**AISIH-3**	Δ*htrA::LuxI*-AISIH	pTD103 pLuxI-htrA-LuxCDABE	AISIH strains express LuxCDABE in a QS-dependent manner
**AISIH-psifB-Lyse**	Δ*htrA::LuxI*-AISIH	pTh23 psifB-Lyse	AISIH strains express Lyse under the regulation of the sifB promoter
**Δ*yidA***	Δ*yidA*-AISIH	N/A	AISIH strains defective in *yidA* gene
**Δ*yigL***	Δ*yigL*-AISIH	N/A	AISIH strains defective in *yigL* gene
**Δ*glpX* (GLA1)**	Δ*glpX*-AISIH	N/A	AISIH strains defective in *glpX* gene
**Δ*ppsA***	Δ*ppsA*-AISIH	N/A	AISIH strains defective in *ppsA* gene
**Δ*ptsG***	Δ*ptsG*-AISIH	N/A	AISIH strains defective in *ptsG* gene
**Δ*pfkB***	Δ*pfkB*-AISIH	N/A	AISIH strains defective in *pfkB* gene
**Δ*pykF***	Δ*pykF*-AISIH	N/A	AISIH strains defective in *pykF* gene
**Δ*cra***	Δ*cra*-AISIH	N/A	AISIH strains defective in *cra* gene
**GLA2**	Δ*glpX*-AISIH	pTD103 pLuxI-htrA-TNF-αnb	GLA1 strains express TNF-αnb in a QS-dependent manner

Abbreviations: *ptsG,* phosphotransferase system glucose-specific transporter component; *pfkB*, phosphofructokinase B; *pykF*, pyruvate kinase F; *cra*, catabolite repressor/activator; *yidA*, putative phosphatase YidA; *yigL*, haloacid dehalogenase-like hydrolase YigL; *glpX*, fructose-1,6-bisphosphatase II; *ppsA*, phosphoenolpyruvate synthase; TNF-α, tumor necrosis factor-alpha; QS, Quorum sensing.

Table S2. List of the primers used for qPCR and PCR in this study.

Gene	Primer sequence	Primer length
**β-actin**	Forward-5′-GCA CCA CAC CTT CTA CAA TGA G-3′	22
	Reverse-5′-TTG GC ATA GAG GTC TTT ACG GA-3′	22
Il6	Forward-5′- TAG TCC TTC CTA CCC CAA TTT CC-3′	23
	Forward-5′- TTG GTC CTT AGC CAC TCC TTC-3′	21
glpX	Forward-5′- CCA GGT TTT AGC GCC G -3′	16
	Reverse-5′- CAG ATA TTC TCG GCA CGC -3′	18
yigL	Forward-5′- CGA GCC GCA GCT GCG C -3′	16
	Reverse-5′- AGC AGT TCG GAT ACG G -3′	16
yidA	Forward-5′- GCT GGC AGC GCC GAA C -3′	16
	Reverse-5′- CTT TTC CTG GTT GGG G -3′	16
ppsA	Forward-5′- GAG GTT GCG CAA TGA AAT GC -3′	20
	Reverse-5′- CGG GCC GAA CGT TTT TAT G -3′	19
cra	Forward-5′- TGT AGG CTG GAG CTG CTT C-3′	19
	Reverse-5′- CAT ATG AAT ATC CTC CTT AG-3′	19
pfkB	Forward-5′- TAT GAA TAT CCT CCT TAG -3′	18
	Reverse-5′- GTG TAG GCT GGA GCT GCT TC -3′	20
ptsG	Forward-5′- GTG TAG GCT GGA GCT GCT TC -3′	20
	Reverse-5′- TAT GAA TAT CCT CCT TAG -3′	18
pykF	Forward-5′- GTA GGC TGG AGC TGC TTC -3′	18
	Reverse-5′- CAT ATG AAT ATC CTC CTT AG -3′	20
Slc2a1	Forward-5′- GCA GCA AGA CCG ATG AAC AC -3′	20
	Reverse-5′- TAG CCG AAC TGC AGT GAT CC -3′	20
Slc2a4	Forward-5′- GCT CTG ACG TAA GGA TGG GGA-3′	21
	Reverse-5′- GTA GTG AGG GTG CCT TGT GG-3′	20
Pfkp	Forward-5′- AAC GCA CCC TAC CTG GAA AG-3′	20
	Reverse-5′- CAG GGC AGT ATC ACA ACC GA-3′	20
Hk2	Forward-5′- TGA GTG AAG TTA GCG ACG GG-3′	20
	Reverse-5′- CTG TCC GAC CGC TTT GAA AC-3′	20
Ccnb1	Forward-5′- TAA GGC CGT GAC AAA GGC AT-3′	20
	Reverse-5′- GAC AAC TTC CGT TAG CCT-3′	18
Cox6b1	Forward-5′- TTG GCG TCT TTG CTA AGG GT-3′	20
	Reverse-5′- TTC TCA CAG CGG TGG AAG TC-3′	20
Ifnb1	Forward-5′- CCA GCT CCA AGA AAG GAC GA-3′	20
	Reverse-5′- AGT CTC ATT CCA CCC AGT GC-3′	20
Cxcl10	Forward-5′- GAG AGA CAT CCC GAG CCA AC-3′	20
	Reverse-5′- TCA ACA CGT GGG CAG GAT AG-3′	20
mtNd1	Forward-5′- CTA GCA GAA ACA AAC CGG GC-3′	20
	Reverse-5′- CCG GCT GCG TAT TCT ACG TT-3′	20
CytB	Forward-5′- CTT CGC TTT CCA CTT CAT CTT ACC-3′	23
	Reverse-5′- TTG GGT TGT TTG ATC CTG TTT CG-3′	23
Cox1	Forward-5′- TAT GTT CTA TCA ATG GGA GC-3′	20
	Reverse-5′- GTA GTC TGA GTA GCG TCG TG-3′	20

Abbreviations: β-actin, beta-actin; *Ccnb1*, cyclin B1; *Cox6b1*, cytochrome c oxidase subunit 6B1; *Cxcl10*, C-X-C motif chemokine ligand 10; *Cytb*, mitochondrially encoded cytochrome b; *Hk2*, hexokinase 2; *Ifnb1*, interferon beta 1; *Il6*, interleukin 6;*Cox1*, mitochondrially encoded cytochrome c oxidase 1; *mtNd1*, mitochondrially encoded NADH dehydrogenase 1; *Slc2a1*, solute carrier family 2 member 1; *Slc2a4*, solute carrier family 2 member 4; *cra*, DNA-binding transcriptional regulator; *yigL*, protein-tyrosine-phosphatase; *yidA*, sugar phosphatase YidA; *ptsG,* phosphotransferase system glucose-specific transporter component; *pfkB*, phosphofructokinase B; *pykF*, pyruvate kinase F; *cra*, catabolite repressor/activator; *yidA*, putative phosphatase YidA; *yigL*, haloacid dehalogenase-like hydrolase YigL; *glpX*, fructose-1,6-bisphosphatase II; *ppsA*, phosphoenolpyruvate synthase.

Table S3. List of the antibodies used for Western blotting and Flow cytometry in this study.

**Antibody name**	Manufacturer	Catalog number	Application	Dilution
p-STING	ABclonal	AP1580	Western blotting	1:500
STING	ABclonal	A23923	Western blotting	1:5,000
p-TBK1	Immunoway	YM8674	Western blotting	1:2,000
TBK	Immunoway	YT4561	Western blotting	1:500
p-IRF31	Immunoway	YM8566	Western blotting	1:1,000
IRF3	Immunoway	YT2398	Western blotting	1:5,000
GAPDH	Servicebio	GB15002-100	Western blotting	1:1,000
Tubulin	Servicebio	GB11017-100	Western blotting	1:1,000
anti-FLAG primary antibody	Sigma	F1804	Western blotting	1:2,000
HRP-conjugated goat anti-rabbit IgG secondary antibody	Servicebio	GB23301	Western blotting	1:10,000
HRP-conjugated goat anti-mouse IgG secondary antibody	Servicebio	GB23303	Western blotting	1:10,000
CD45-BV510	eBioscience	69-0451-82	Flow cytometry	1:100
CD3-FITC	BD Biosciences	561801	Flow cytometry	1:100
CD4-PERCP-CY5.5	BD Biosciences	550954	Flow cytometry	1:100
CD8-PE-CY7	BD Biosciences	552877	Flow cytometry	1:100
CD69-BV605	eBioscience	63-0691-82	Flow cytometry	1:100
CD11b-FITC	BD Biosciences	561684	Flow cytometry	1:100
CD11c-PE-CY7	BD Biosciences	558079	Flow cytometry	1:100
CD103-PERCP-CY5.5	eBioscience	46-1031-82	Flow cytometry	1:100
CD86-PE	Invitrogen	12-0862-82	Flow cytometry	1:100
F4/80-BV421	BD Biosciences	565411	Flow cytometry	1:100
GzmB-APC	Invitrogen	17-8898-82	Flow cytometry	1:50
Foxp3-PE	eBioscience	00-5523-00	Flow cytometry	1:50
Ki67-BV421	BD Biosciences	562899	Flow cytometry	1:50
CD206-APC	Invitrogen	17-2061-82	Flow cytometry	1:100
CD11c-APC	Selleck	G0093	Flow cytometry	1:100
CD80-FITC	Selleck	G0211	Flow cytometry	1:100
CD86-PE	Invitrogen	12-0862-82	Flow cytometry	1:100

Abbreviations: APC, allophycocyanin; BV, Brilliant Violet; CD, cluster of differentiation; FITC, fluorescein isothiocyanate; Foxp3, forkhead box P3; GAPDH, glyceraldehyde-3-phosphate dehydrogenase; GzmB, granzyme B; HRP, horseradish peroxidase; IgG, immunoglobulin G; IRF3, interferon regulatory factor 3; Ki67, marker of proliferation Ki-67; PE, phycoerythrin; PerCP-Cy5.5, peridinin chlorophyll protein-cyanine 5.5; p-IRF3, phosphorylated interferon regulatory factor 3; p-STING, phosphorylated stimulator of interferon genes; p-TBK1, phosphorylated TANK-binding kinase 1; STING, stimulator of interferon genes; TBK1, TANK-binding kinase 1; Tubulin, tubulin.
